# Triptycepenes: Synthesis, Metal Complexes, and Their Reactivity in Catalytic Reactions

**DOI:** 10.1021/acs.organomet.5c00102

**Published:** 2025-05-29

**Authors:** Shyam Sundar Mothuku, Uwe Monkowius, Marko Hapke

**Affiliations:** † Institute for Catalysis (INCA), 27266 Johannes Kepler University Linz (JKU), Altenberger Strasse 69, A-4040 Linz, Austria; ‡ Institute of Inorganic Chemistry (IAC), JKU Linz, Altenberger Strasse 69, A-4040 Linz, Austria; § Institute for Catalysis e.V. (LIKAT), Albert-Einstein-Strasse 29a, D-18059 Rostock, Germany

## Abstract

An efficient protocol allows the convenient synthesis of substituted triptycepenes through the condensation of anthracene α-diketone with 3-pentanone or 1,3-diphenylpropan-2-one, finally yielding the corresponding cyclopentadienones. This approach offers a potential route toward sterically bulky cyclopentadienyl ligand frameworks. The methyl-substituted triptycepene was used to generate η^5^-coordinated Ru and Co complexes as well as an η^4^-coordinated Fe complex. These metal complexes were characterized by single-crystal X-ray diffraction, and their exemplary catalytic activity in metal-catalyzed reactions like cyclotrimerization, C–H functionalization, and transfer hydrogenation was investigated.

## Graphical abstract

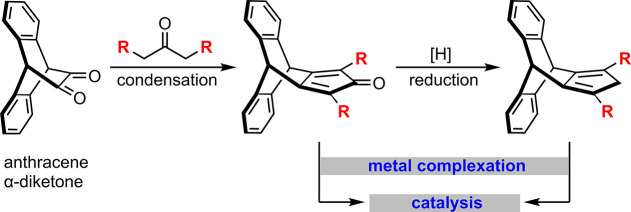

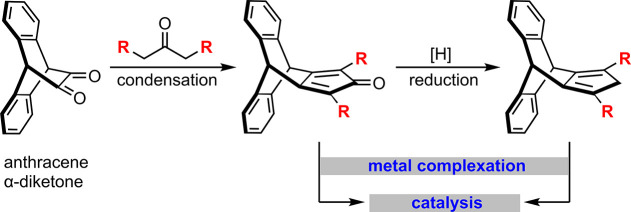

## Introduction

Triptycenes are hydrocarbon molecules from the iptycene family, distinguished by arene rings connected via a [2.2.2]­bicyclooctane skeleton with *D*_3*h*_ molecular symmetry. Initially, research on triptycenes centered on synthetic aspects. Later, their unique, rigid three-dimensional structure has gradually led to potential applications across various fields. In recent years, triptycene derivatives have been used for the synthesis of mechanically interlocked molecules,^1^ self-assembly, and molecular recognition in supramolecular chemistry.^2^ Additionally, they find applications as anticancer agents,^3^ models for studying hindered rotation,^4^ and structural components in material science.^5^ Triptycenes also serve as key units in functional polymers, porous materials,^6^ molecular cages,^7^ metal–organic frameworks,^8^ chemical sensors,^9^ and liquid crystals.^10^

Triptycene (**A**) itself is an achiral molecule (Figure 1a), but its derivatives can display inherent chirality depending on the substitution pattern.^11^ For example, when identical substituents are positioned at the 1,5- or 2,6-positions, the two bridgehead carbon atoms form asymmetric centers, resulting in a *C*_2_ symmetry axis. Achiral triptycenes have primarily been used as ligands or catalysts incorporating metals, particularly in chelating ligand complexes for organic transformations.^12^ In recent years, the rigid structure and unique properties of inherently chiral triptycene scaffolds have gained considerable attention across various fields.^13^ Compared with other *C*_2_-symmetrical chiral synthons, such as 1,1′-binaphthyl, *trans*-1,2-disubstituted cycloalkanes, and 1,1,2,2-tetrasubstituted ethane-based scaffolds, triptycenes offer exceptional features for innovative molecular design. These include a robust chiral backbone and highly restricted conformational flexibility, making them particularly appealing for advanced functional applications. Triptycene (**A**) derivatives have therefore been widely studied and recognized as essential synthons for various applications in materials science, supramolecular chemistry, and as achiral or chiral ligands in catalysis. In contrast, the chemistry of triptycepene (**B**) has received comparatively less attention (Figure 1a). It was first reported in 1977, and to the best of our knowledge, no attempts have been made to synthesize its substituted derivatives later.^14^

**Figure 1:**
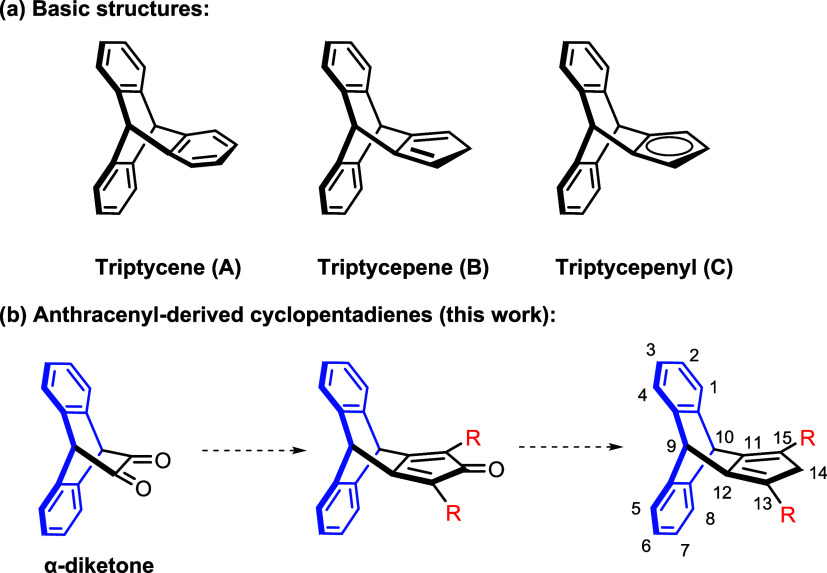
(a) Representative structures of triptycene (**A**) and triptycepene (**B**) and (b) the outline of triptycepene synthesis.

In the present study, we therefore focus on symmetrically ring-substituted triptycepene (**B**) (Figure 1b), which can be imagined as a propeller-like compound with an anionic cyclopentadienyl group instead of another arene like in **A** (Figure 1a). Simply starting out from anthracene by a Diels–Alder reaction, the construction of the cyclopentadiene fragment using a condensation procedure was considered the crucial reaction step (Figure 1b). It should allow us to access a stable cyclopentadienyl ligand possessing a sterically shielding backbone provided by the anthracene fragment (Figure 1a, general structure **C**). Further decoration of the Cp ring by condensation reactions opens up new prospects to improve the steric and electronic properties of the target cyclopentadienyl ligands.

## Results and Discussion

At the outset of our study, we started with the synthesis of the required anthracene backbone precursor as outlined in Scheme 1, in accordance with literature precedent. The α-diketone **5** can be easily prepared by the Diels–Alder reaction of anthracene **1** and vinylene carbonate **2**, and several variations of this approach to **3** have since emerged.^15^ In the present case, we performed the [4 + 2] cycloaddition reaction without the requirement of a large excess of dienophile **2**. The subsequent transformation of carbonate **3** to diol **4**, using sodium hydroxide or potassium hydroxide in water or aqueous ethanol, has also been reported.^16^ The hydrolysis was performed using aqueous sodium hydroxide in dioxane under reflux conditions, yielding the diol in 93% yield. Finally, we employed the Swern oxidation procedure to cleanly oxidize diol **4** to diketone **5** with 77% yield.

**Scheme 1:**
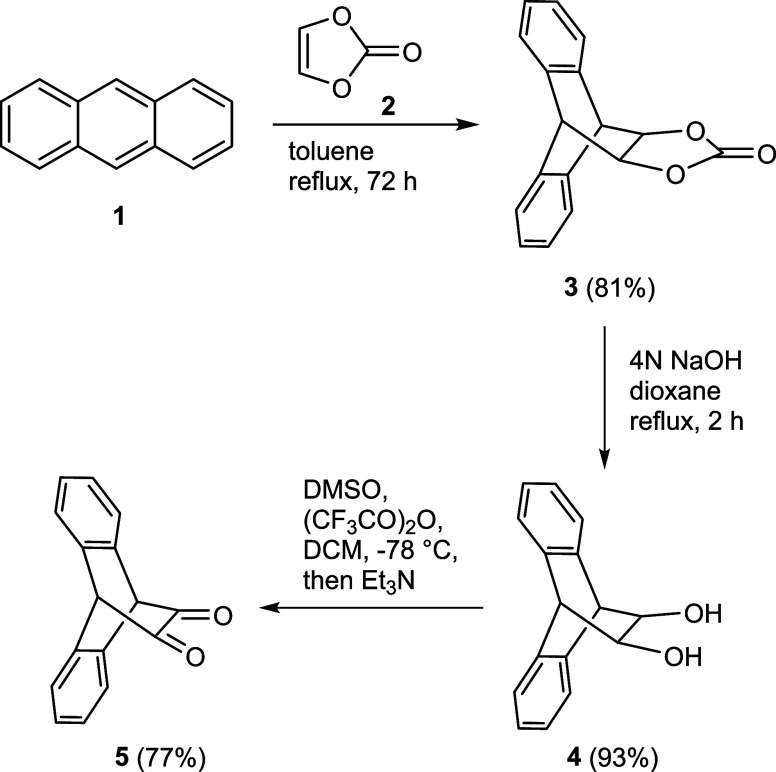
Synthesis of α-Diketone **5**

To obtain the target compound, the key step in our synthetic strategy was the subsequent condensation reaction of α-diketone **5**. Generally, the condensation reactions in the presence of a base are well-known in the literature.^17^ Therefore, we subjected the condensation of **5** with 3-pentanone **6** or 1,3-diphenylpropan-2-one **10** in the presence of a base to afford the intermediate hydroxy-cyclopentenone **7** with 92% yield or **11** with 75% yield (Scheme 2). Subsequently, employing a widely used method,^18^ an acid-catalyzed dehydration in acetic anhydride was pursued to obtain the cyclopentadienone. The elimination reaction of the hydroxy compound **7** produced cyclopentadienone **8** with 61% yield and in addition a side product (for details see the Supporting Information). In the case of **11**, we exclusively isolated product **12** in 68% yield. Finally, the reduction of cyclopentadienone **8** was carried out following the literature procedure using AlCl_3_–LiAlH_4_,^19^ furnishing the final product **9** with 71% yield along with hydroxy byproduct formation (for details see the Supporting Information). Following this procedure, compound **12** was smoothly reduced to **13** with 91% yield (Scheme 2). Release of hydrogen during the procedure by connection to a Schlenk line with a bubbler proved to be advantageous.

**Scheme 2:**
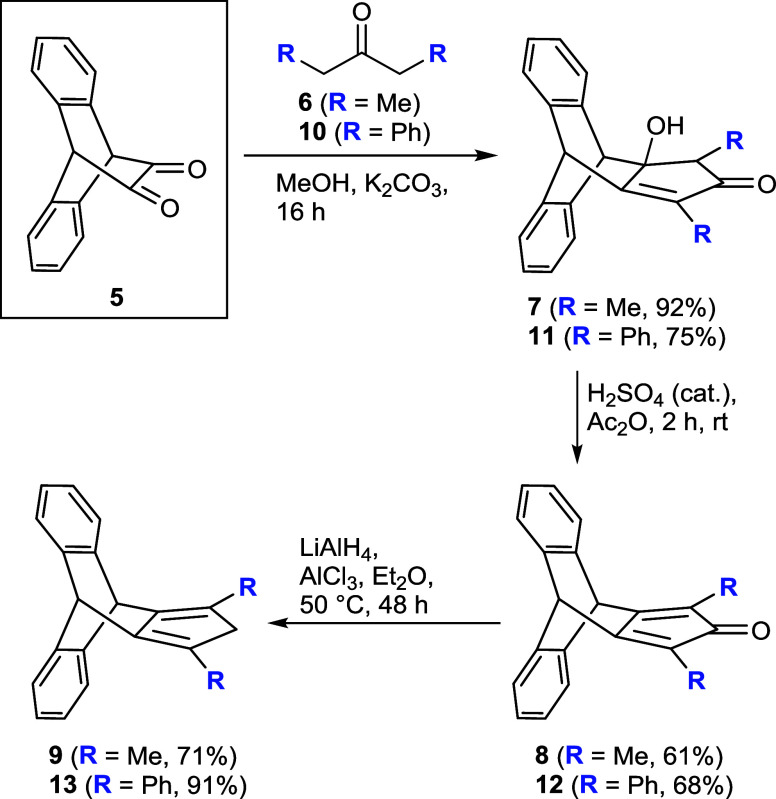
Synthesis of Substituted Triptycepenes **9** and **13**

Single-crystal X-ray diffraction studies confirmed the molecular structure of **9**. A suitable crystal was grown by the slow evaporation of its *n*-hexane solution. The compound crystallizes in the space group *C*2/*c*, and the asymmetric unit contains half a molecule (Figure 2).

**Figure 2:**
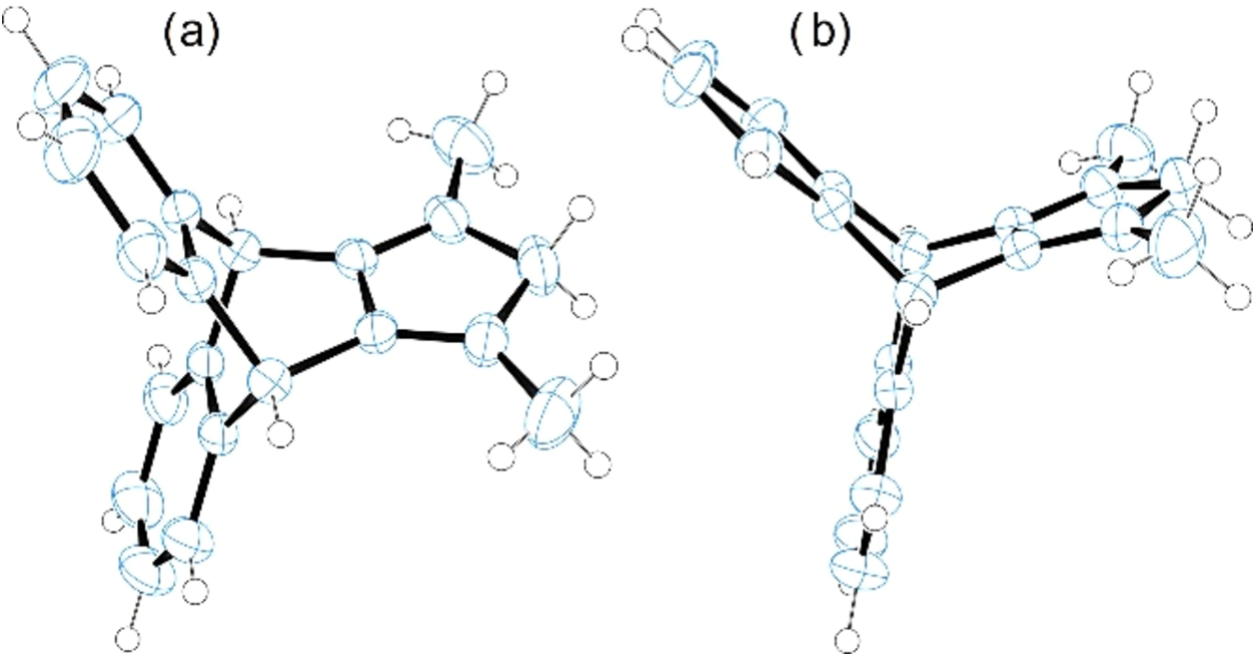
Molecular structure of **9** (CCDC 2423988), (a) upper view and (b) side view.

Next, we shifted our attention to synthesizing metal complexes from either the cyclopentadienone **8** or the cyclopentadiene **9**. Initially, we synthesized a ruthenium complex using a well-established one-pot synthetic route,^20^ which yielded the benzoyloxy-substituted ruthenium complex **Ru-14** by reacting **8** first with a ruthenium carbonyl compound and subsequently with benzoyl chloride (Scheme 3). Second, we prepared an iron complex using an established procedure,^21^ where the reaction of cyclopentadienone **8** with Fe­(CO)_5_ furnished the iron complex **Fe-15** in good yield (Scheme 3). The complex structures were confirmed by single-crystal X-ray diffraction (XRD) analyses (Scheme 3). In complex **Ru-14**, the ruthenium center showed η^5^-coordination with the cyclopentadienyl ligand. The two Ru-CO bond lengths (1.904 and 1.880 Å) and the Ru–Cl bond length (2.408 Å) are consistent with values reported for Cp-Ru complexes in previous studies.^22^ Interestingly, the distance from the ruthenium atom to the Cp plane (Ru–C_5_ centroid) shows a slight increase from 1.875 to 1.887 Å compared to the reported data. In complex **Fe-15**, the iron(0) center is coordinated by a η^4^-bound pentadienone ring. The η^4^-coordination is confirmed by four Fe-diene carbon atom distances ranging in a similar range from approximately 2.071–2.127 Å and the Fe–C­(O) distance of 2.382 Å, being consistent with literature values.^23^ As compared to the classical Knölker complex,^24^ the Fe-diene carbon atoms’ bond length decreases from an average of 1.798 Å to an average of 1.760 Å in **Fe-15**. The distance of the Fe–C_5_ centroid increases from 1.722 to 1.763 Å. In the infrared (IR) spectrum, the terminal CO ligand stretching frequency decreased from 2053 and 1989 cm^–1^ to 2050 and 1979 cm^–1^ in **Fe-15** and the cyclopentadienone carbonyl stretching frequency increased from 1608 to 1627 cm^–1^ in **Fe-15**.

**Scheme 3:**
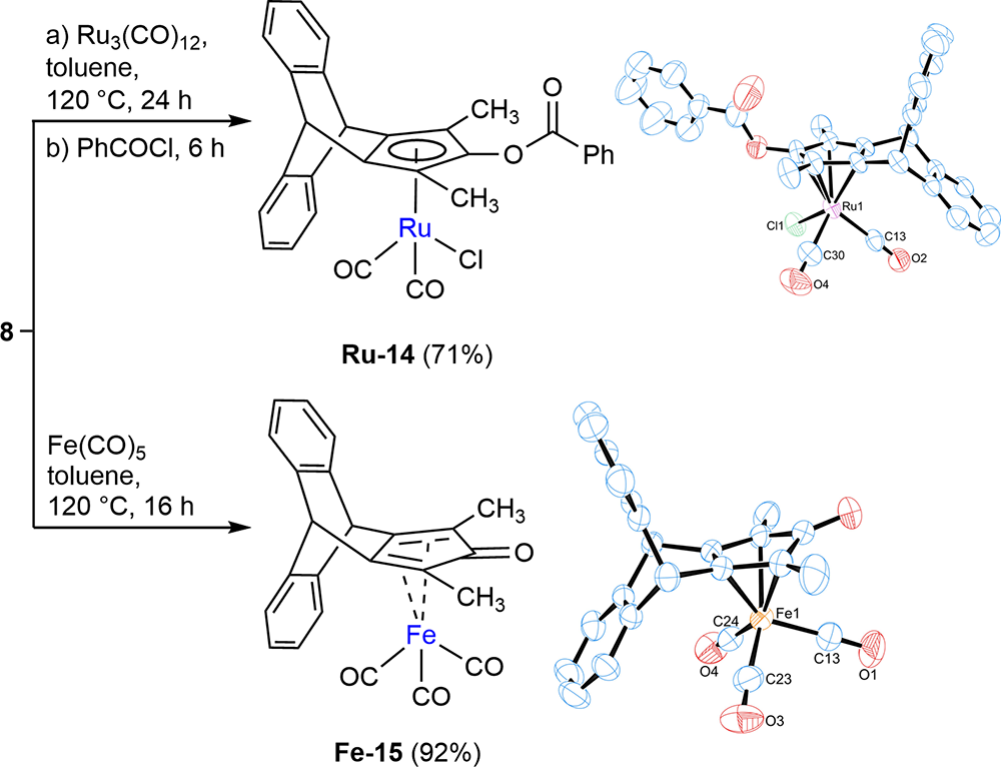
Synthesis of Ru and Fe Complexes from Cyclopentadienone **8**

In the next step, we successfully synthesized the cobalt complex by reacting cyclopentadiene **9** with Co_2_(CO)_8_ under mild conditions (Scheme 4). Since the CpCo­(I)-carbonyl complexes are usually not easy to purify, we oxidized the primary reaction product with elemental iodine, leading to the final reaction product **Co-16**, which was possible to purify using silica gel column chromatography on air. The complex structure was also confirmed by single-crystal XRD analysis (Scheme 4). The crystal structure showed η^5^-coordination between the cobalt center and the cyclopentadienyl ligand. As compared to the CpCoI_2_(CO) complex,^25^ the Co-CO bond length increases from 1.76 Å in CpCoI_2_(CO) to 1.778 Å in **Co-16**, and the distance of the Co–C_5_ centroid increases from 1.68 to 1.708 Å. In the IR spectrum, the carbonyl (CO) stretching frequency decreased from 2060 to 2038 cm^–1^ in **Co-16** due to the electron-donating effect of the alkyl substituents on the Cp ring. We also attempted to prepare a ruthenium complex starting from **9**, following the literature procedures.^26^ However, the reaction with Ru_3_(CO)_12_ and RuCl_3_ without the presence of a base produced complex mixtures along with the starting material, and no significant single complexation product could be isolated.

**Scheme 4:**
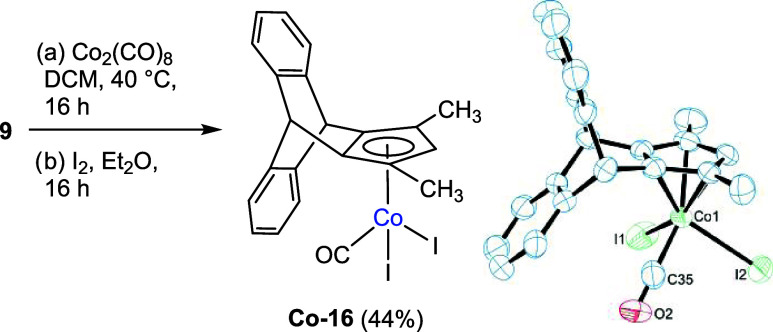
Synthesis of the CpCo­(III) Complex with Cyclopentadiene **9**

After the successful isolation of the metal complexes, we set out to study the reactivity of all the newly synthesized complexes in metal-catalyzed reactions. We first selected the ruthenium complex **Ru-14** for the cyclotrimerization of triynes **17** as a benchmarking reaction. Ruthenium complexes are well-known to catalyze [2+2+2] cycloaddition reactions under mild conditions, most often catalyzed by Cp*RuCl­(COD) or derivatives thereof.^27^ We investigated the reaction under thermal as well as photochemical conditions, with the latter one being rarely studied with ruthenium complexes in the reaction setup (Scheme 5). While at room temperature, a very slow conversion of triyne **17a** was observed, the reaction on heating to 120 °C gave **18a** in excellent 96% yield. On the contrary, light irradiation at 365 nm wavelength in a photochemical reactor gave 88% yield even at room temperature. However, when the reaction was carried out at light irradiation with a longer wavelength (450 nm), only minimal product formation was observed and the starting material was recovered. It was reported that the cyclization of the terminally unsubstituted triyne **17b** under thermal conditions using Cp*RuCl­(COD) as catalyst gave 32% yield of the desired product **18b** along with a byproduct.^28^ In contrast, our catalyst **Ru-14** furnished product **18b** in 82% yield and with an excellent 94% yield under photochemical conditions, a clear improvement over the conventional catalyst. Finally, triyne **17c** yielded the cyclized product **18c** with high efficiency under thermal (93%) and photochemical (86%) conditions, as well. The obtained results demonstrated that precatalyst **Ru-14** was highly active, resulting in up to 96% isolated yields for intermolecular cyclotrimerization products (Scheme 5).

**Scheme 5:**
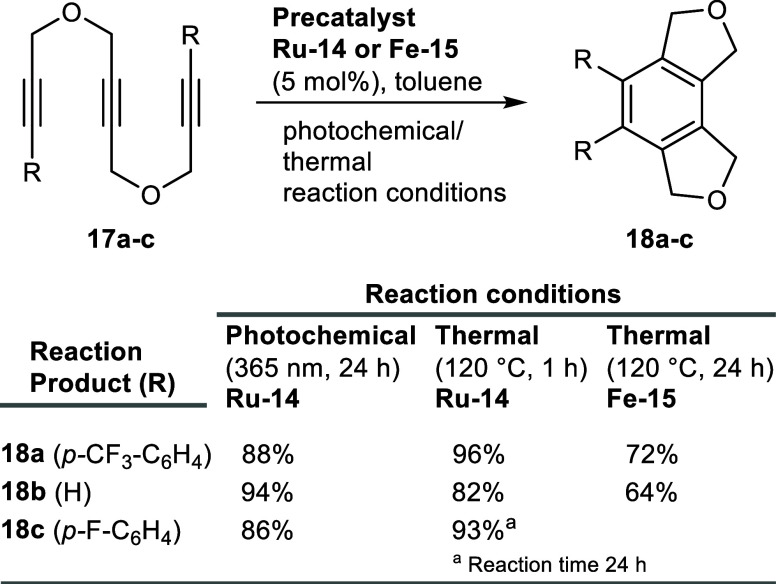
Reactivity Screening for Precatalyst Ru-14 in the Cyclotrimerization of Triynes **17a**–**c** under Thermal and Photochemical Conditions

Second, we evaluated the catalytic reactivity of complex **Fe-15** in carbonyl reductions. The (cyclopentadienone)­iron tricarbonyl compounds are known for reducing carbonyl compounds under transfer hydrogenation conditions.^29^ The substrate scope and potential limitations of the reaction were tested with acetophenone and standard derivatives that bear either electron-donating (**20c**) or electron-withdrawing groups (**20d**). All the reactions were carried out under 30 bar H_2_ pressure at 80 °C, using Me_3_NO to activate the precatalysts in situ.^30^ The obtained results show that catalyst **Fe-15** was highly active, resulting in at least 91% conversion and excellent isolated yields of the alcohols (Scheme 6), similar to the other substituted (cyclopentadienone)iron tricarbonyl complexes under identical conditions.^31^

**Scheme 6:**
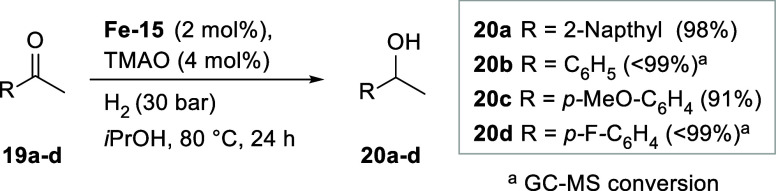
Reactivity Screening for Precatalyst **Fe-15** in Transfer Hydrogenation Reactions

Out of curiosity and since iron-catalyzed [2+2+2] cyclotrimerization reactions are known in the literature,^32^ we also wanted to evaluate our iron Knölker-type complex for the cyclotrimerization reaction, which has not been reported yet to the best of our knowledge. Complex **Fe-15** was therefore tested in cyclotrimerization reactions using compounds **17a** and **17b** as substrates, under thermal conditions for 24 h without activator, furnishing cyclized products **18a** (72%) and **18b** (64%) in surprisingly good yields, respectively (Scheme 5).

Finally, we evaluated the catalytic reactivity of complex Co-**16** for the C–H functionalizations of chlorobenzamides **21** to synthesize dihydroisoquinolones **23**. Previous studies have demonstrated this transformation using achiral Cp*Co catalysts^33^ and chiral Cp^x^Co^34^ systems. Compared to literature protocols mostly using Cp*CoI_2_(CO),^33^ the catalyst **Co-16** also performs effectively and delivered high yields even at a reduced catalyst loading of 2 mol % instead of 5 mol % or even 10 mol % loading, which were often applied (Scheme 7).^35^ We generally investigated the reaction scope with styrenes bearing either electron-donating (**22b**) or electron-withdrawing groups (**22c**), in which cases dihydroquinolones **23b** and **23c** were also obtained in good yields (Scheme 7).

**Scheme 7:**
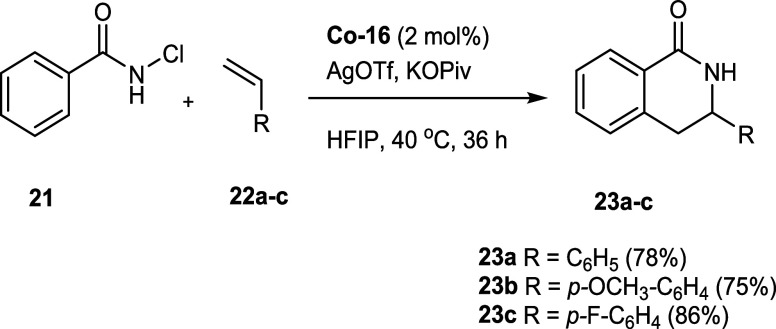
Reactivity Screening for Precatalyst **Co-16** for C–H Functionalizations of *N*-Chlorobenzamides and Styrenes

Besides the rather simple synthesis of the Cp ligand precursors containing a large shielding aromatic backbone and their metal complexation, the evaluation of the catalytic properties of selected metal complexes demonstrated the potential of these structures for a variety of transformations, which we aim to exploit in further studies.

## Conclusions

In conclusion, we have successfully designed and synthesized so far unknown substituted triptycepene compounds, starting by simple transformations from anthracene. A key step for the cyclopentadiene ring construction was a double condensation reaction. The synthetic route allows for easy modification within the Cp-ring-substituted triptycepene framework. Furthermore, triptycepene-based metal complexes exhibited excellent reactivity in C–H activation, cyclotrimerization, and reductive transfer hydrogenation reactions.

## Experimental Section

### General Methods

All experiments were carried out under an inert gas atmosphere in flame-dried Schlenk tubes or glass reaction vials using a standard Schlenk-line technique, unless stated otherwise. The anhydrous solvents (dichloromethane, diethylether, tetrahydrofuran, and toluene) were dried in a solvent purification system MB SPS7 from MBraun and degassed via three cycles of freeze–pump–thaw. All other chemicals (Aldrich, Aurora Fine Chemicals LLC, BLDPharm, Chempur Chemicals, Fisher Scientific, Fluorochem, Merck, and TCI) were purchased and used as received. All reactions were heated by using a temperature-controlled oil bath and a magnetic stirrer. Photochemical reactions were conducted in a HepatoChem Lucent 360 photoreactor or in an air-cooled EvoluChem PhotoRedOx Box (equipped with a 30 W LED) with LEDs of given wavelength.

Thin-layer chromatography was performed on Merck 60 F254 silica gel plates or ALUGRAM ALOX N/UV 254 plates with a layer size of 0.2 mm from MACHERY-NAGEL. Column chromatography utilized Silica 60 with 0.04–0.063 mm particle size.

NMR spectroscopy was performed on a Bruker Avance 300 MHz spectrometer (^1^H: 300 MHz, ^13^C: 75 MHz, ^19^F: 282 MHz). Chemical shifts are stated in parts per million (ppm) on a delta scale (δ). Axis calibration was performed using the residual protic solvent signals (^1^H NMR: CDCl_3_: 7.26 ppm, ^13^C NMR: CDCl_3_: 77.16 ppm). Multiplicities are stated as s (singlet), d (doublet), dd (doublet of doublets), dt (doublet of triplets), t (triplet), or m (multiplet). High-resolution mass spectrometry was carried out on an Agilent QTOF 6520 instrument with electrospray ionization (ESI). GC-MS analysis was performed on a Shimadzu GC-MS QP 2020 instrument using helium (purity 5.0) from Linde Gas GmbH as carrier gas. Melting points were determined with the Büchi Melting Point M-560.

Compounds **17a**–**c** ^36^ and compound **21** ^34^ ^b^ were prepared according to literature procedures.

#### Synthesis of Compound **7**

To a suspension of potassium carbonate (2.36 g, 17.1 mmol) in dry methanol (25 mL) was added pentan-3-one **6** (1.47 g, 1.81 mL, 17.1 mmol) at room temperature. Diketone **5** (2.00 g, 8.54 mmol, 1.0 equiv) was introduced portionwise over 15 min, and the reaction mixture was stirred at room temperature for 12 h. The reaction mixture was concentrated, and water was added. The yellow precipitate was extracted with CH_2_Cl_2_ (2 × 40 mL), and the combined organic layers were dried over Na_2_SO_4_. After filtration, the filtrate was concentrated in vacuo and dried to afford intermediate β-hydroxy ketone **7** as a yellow solid (2.38 g, 92%). Mp = 198–200 °C (decomp).

^1^H NMR (300 MHz, CDCl_3_): δ = 7.50–7.44 (m, 2H), 7.41–7.36 (m, 1H), 7.29–7.19 (m, 3H), 7.18–7.11 (m, 2H), 5.38 (s, 1H), 4.61 (s, 1H), 1.94 (q, *J* = 7.0, 1H), 1.78 (s, 3H), 1.47 (s, 1H), 1.26 (d, *J* = 7.0, 3H). ^13^C­{^1^H} NMR (75 MHz, CDCl_3_): δ 211.05, 170.25, 141.88, 140.74, 139.77, 137.95, 131.67, 127.87, 127.11, 127.08, 126.85, 126.74, 126.19, 125.28, 122.20, 79.81, 53.32, 52.93, 47.96, 8.51, 7.94. IR (neat, cm^–1^): 3070, 3022, 1747, 1706, 1675, 1590, 1579, 1460, 1332, 1318, 1303, 1284, 1227, 1170, 1119, 1066, 10368, 981, 935, 755, 694, 656. HRMS (ESI) calcd for C_21_H_18_O_2_ (M + H)^+^: 303.1380, found: 303.1383.
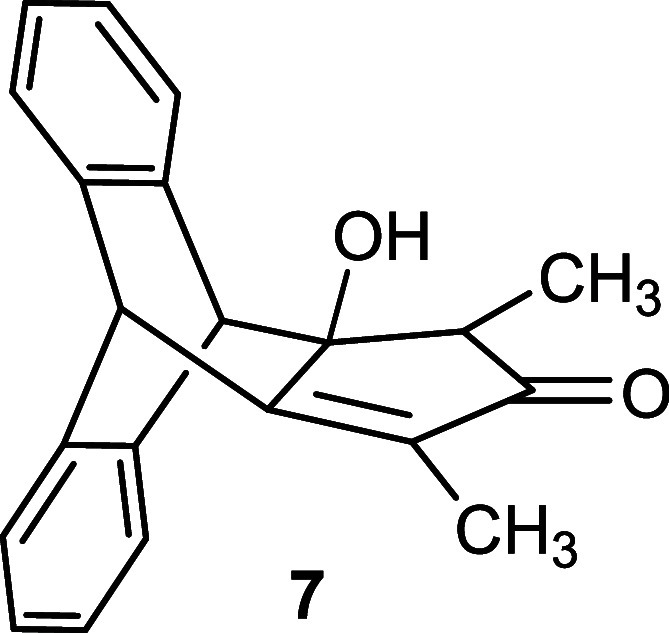


#### Synthesis of Compound **8**

The β-hydroxy ketone **7** (1.50 g, 4.96 mmol, 1.0 equiv) was suspended in Ac_2_O (20 mL) and H_2_SO_4_ (32 μL, 0.59 μmol) was added very slowly. The resulting brown solution was stirred at rt for 2 h, and then ice-cold water (50 mL) was slowly introduced. The mixture was stirred for 1 h, and the resulting orange precipitate was isolated. The precipitate was extracted with CH_2_Cl_2_ (2 × 30 mL), and the combined organic layers were dried over Na_2_SO_4_. After being filtered, the filtrate was concentrated in vacuo. The product was purified via column chromatography on silica gel (gradient: *n*-heptane/EtOAc = 0 → 40%) to afford 860 mg (61%) of compound **8** as an orange-red solid. Mp = 224–226 °C.

^1^H NMR (300 MHz, CDCl_3_) δ = 7.36 (dd, *J* = 5.4, 3.2, 4H), 7.22 (dd, *J* = 5.4, 3.2, 4H), 5.16 (s, 2H), 1.72 (s, 6H). ^13^C­{^1^H} NMR (75 MHz, CDCl_3_) δ: 209.33, 151.37, 139.54, 127.54, 124.31, 113.63, 46.20, 7.62. IR (neat, cm^–1^): 3065, 3030, 2986, 2931, 2807, 2844, 1703, 1655, 1456, 1443, 1429, 1277, 1206, 1151, 1044, 945, 817, 764, 743, 686, 625. HRMS (ESI) calcd for C_21_H_16_O (M + H)^+^: 285.1274, found: 285.1275.
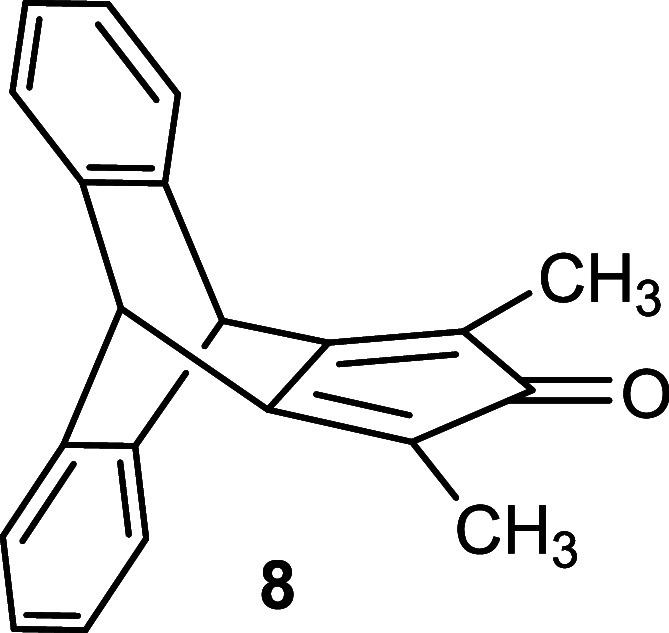


#### Synthesis of Compound **9**

A 100 mL Schlenk tube with PTFE cap was charged with anhydrous AlCl_3_ (0.47 g, 3.5 mmol, 4.0 equiv) in ether (5.0 mL). Lithium aluminum hydride (1 M in ether) (0.50 g, 13 mmol, 15 equiv) was slowly added to the reaction mixture at 0 °C, followed by compound **8** (0.25 g, 0.87 mmol, 1.0 equiv) in etheral solution (20 mL) and the reaction mixture was refluxed at 50 °C for 48 h. Then, water was added slowly to quench the reaction, and the organic layer was washed with brine and dried over Na_2_SO_4_. The solvent was removed in vacuo. The product was purified via column chromatography on silica gel (gradient: *n*-heptane/EtOAc = 0 → 5%) to afford compound **9** as a white solid (168 mg, 71%). Mp = 212–214 °C.

^1^H NMR (300 MHz, CDCl_3_): δ = 7.31 (dd, *J* = 5.3, 3.2, 4H), 7.06 (dd, *J* = 5.4, 3.2, 4H), 4.97 (s, 2H), 2.87 (s, 2H), 1.95 (s, 6H). ^13^C­{^1^H} NMR (75 MHz, CDCl_3_): δ 144.20, 141.64, 125.81, 125.25, 123.79, 52.16, 47.30, 13.30. IR (neat, cm^–1^): 3066, 3014, 2952, 2924, 2906, 2870, 2847, 1673, 1452, 1430, 1370, 1351, 1192, 1154, 873, 739, 723, 676, 634. HRMS (ESI) calcd for C_21_H_18_ (M + H)^+^: 271.1481, found: 271.1481.
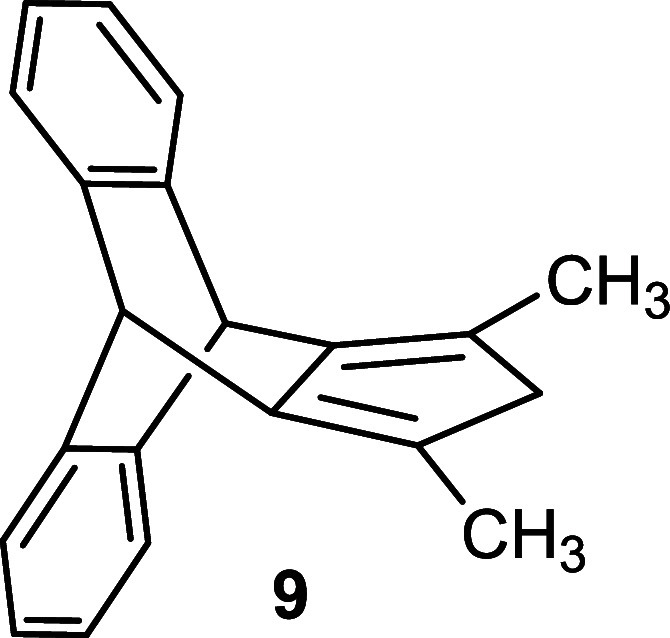


#### Synthesis of Compound **11**

Compound **11** was synthesized using the same procedure as for compound **7**. The product was obtained with 750 mg (75%) yield as a viscous liquid.

^1^H NMR (300 MHz, CDCl_3_) δ = 7.59–7.39 (m, 10H), 7.38–7.30 (m, 3H), 7.26–7.13 (m, 5H), 5.71 (s, 1H), 4.59 (s, 1H), 3.41 (s, 1H). ^13^C­{^1^H} NMR (75 MHz, CDCl_3_) δ 205.69, 169.78, 141.31, 140.44, 139.97, 137.73, 133.82, 131.04, 128.88, 128.78, 128.64, 128.61, 128.37, 128.04, 127.34, 127.23, 126.98, 126.23, 125.25, 123.18, 80.78, 53.22, 48.72.IR (neat, cm^–1^): 3551, 3066, 3023, 1714, 1496, 1459, 1223, 1103, 1077, 1031, 791, 766, 748, 720, 693. HRMS (ESI) calcd for C_31_H_23_O_2_ (M + H)^+^: 427.1693, found: 427.1694.
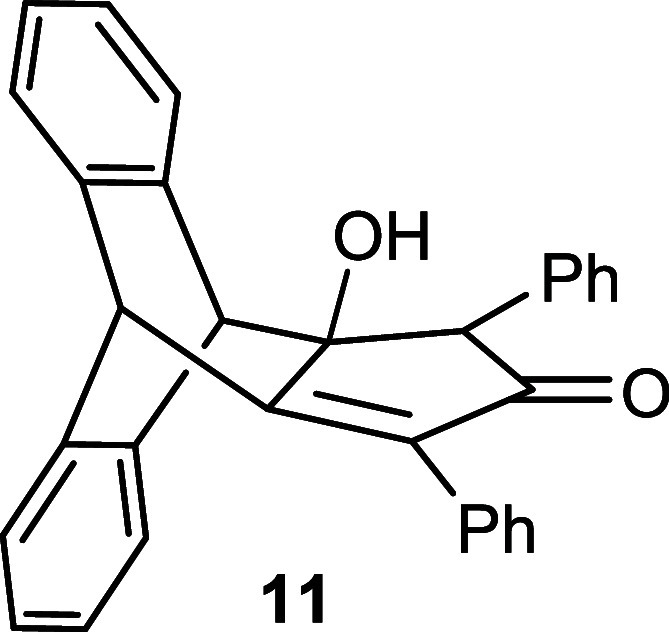


#### Synthesis of Compound **12**

Compound **12** was synthesized by the same procedure as for compound **8**. Yield: 324 mg (68%), Mp = 102–104 °C (decomp).

^1^H NMR (300 MHz, CDCl_3_): δ = 7.47–7.32 (m, 12H), 7.30–7.23 (m, 2H), 7.22–7.15 (m, 4H), 5.55 (s, 2H). ^13^C­{^1^H} NMR (75 MHz, CDCl_3_): δ 204.93, 153.54, 139.27, 131.14, 129.05, 128.73, 127.94, 127.64, 124.67, 117.34, 46.62. IR (neat, cm^–1^): 3024, 3012, 2955, 2916, 2848, 1733, 1709, 1491, 1460, 1444, 1375, 1278, 1239, 1157, 971, 791, 746, 691, 638. HRMS (ESI) calcd for C_31_H_21_O (M + H)^+^: 409.1587, found: 409.1588.
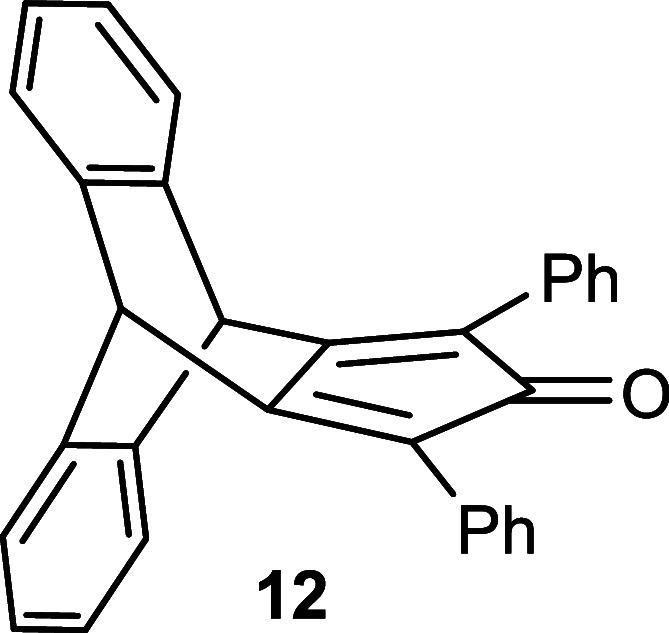


#### Synthesis of Compound **13**

Compound **13** was synthesized by the same procedure as for compound **9**. Yield: 53 mg (91%), Mp = 272–274 °C.

^1^H NMR (300 MHz, CDCl_3_) δ = 7.45–7.39 (m, 4H), 7.33 (ddd, *J* = 7.6, 5.0, 2.0, 8H), 7.21–7.14 (m, 2H), 7.02 (dd, *J* = 5.4, 3.2, 4H), 5.47 (s, 2H), 3.71 (s, 2H). ^13^C­{^1^H} NMR (75 MHz, CDCl_3_) δ: 145.22, 143.40, 136.82, 131.35, 128.82, 127.14, 126.51, 126.26, 124.21, 48.12, 47.61. IR (neat, cm^–1^): 3055, 3038, 3019, 2982, 2957, 2953, 1595, 1491, 1460, 1441, 1373, 1260, 1240, 1197, 1083, 1021, 800, 752, 742, 727, 692, 638, 545. HRMS (ESI) calcd for C_31_H_23_O_2_ (M + H)^+^: 395.1794, found: 395.1792.
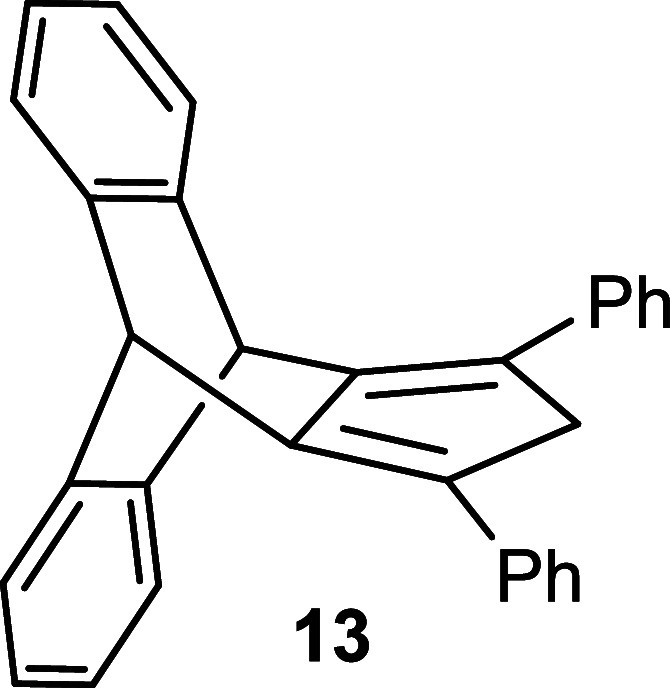


#### Synthesis of Ruthenium Complex **Ru-14**

A 25 mL Schlenk tube with PTFE cap was charged with compound **8** (100 mg, 0.35 mmol, 1.0 equiv) and Ru_3_(CO)_12_ (76.0 mg, 0.11 mmol, 0.33 equiv) and dissolved in toluene (5 mL) under argon. The mixture was heated to 120 °C and stirred for 24 h. After the starting material was consumed (monitored by TLC), benzoyl chloride (99.0 mg, 0.08 mL, 0.70 mmol, 2.0 equiv) was added and further stirred for 6 h at the same temperature. The volatiles were removed under reduced pressure, and the crude product was purified by column chromatography on silica gel (gradient: *n*-heptane/EtOAc = 10 → 25%) to afford 145 mg (71%) of **Ru-14** as a yellow solid. Mp = 230–232 °C (decomp).

^1^H NMR (300 MHz, CDCl_3_): δ = 8.17–8.10 (m, 2H), 7.68–7.59 (m, 1H), 7.51–7.44 (m, 2H), 7.33 (td, *J* = 5.3, 3.2,4H), 7.14 (dd, *J* = 5.4, 3.2, 2H), 7.06 (dd, *J* = 5.4, 3.2, 2H), 5.01 (s, 2H), 2.07 (s, 6H). ^13^C­{^1^H} NMR (75 MHz, CDCl_3_): δ = 195.64, 163.91, 145.22, 143.31, 134.60, 130.72, 128.94, 127.62, 126.74, 126.68, 126.65, 126.50, 124.44, 124.26, 123.72, 123.41, 107.23, 93.40, 45.37, 9.26. IR (neat, cm^–1^): 3021, 3015, 2957, 2923, 2852, 2042, 1987, 1747, 1454, 1253, 1243, 1229, 1144, 1053,1044, 1018, 740, 721, 708, 577, 551. HRMS (ESI) calcd for C_30_H_21_RuO_4_ (M – Cl)^+^: 547.0486, found: 547.0483.
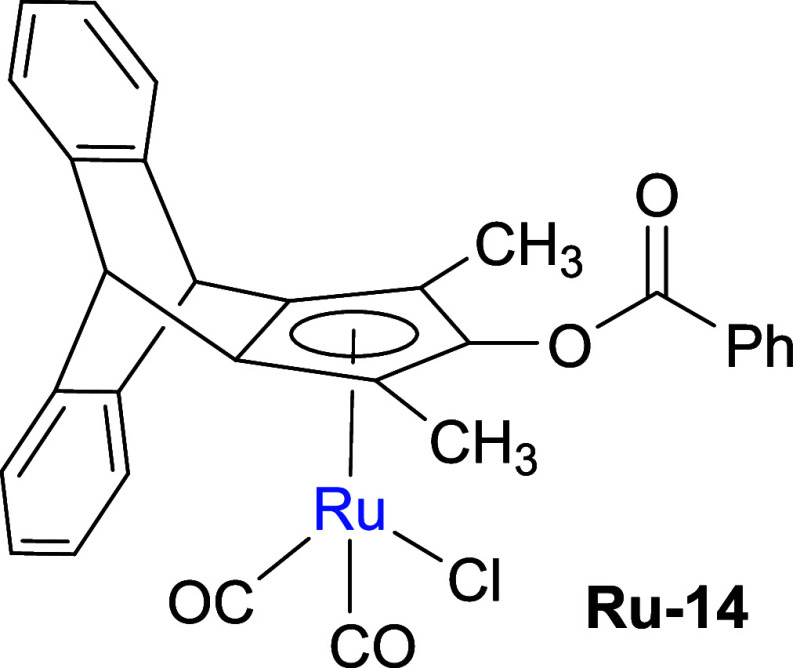


#### Synthesis of Iron Complex **Fe-15**

A 10 mL microwave vial was charged with compound **8** (70.0 mg, 0.24 mmol, 1.0 equiv) and iron pentacarbonyl (0.086 mL, 0.64 mmol, 2.6 equiv) in toluene (3 mL). The reaction mixture was heated to 140 °C for 16 h. The volatiles were removed under reduced pressure, and the crude product was purified by column chromatography on silica gel (gradient: *n*-heptane/EtOAc = 30 → 50%) to afford 90 mg (92%) of **Fe-15** as a yellow solid. Mp = 202–204 °C (decomp).

^1^H NMR (300 MHz, CDCl_3_): δ = 7.37 (dd, *J* = 5.3, 3.3, 4H), 7.13 (dd, *J* = 5.4, 3.1, 2H), 7.05 (dd, *J* = 5.3, 3.1, 2H), 5.19 (s, 2H), 1.89 (s, 6H). ^13^C­{^1^H} NMR (75 MHz, CDCl_3_): δ = 208.30, 171.70, 144.22, 143.75, 126.56, 126.35, 124.68, 123.27, 109.90, 45.71, 9.09. IR (neat, cm^–1^): 3067, 2958, 2919, 2854, 2050, 1996, 1979, 1627, 1451, 1379, 1037, 777, 759, 704, 596, 575. HRMS (ESI) calcd for C_24_H_16_FeO_4_ (M + H)^+^: 425.0471, found: 425.0472.
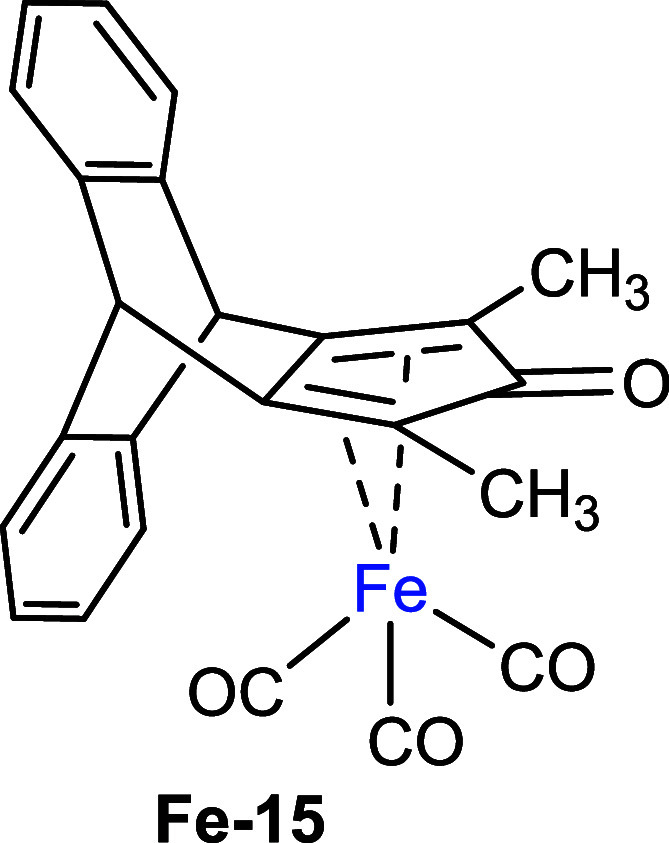


#### Synthesis of Cobalt Complex **Co-16**

A flame-dried Schlenk tube was charged with Co_2_(CO)_8_ (99.1 mg, 0.29 mmol, 1.10 equiv) under nitrogen. A solution of compound **9** (70.0 mg, 0.26 mmol, 1.15 equiv) in dry DCM (3 mL) was added. The black reaction mixture was stirred at 40 °C for 16 h. The flask was connected to a Schlenk line and all volatiles removed in vacuo. A solution of iodine (78.9 mg, 0.31 mmol, 1.07 equiv) in distilled Et_2_O (3 mL) was added to the black residue and stirred for 12 h at room temperature. Since small amounts of CO are released during this reaction, the overpressure needs to be relieved via the Schlenk line, e.g., via connection to a bubbler. Afterwards, all volatiles were evaporated in vacuo, and the residue was purified by column chromatography on silica gel (gradient: *n*-heptane/DCM = 0 → 60%). The dark violet fractions were collected and concentrated under reduced pressure to obtain 69.0 mg (44%) of the desired product **Co-16** as a black solid. Mp > 370 °C.

^1^H NMR (300 MHz, CDCl_3_): δ = 7.52 (dd, *J* = 5.4, 3.2, 2H), 7.37–7.28 (m, 2H), 7.17 (dd, *J* = 5.5, 3.2, 2H), 7.03 (dd, *J* = 5.4, 3.2, 2H), 5.44 (s, 1H), 5.11 (s, 2H), 2.48 (s, 6H). ^13^C­{^1^H} NMR (75 MHz, CDCl_3_): δ = 143.53, 143.37, 126.90, 126.42, 124.82, 124.78, 115.27, 95.02, 94.02, 45.55, 12.69. IR (neat, cm^–1^): 3062, 3041, 2972, 2913, 2038, 1484, 1451, 1396, 1369, 1215, 1174, 1148, 1018, 889, 785, 732, 636, 568, 518. HRMS (ESI) calcd for C_21_H_14_ CoI_2_ [(M – Co) + NH_4_]^+^: 599.9090, found: 599.9093.
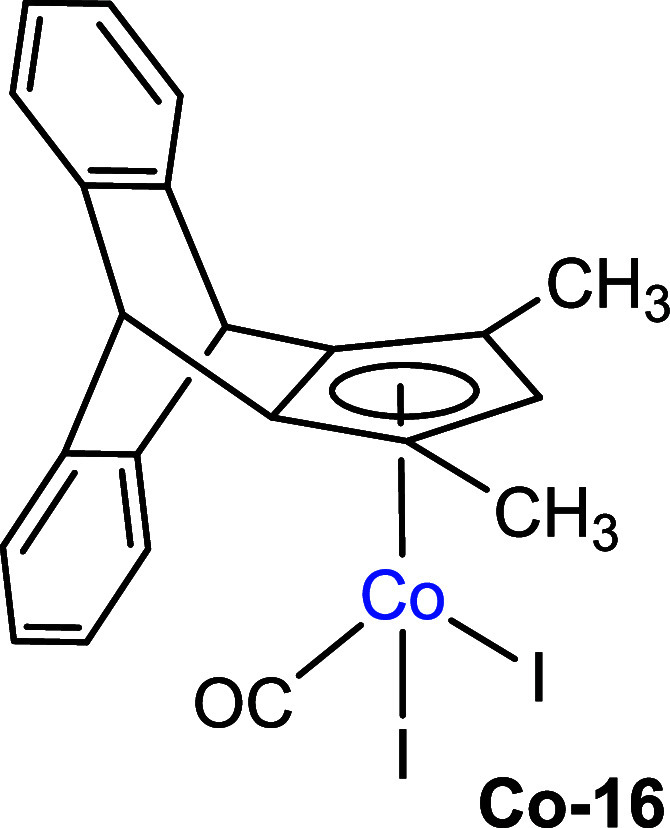


### Reactivity Screening of Precatalyst **Ru-14** with Triynes in Cyclotrimerization Reactions

#### Thermal Conditions

A 10 mL microwave vial was charged with substrate triyne **17a**–**c** (50 mg, 1.0 equiv) and **Ru-14** (5 mol %) in 2 mL of toluene. The reaction mixture was heated to 120 °C for 1 h. The volatiles were removed under reduced pressure, and the crude product was purified by column chromatography on silica gel (gradient: *n*-heptane/EtOAc = 0 → 20%) to afford the desired product **18a**–**c** as a solid.

All compounds’ analytical data agree with the values stated in the literature.^36^

##### Compound **18a** (48.0 mg, 96% Yield)

^1^H NMR (300 MHz, CDCl_3_) δ = 7.48 (d, *J* = 8.1, 4H), 7.14 (d, *J* = 8.0, 4H), 5.16 (t, *J* = 1.7, 4H), 4.96 (t, *J* = 1.7, 4H). ^19^F NMR (282 MHz, CDCl_3_) δ = −62.6.

##### Compound **18b** (41.0 mg, 82% Yield)

^1^H NMR (300 MHz, CDCl_3_) δ = 7.15 (s, 2H), 5.13 (t, *J* = 1.6, 4H), 5.03 (t, *J* = 1.7, 4H). ^13^C­{^1^H} NMR (75 MHz, CDCl_3_) δ: 138.83, 132.48, 120.02, 73.56, 72.33.

##### Compound **18c** (46.0 mg, 93% Yield)

^1^H NMR (300 MHz, CDCl_3_) δ = 7.03–6.85 (m, 8H), 5.14 (t, *J* = 1.7, 4H), 4.96 (t, *J* = 1.6, 4H). ^19^F NMR (282 MHz, CDCl_3_) δ = −114.8 (tt, *J* = 8.6, 5.5).

#### Photochemical Conditions

A magnetic stirring bar vial was charged with substrate triyne **17a**–**c** (25 mg, 1.0 equiv) and **Ru-14** (5 mol %.) in 1 mL of toluene. The reaction mixture was stirred under light irradiation at 365 nm for 24 h at room temperature. The volatiles were removed under reduced pressure, and the crude product was purified by column chromatography on silica gel (gradient: *n*-heptane/EtOAc = 0 → 20%) to afford the desired product **18a** (22.0 mg, 88%), **18b** (23.4 mg, 94%) and **18c** (21.5 mg, 86%) as solids. The analytical data are consistent with the values reported for compounds **18a**, **18b**, and **18b** in previous examples.

### Reactivity Screening for Precatalyst **Fe-15** in Acetophenones Transfer Hydrogenation Reactions

Four glass vials were charged with the substrate ketones **19a**–**d** (50 mg, 1.0 equiv), **Fe-15** (2 mol %) and anhydrous Me_3_NO (4 mol %) in 1 mL of dry 2-propanol. All vials were placed into an alloy plate equipped with a septum and an inlet needle. The alloy plate with vials was then placed in an autoclave and sealed. At room temperature, the autoclave was purged 4 times with hydrogen and finally pressurized to 30 bar with hydrogen. The autoclave was then heated to 80 °C for 24 h. The reaction was allowed to cool to rt, and excessive hydrogen was removed. After discharging the H_2_, four drops of each sample were dissolved in MeOH (1 mL) and filtered through a syringe paper filter for GC-MS analysis. All of the samples showed quantitative conversions of the starting materials. For two examples, the volatiles were removed under reduced pressure, and the crude product was purified by column chromatography on silica gel (gradient: *n*-heptane/EtOAc = 5 → 10%) to afford the desired product.

All compounds’ analytical data agree with the values stated in the literature.^29a^

#### Compound **20a** (White Solid 50.0 mg, 98% Yield)

^1^H NMR (300 MHz, CDCl_3_) δ = 7.89–7.77 (m, 4H), 7.55–7.44 (m, 3H), 5.06 (q, *J* = 6.5, 1H), 2.09 (s, 1H), 1.58 (d, *J* = 6.5, 3H).

#### Compound **20c** (Liquid 45.8 mg, 91% Yield)

^1^H NMR (300 MHz, CDCl_3_) δ = 7.35–7.24 (m, 2H), 6.93–6.83 (m, 2H), 4.85 (q, *J* = 6.4, 1H), 3.80 (s, 3H), 1.91 (s, 1H), 1.47 (d, *J* = 6.4, 3H).

### Reactivity Screening of Precatalyst **Fe-15** with Triynes in Cyclotrimerization Reactions

A 10 mL microwave vial was charged with substrate triyne **17a**–**b** (25.0 mg) and **Fe-15** (5 mol %) in 1 mL of toluene. The reaction mixture was heated to 120 °C for 24 h. The volatiles were removed under reduced pressure, and the crude product was purified by column chromatography on silica gel (gradient: *n*-heptane/EtOAc = 0 → 20%) to afford the desired products **18a** (18.0 mg, 72% yield), **18b** (16.0 mg, 64% yield) as solids. The analytical data are consistent with the values reported for compounds **18a** and **18b** in previous examples.

### Reactivity Screening for Precatalyst **Co-16** in C–H Functionalization Reactions of *N*-Chlorobenzamides and Styrenes

A 10 mL microwave vial was charged with *N*-chlorobenzamide **21** (25.0 mg, 0.16 mmol, 1.0 equiv), cobalt catalyst **Co-16** (1.96 mg, 3.21 μmol, 2 mol %), potassium pivalate (27.0 mg, 0.19 mmol, 1.2 equiv) as well as silver triflate (1.65 mg, 6.42 μmol, 4.0 mol %) in HFIP solvent (500 μL) under argon. The reaction mixture was stirred for 5 min, and then the respective styrene **22a**–**c** (1.5 equiv) was added. The resulting brown mixture was stirred for 36 h at 40 °C. The reaction was then quenched by adding AcOH (50 μL) and EtOAc (500 μL), stirred for 15 min at rt, and filtered through a pad of silica gel in vacuo. The crude mixture was purified by column chromatography on silica gel (eluent *n*-heptane/EtOAc 5 → 30%) to afford the desired product **23a**–**c** as a solid.

All compounds’ analytical data agree with the values stated in the literature.^31a^

#### Compound **23a** (28.0 mg, 78% Yield)

^1^H NMR (300 MHz, CDCl_3_); δ = 8.13 (dd, *J* = 7.6, 1.5, 1H), 7.53–7.28 (m, 7H), 7.19 (d, *J* = 7.4, 1H), 6.02 (s, 1H), 4.86 (ddd, *J* = 10.8, 5.1, 1.2, 1H), 3.29–3.06 (m, 2H).

#### Compound **23b** (30.0 mg, 75% Yield)

^1^H NMR (300 MHz, CDCl_3_) δ = 8.12 (dd, *J* = 7.6, 1.5, 1H), 7.46 (td, *J* = 7.4, 1.6, 1H), 7.39 (dt, *J* = 7.5, 1.1, 1H), 7.36–7.29 (m, 2H), 7.22–7.15 (m, 1H), 6.97–6.86 (m, 2H), 5.95 (s, 1H), 4.86–4.75 (m, 1H), 3.82 (s, 3H), 3.27–3.00 (m, 2H).

#### Compound **23c** (33.0 mg, 86% Yield)

^1^H NMR (300 MHz, CDCl_3_): δ = 8.10 (dd, *J* = 7.7, 1.6, 1H), 7.47 (ddd, *J* = 8.8, 7.0, 1.4, 1H), 7.38 (ddd, *J* = 8.6, 4.9, 2.2, 3H), 7.18 (d, *J* = 7.4, 1H), 7.12–7.02 (m, 2H), 6.19 (s, 1H), 4.90–4.80 (m, 1H), 3.23–3.06 (m, 2H). ^19^F NMR (282 MHz, CDCl_3_): δ −113.5.

## Supplementary Material



## Notes

Detailed experimental information on the synthesis of starting materials and intermediate products; spectral data for all synthesized compounds (PDF).

## Notes

Dedicated to Prof. Dr. Uwe Rosenthal on the occasion of his 75th birthday and in appreciation of his important contributions to Cp-transition metal chemistry.
