# Supplementary material for: Triptycepenes: Synthesis, Metal Complexes, and Their Reactivity in Catalytic Reactions
Source: Organometallics. 2025 May 29;44(11):1200–9. doi: 10.1021/acs.organomet.5c00102 (PMC12152958; doi:10.1021/acs.organomet.5c00102)
Supplement: Supplementary file 1 [file om5c00102_si_001.pdf]

Supporting Information

Triptycepenes: Synthesis, Metal Complexes and Their Reactivity in Catalytic Reactions

Shyam Sundar Mothuku,*[§,Δ] Uwe Monkowius [Δ] and Marko Hapke*[§,Δ,#]

[§] Institute for Catalysis (INCA), Johannes Kepler University Linz (JKU), Altenberger Strasse 69, A-4040 Linz (Austria)

[Δ] Institute of Inorganic Chemistry (IAC), JKU Linz, Altenberger Strasse 69, A-4040 Linz (Austria)

[#] Leibniz Institute for Catalysis e.V. (LIKAT), Albert-Einstein-Strasse 29a, D-18059 Rostock (Germany)

Shyam Sundar Mothuku: ORCID: https://orcid.org/0000-0003-3732-2647; Researcher-ID: AAC-6068-2021; E-mail: shyam_sundar.mothuku@jku.at
Uwe Monkowius: ORCID: https://orcid.org/0000-0003-0662-2866
Marko Hapke: ORCID: https://orcid.org/0000-0001-7454-9184; Researcher-ID: I-7444-2012; E-mail: marko.hapke@jku.at

Table of Contents

1. Synthesis of Compounds ____ SI-2
2. SC-XRD Data ____ SI-6
3. NMR Spectra ____ SI-10
4. References ____ SI-43

1. Synthesis of Compounds

Compound 3[1]

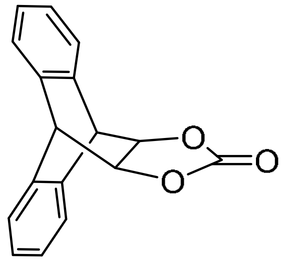

Anthracene (**1**, 3.00 g, 16.8 mmol, 1.0 equiv.), vinylene carbonate (**2**, 2.32 g, 1.71 mL, 26.9 mmol, 1.6 equiv.), and anhydrous toluene (30 mL) were placed in a pressure tube under nitrogen, and the vessel was sealed tightly. The mixture was stirred at 180 °C for 3 days. The reaction mixture was moved to a 100 mL round-bottomed flask and concentrated under vacuo to furnish a white solid. The solid was repeatedly washed with methanol and then dried under vacuo to obtain a white compound in 3.61 g (81%) yield. The product was spectroscopically pure **3** based on the ^{1}H NMR spectrum and was used directly for the next step without purification.

^{1}H NMR (300 MHz, $CDCl_3$): δ = 7.46-7.37 (m, 4H), 7.30-7.25 (m, 4H), 4.91 (s, 2H), 4.73 (s, 2H) ppm. **^{13}C NMR** (75 MHz, $CDCl_3$): δ = 154.21, 137.82, 136.38, 127.92, 127.79, 126.72, 125.77, 76.37, 47.84 ppm. **HRMS** (ESI) calcd for $C_{17}H_{12}O_3$ $(M+H)^+$: 265.0859, found: 26.0860.

Compound 4[1]

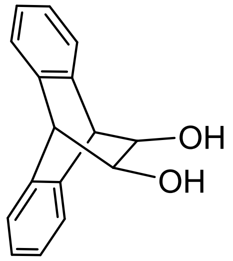

In a 500 mL two-necked flask containing a stirring bar, anthracene carbonate **3** (3.50 g, 13.3 mmol), 1,4-dioxane (90 mL), and 4N NaOH (30 mL) were placed under nitrogen. The mixture was stirred at reflux for 2 h. The reaction mixture was neutralized with 1N HCl (ca. 25 mL). The aqueous layer was then separated from the organic layer and extracted with CH_2Cl_2 (2x 30 mL), and the combined organic layers were washed with water (3x 30 mL) and dried over Na_2SO_4. After filtering, the filtrate was concentrated in vacuo to give a white solid. The solid was washed with 10x 20 mL of hexane and dried under vacuo to yield **4** as a white solid (2.92 g, 93%). The compound was shown to be spectroscopically pure in ^{1}H NMR analysis and was used directly for the next step without further purification.

^{1}H NMR (300 MHz, $CDCl_3$) δ = 7.40 (dd, *J* = 5.4, 3.2 Hz, 2H), 7.34 (dd, *J* = 5.4, 3.2 Hz, 2H), 7.23 (dd, *J* = 5.4, 3.2 Hz, 2H), 7.18 (dd, *J* = 5.4, 3.2 Hz, 2H), 4.44 (t, *J* = 1.6 Hz, 2H), 4.07 (t, *J*

= 1.6 Hz, 2H), 2.25 (s, 2H) ppm. **^{13}C NMR** (75 MHz, $CDCl_3$) δ = 140.04, 138.74, 126.86, 126.80, 126.64, 124.88, 68.24, 51.44 ppm. **HRMS** (ESI) calcd for $C_{16}H_{14}O_2$ $(M+Na)^+$: 261.0886, found: 261.0883.

Compound 5[1]

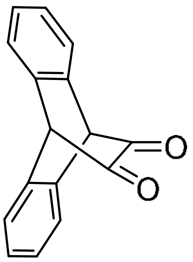

In a 500 mL three-necked flask equipped with a 50 mL dropping funnel were placed dry CH_2Cl_2 (80 mL) and anhydrous DMSO (2.19 g, 2.0 mL, 28.1 mmol, 2.7 equiv.), and the mixture was cooled to -78 °C. Trifluoroacetic anhydride (5.50 g, 3.66 mL, 26.2 mmol, 2.5 equiv.) was added dropwise to the mixture at -78 °C over a period of 10 min, and the resulting mixture was stirred at the same temperature for 30 min. Subsequently, a solution of α-diol **4** (2.50 g, 10.5 mmol) in DMSO (20 mL) and CH_2Cl_2 (40 mL) was added dropwise to the mixture at -78 °C over a period of 30 min, and the resulting mixture was then stirred at the same temperature for an additional 30 min. Finally, triethylamine (6.0 g, 8.23 mL, 59.5 mmol) was added dropwise at -78 °C over a period of 15 min, and the resulting mixture was then stirred at the same temperature for 1 h. The solution was warmed to room temperature. ***From this point on, all subsequent operations were performed under light shielding as much as possible to prevent the decomposition of the product by ambient light.*** Aqueous 1N HCl (250 mL) was added to the reaction mixture, and the aqueous layer was separated from the organic layer and extracted with CH_2Cl_2 (6x 20 mL). The combined organic layers were washed with water (3x 15 mL) and brine (2x 20 mL) and dried over Na_2SO_4. After filtrating, the filtrate was concentrated in vacuo to give a pale-yellow solid. The solid was crystallized from CH_2Cl_2/*n*-hexane to give anthracene α-diketone **5** in 77% yield (1.89 g) as a pale-yellow solid.
^{1}H NMR (300 MHz, $CDCl_3$): δ = 7.53-7.43 (m, 4H), 7.42-7.34 (m, 4H), 5.00 (s, 2H) ppm. **^{13}C NMR** (75 MHz, $CDCl_3$): δ = 183.88, 134.96, 129.53, 126.45, 60.10 ppm. **HRMS** (ESI) calcd for $C_{16}H_{10}O_2$ $(M+NH_4)^+$: 252.1019, found: 252.1017.

Synthesis of compound 8

The elimination of water in acidic conditions from compound **7** produced two distinct products: the non-polar *endo*-formation of **8** (*endo*-**8**) and the polar *exo*-formation **8** (*exo*-**8**, Scheme S-1). We observed that under cold conditions, the reaction concentration and reaction time, as well as the rate of addition of H_2SO_4 (very slow addition) favor the formation of the *endo* product as the major product. The ^{1}H NMR spectrum of *endo*-**8** shows four signals (see the spectra

section), indicating the molecules' symmetrical nature. We observed that the methyl proton appeared as a singlet at 1.72 ppm, while the tertiary CH proton also showed as a singlet at 5.16 ppm. The eight aromatic protons appeared as two sets of doublets at 7.22 and 7.36 ppm. In contrast, the ^{1}H NMR spectrum of the polar product exhibited a mixture of signals of *exo*, keto form and its *enol form* tautomerism. The *exo* form showed characteristic doublet and quartet signals, along with hydroxy proton signals indicative of *keto*-*enol* formation.

<u>Compound *exo*-**8**</u>

^{1}H NMR (300 MHz, $CDCl_3$) δ = 7.47-7.38 (m, 2H), 7.34-7.24 (m, 2H), 7.25-7.06 (m, 8H), 5.64 (s, 1H), 5.50 (s, 1H), 5.34 (d, *J* = 3.6, 2H), 2.96 (q, *J* = 7.7, 1H), 2.08 (q, *J* = 7.2, 1H), 1.81 (d, *J* = 9.2, 5H), 1.51 (d, *J* = 7.5, 5H), 1.23 (d, *J* = 7.1, 2H), 0.79 (d, *J* = 7.8, 3H).

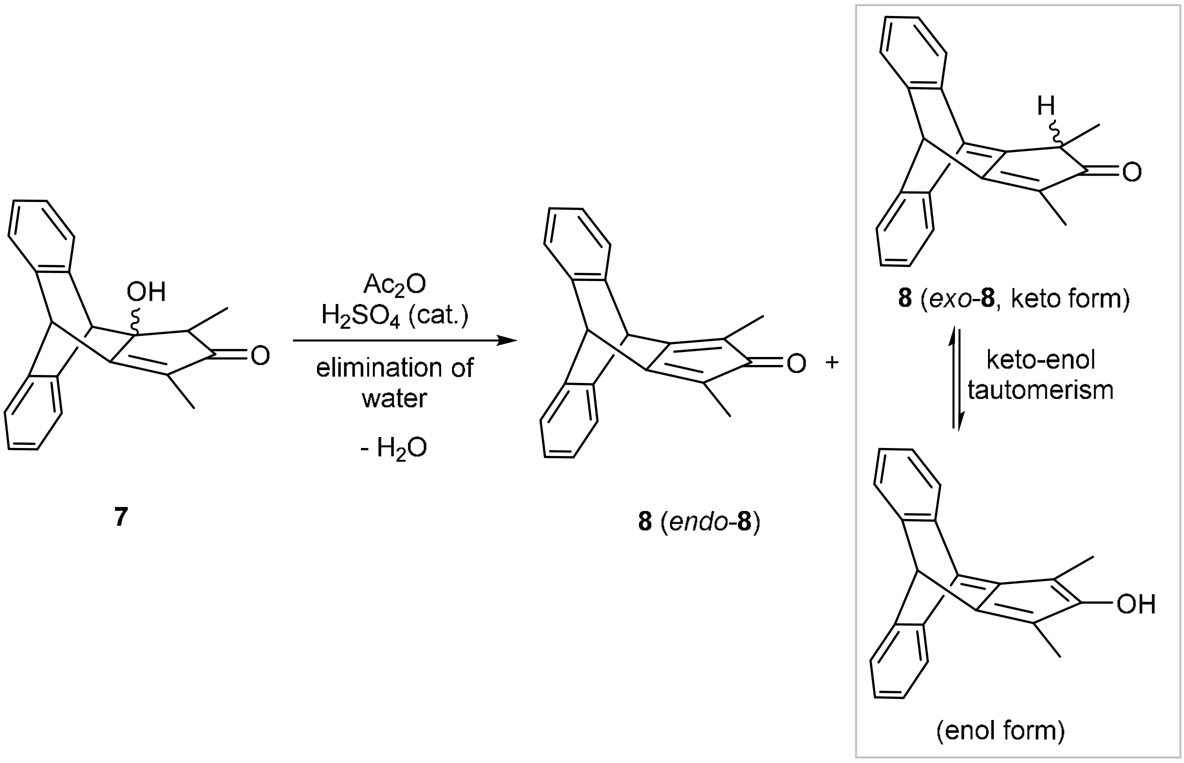

Scheme S-1. Elimination of water from compound **7**.

Synthesis of compounds 9 and 13

The reduction of tetra-substituted cyclopentadienone using $AlCl_3$-$LiAlH_4$ with varying equivalent ratios has been reported.[2] Following those procedures, the reduction of cyclopentadienone **8** or **12** reactions was carried out using different ratios of reagents (Scheme S-2). We observed that the reduction of compound **8** in the presence of 16 equivalent of $LiAlH_4$ predominantly the reduced product **9** was yielded, along with a hydroxy byproduct **9-OH**. In contrast, the reduction of compound **12** resulted in the exclusive isolation of the reduced product **13**. In the case of using 2.5 equivalent of $LiAlH_4$, predominantly the hydroxy product **9-OH** or **13-OH** were obtained. Increasing the equivalents of hydride donor shifts the product

ration **9**/**9-OH** towards the cyclopentadiene. However, only a large excess of $LiAlH_4$ finally pushes the reaction towards formation of the desired free cyclopentadienes.

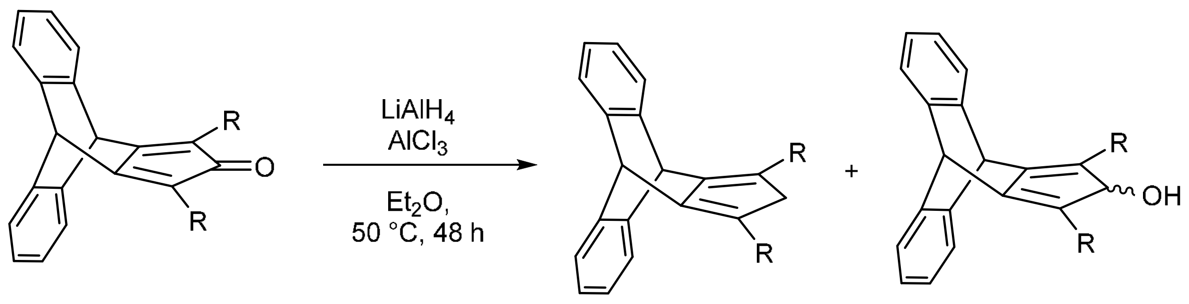

**8** (R = CH_3)<br>**12** (R = Ph)	i) $LiAlH_4$ (16 equiv.)<br>$AlCl_3$ (4.0 equiv.)	**9** (R = CH_3, 71%)<br>**13** (R = Ph, 91%)	**9-OH** (R = CH_3, 11%)
	ii) $LiAlH_4$ (2.5 equiv.)<br>$AlCl_3$ (2.0 equiv.)	**9** (R = CH_3, 6%)<br>**13** (R = Ph, 4%)	**9-OH** (R = CH_3, 75%)<br>**13-OH** (R = Ph, 88%)
	iii) $LiAlH_4$ (5.0 equiv.)<br>$AlCl_3$ (4.0 equiv.)	**9** (R = CH_3, 21%)	**9-OH** (R = CH_3, 63%)
	iv) $LiAlH_4$ (10.0 equiv.)<br>$AlCl_3$ (4.0 equiv.)	**9** (R = CH_3, 45%)	**9-OH** (R = CH_3, 36%)

Scheme S-2. Reduction of cyclopentadienone.

Compound **9-OH**

The structure was confirmed by single crystal X-ray analysis. See page no. SI-7, Figure S2). ^{1}H NMR (300 MHz, $CDCl_3$) δ = 7.33-7.28 (m, 4H), 7.12-7.06 (m, 4H), 4.93 (s, 2H), 4.35 (s, 1H), 1.90 (s, 6H). **HRMS** (ESI) calcd for $C_{21}H_{19}O$ $(M+H)^+$: 287.1430, found: 287.1433.

Compound **13-OH**

^{1}H NMR (300 MHz, $CDCl_3$) δ = 7.50 (ddd, *J* = 8.4, 4.2, 2.4, 6H), 7.38 (dd, *J* = 8.4, 6.9, 4H), 7.28-7.11 (m, 6H), 6.97 (dd, *J* = 5.4, 3.2, 2H), 5.69 (s, 1H), 5.44 (s, 2H). **^{13}C NMR** (75 MHz, $CDCl_3$) δ 142.40, 142.29, 142.10, 134.43, 133.58, 128.94, 128.09, 126.99, 126.86, 126.46, 124.37, 83.41, 47.34. **HRMS** (ESI) calcd for $C_{31}H_{23}O$ $(M+H)^+$: 411.1743, found: 411.1744.

2. SC-XRD Data

X-ray quality crystals were grown via slow evaporation from a concentrated solution of **9** in n-hexane at ambient temperature, compound **Ru-14** in DCM layered with hexane at -40 °C, compound **Fe-15** in DCM layered with hexane at ambient temperature and compound **Co-16** in DCM layered with hexane at -40 °C. Single-crystal structure analysis was carried out at room temperature on a Bruker D8 Quest ECO diffractometer with graphite-monochromated MoKα radiation (λ = 0.71073 Å). The structures were solved by direct methods (SHELXS-2013/1[3]) and refined by full-matrix least-squares on F^2 (SHELXL-2018/3[4]). The H atoms were calculated geometrically, and a riding model was applied in the refinement process. Crystallographic details for **9** (CCDC 2423988) and compound **9-OH** (2423992) can be found in Table S-1, compounds **Ru-14** (CCDC 2423987), **Fe-15** (CCDC 2423990) and **Co-16** (CCDC 2423991) can be found in Table S-2. CCDC contains the supplementary crystallographic data information can be obtained free of charge via https://www.ccdc.cam.ac.uk/structures/

Figure S-1: Molecular structure of **9** (thermal ellipsoids drawn at the 50% probability level at 150 K).

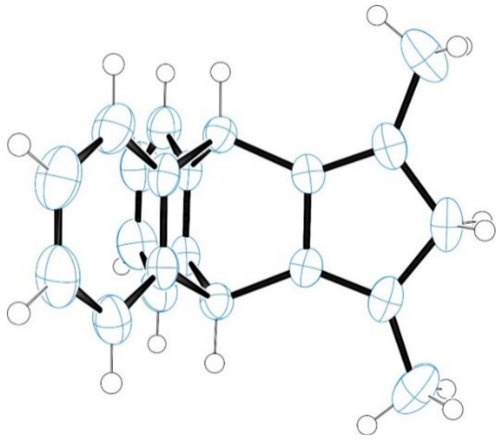

Figure S-2: Molecular structure of **9-OH** (thermal ellipsoids drawn at the 50% probability level at 150 K).

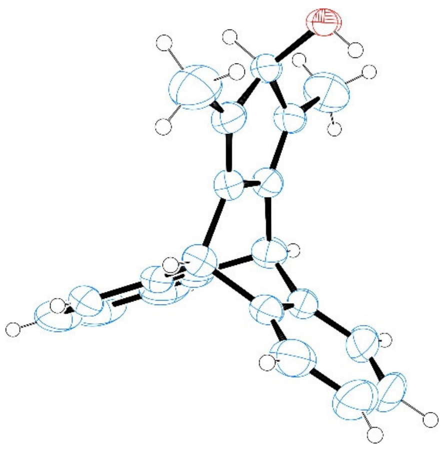

Figure S-3: Molecular structure of **Ru-14** (thermal ellipsoids drawn at the 50% probability level at 150 K, hydrogen atoms omitted for clarity).

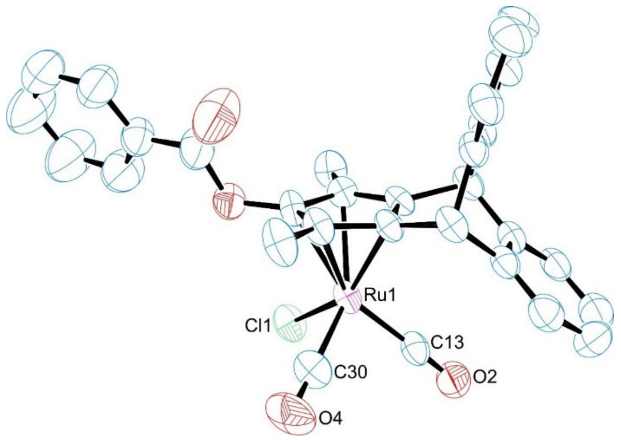

Figure S-4: Molecular structure of **Fe-15** (thermal ellipsoids drawn at the 50% probability level at 150 K, hydrogen atoms omitted for clarity).

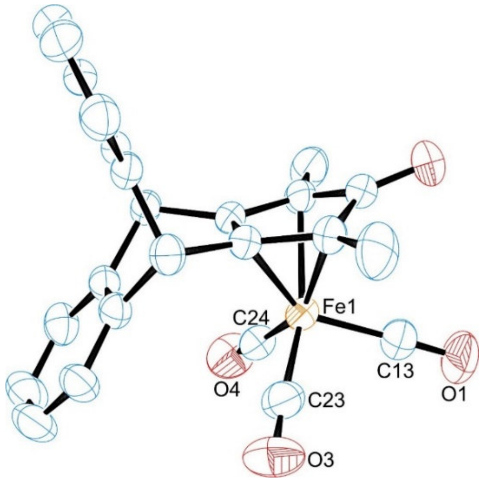

Figure S-5: Molecular structure of **Co-16** (thermal ellipsoids drawn at the 50% probability level at 150 K, hydrogen atoms omitted for clarity).

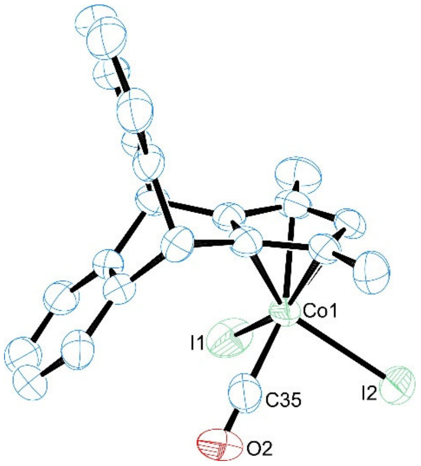

Table S-1: Crystal data, data collection and structure refinement details for compound **9** and **9-OH**.

Compound	**9**	**9**-OH
Empirical formula	$C_{21}H_{18}$	$C_{21}H_{18}O$ [+solvent]
Formula weight	270.35	286.35
Crystal system	monoclinic	trigonal
Space group	*C*2/c	$R\bar{3}c$
Temp/K	296	296
a (Å)	14.226(2)	20.9672(3)
b (Å)	7.8604(12)	-
c (Å)	13.905(2)	42.2604(9)
α (°)	-	-
β (°)	106.018(7)	-
γ (°)	-	-
V (Å^3)	1494.5(4)	16089.6(6)
Z	4	36
D_{calc} (g/cm^3)	1.201	1.064
μ / mm^{-1}	0.07	0.06
Reflns collected	40497	270797
Indep. reflns	1519	3663
Obs. reflns [I > 2σ(I)]	1285	2417
Param. refin./restr.	97	192
Absorption correction	multi-scan	multi-scan
Final R_1 [$I > 2\ \sigma(I)$]	0.0454	0.0705
Final $wR_2(F^2)$ [$I > 2\ \sigma(I)$]	0.1231	0.1952
Final R_1 (all data)	0.0531	0.0986
Final $wR_2(F^2)$ (all data)	0.1295	0.2143
$\Delta\rho_{(max/min)}$ / e Å^{-3}	0.27/-0.14	0.30/-0.28
CCDC	2423988	2423992

Table S-2: Crystal data, data collection and structure refinement details for compound **Ru-14, Fe-15** and **Co-16**.

Compound	**Ru-14**	**Fe-15**	**Co-16**
Empirical formula	$C_{30}H_{21}ClO_4Ru$	$3(C_{24}H_{16}FeO_4)\cdot 0.5(C_4H_{10})$	$C_{22}H_{17}CoI_2O$
Formula weight	581.99	1301.71	610.08
Crystal system	monoclinic	monoclinic	monoclinic
Space group	$P2_1/n$	$P2_1/c$	$P2_1/c$
Temp/K	296	296	296
a (Å)	12.1243(11)	28.6323(10)	18.8455(8)
b (Å)	17.9917(16)	9.2289(3)	13.9402(6)
c (Å)	13.9485(12)	24.3104(9)	18.0911(7)
α (°)	-	-	-
β (°)	111.506(2)	92.523(1)	118.644(1)
γ (°)	-	-	-
V (Å^3)	2830.8(4)	6417.7(4)	4171.1(3)
Z	4	4	8
D_{calc} (g/cm^3)	1.366	1.347	1.943
μ / mm^{-1}	0.68	0.73	3.79
Reflns collected	5003	147436	137248
Indep. reflns	5003	11286	11202
Obs. reflns [I > 2σ(I)]	3117	6773	6387
Param. refin./restr.	315	799	473
Absorption correction	multi-scan	multi-scan	multi-scan
Final R_1 [$I > 2\ \sigma(I)$]	0.0565	0.0564	0.0498
Final $wR_2(F^2)$ [$I > 2\ \sigma(I)$]	0.1438	0.1523	0.1167
Final R_1 (all data)	0.0992	0.1080	0.1063
Final $wR_2(F^2)$ (all data)	0.1576	0.1719	0.0498
$\Delta\rho_{(max/min)}$ / e Å^{-3}	0.36/-0.93	1.01/-0.64	-1.63/2.05
CCDC	2423987	2423990	2423991

3. NMR Spectra

Figure S-6: ^{1}H NMR ($CDCl_3$, 300 MHz) of **3**

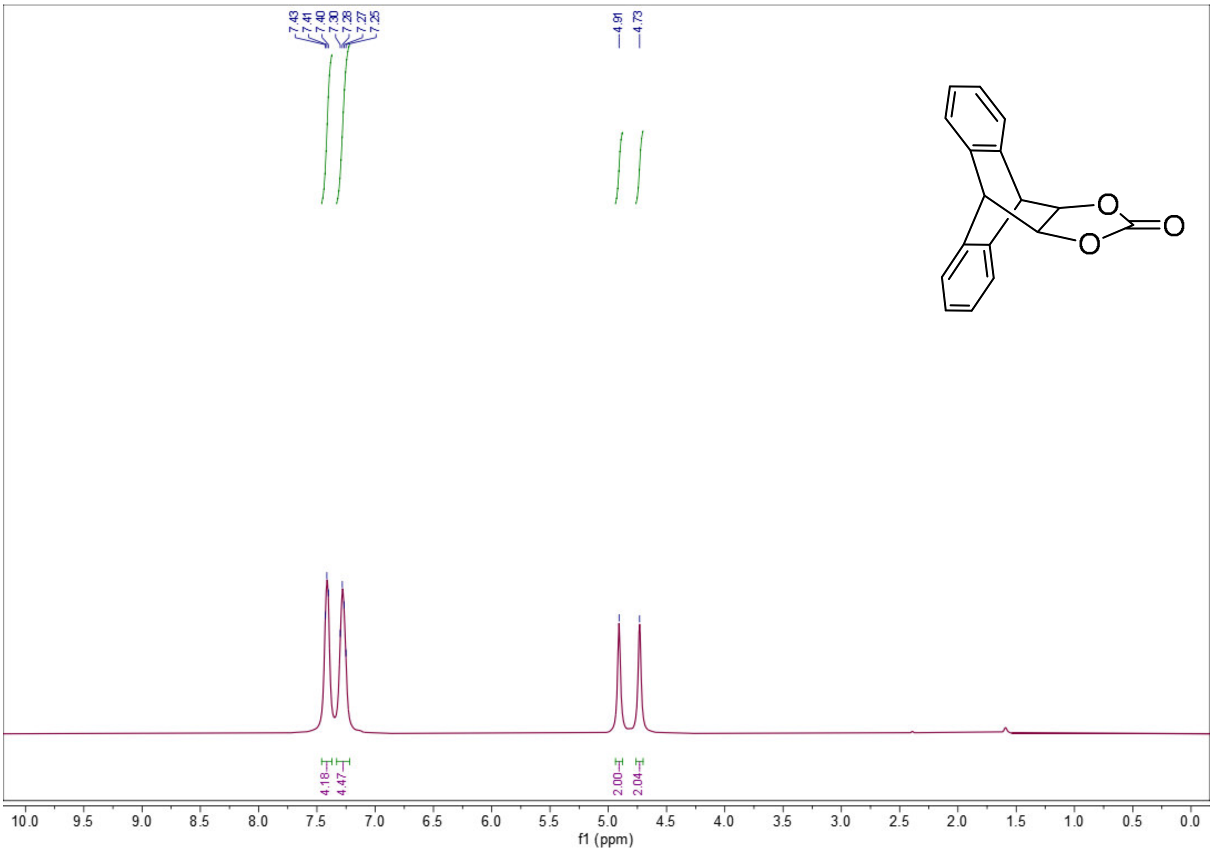

Figure S-7: $^{13}C\{^1H\}$ NMR ($CDCl_3$, 125 MHz) of **3**

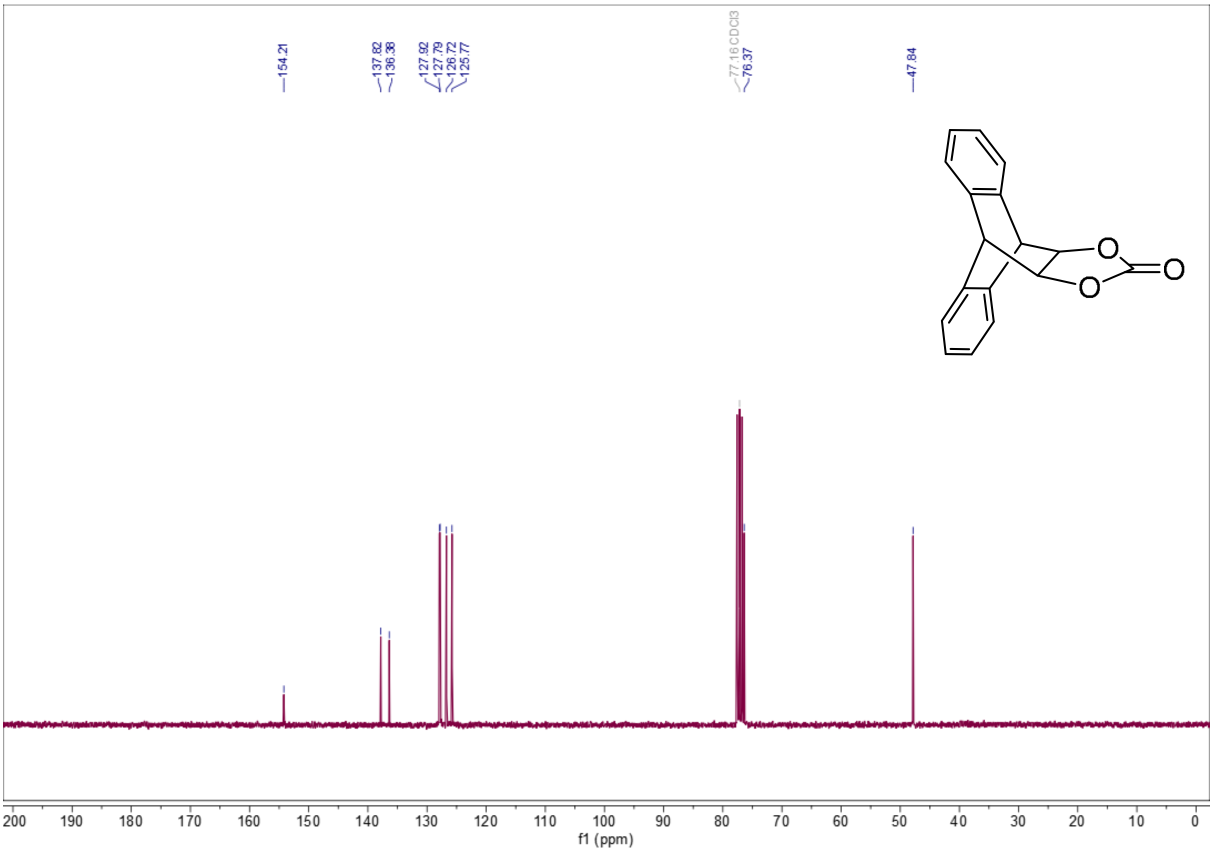

Figure S-8: ^{1}H NMR ($CDCl_3$, 300 MHz) of **4**

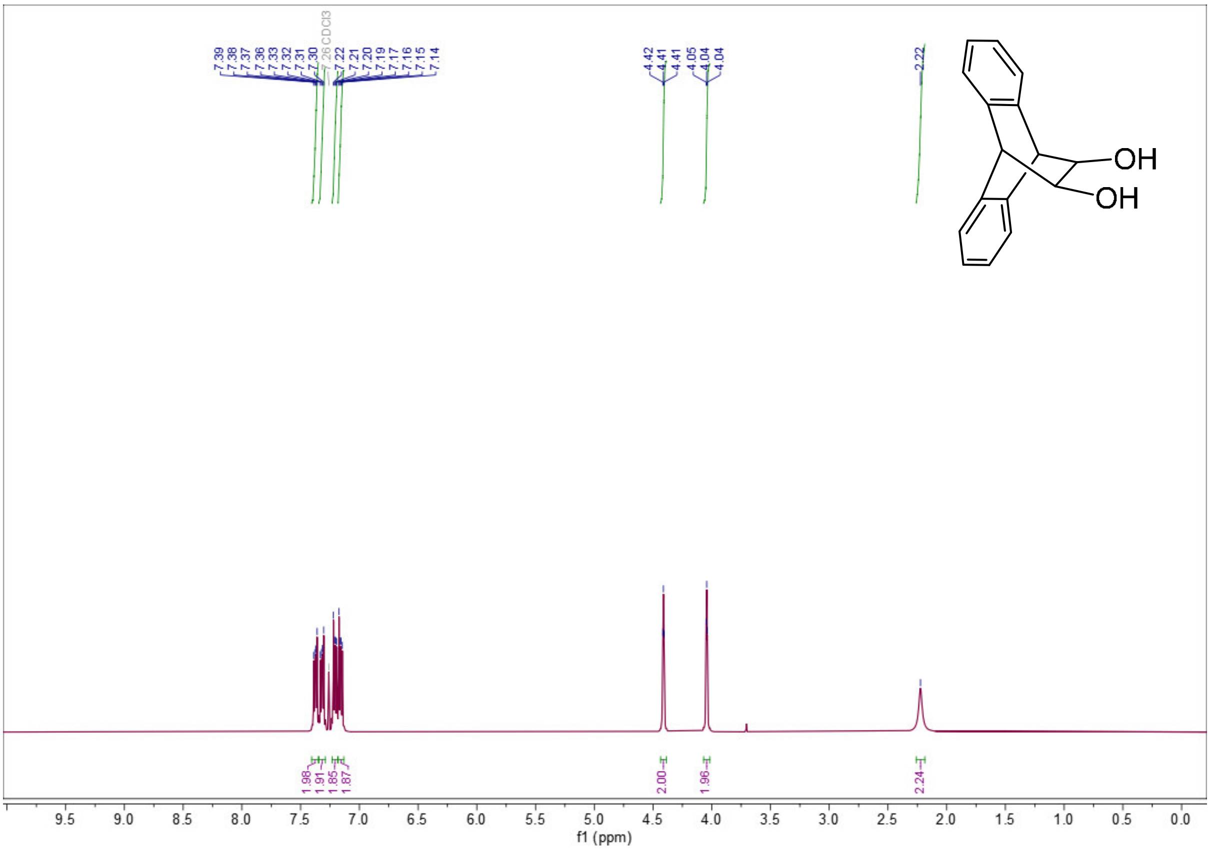

Figure S-9: $^{13}C\{^{1}H\}$ NMR ($CDCl_3$, 125 MHz) of **4**

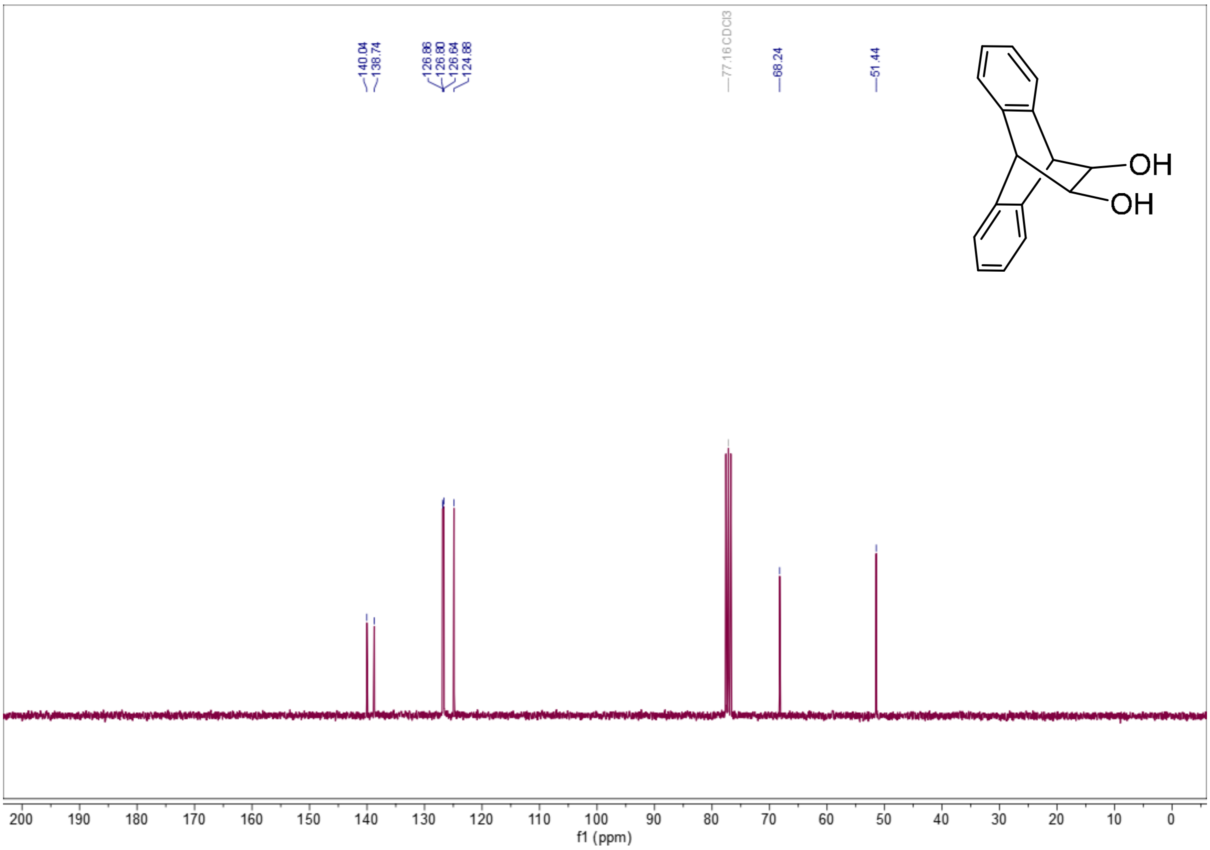

Figure S-10: H,H-COSY ($CDCl_3$, 300 MHz) of **4**

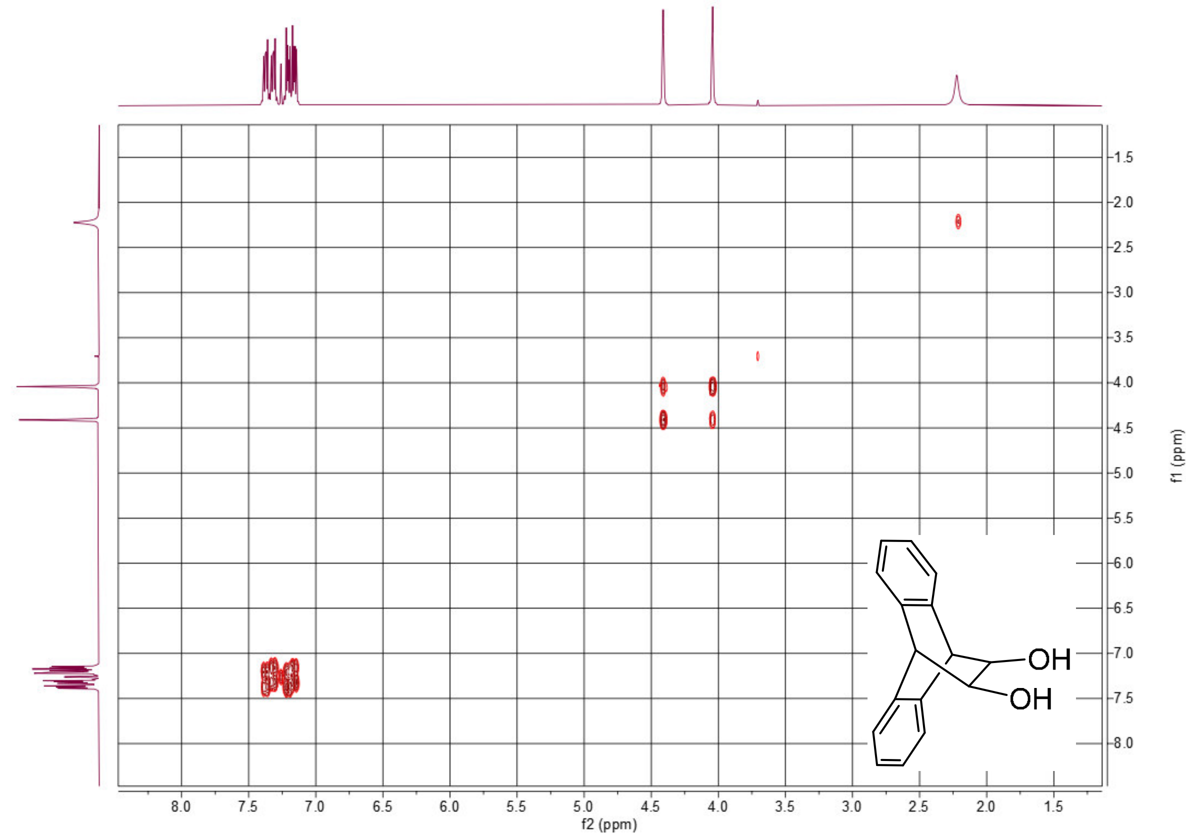

Figure S-11: HSQC ($CDCl_3$, 300 MHz) of **4**

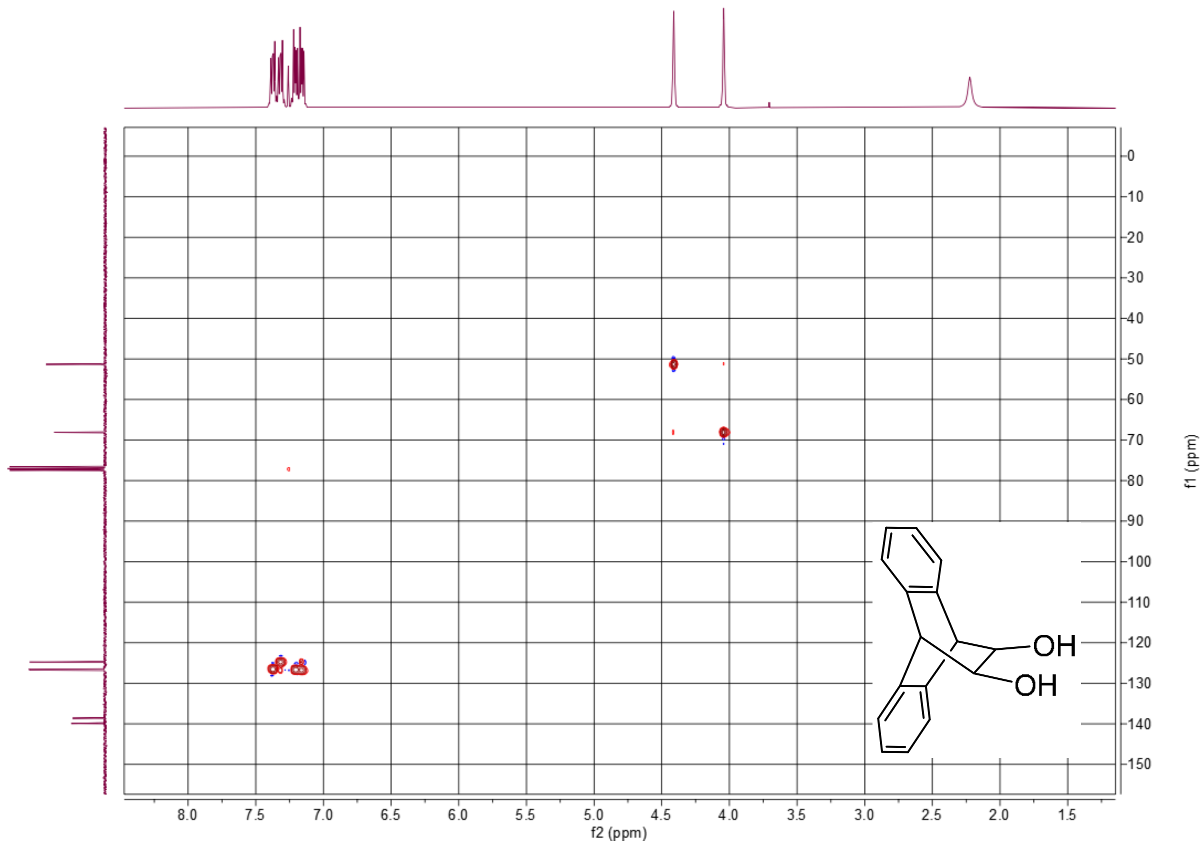

Figure S-12: ^{1}H NMR ($CDCl_3$, 300 MHz) of **5**

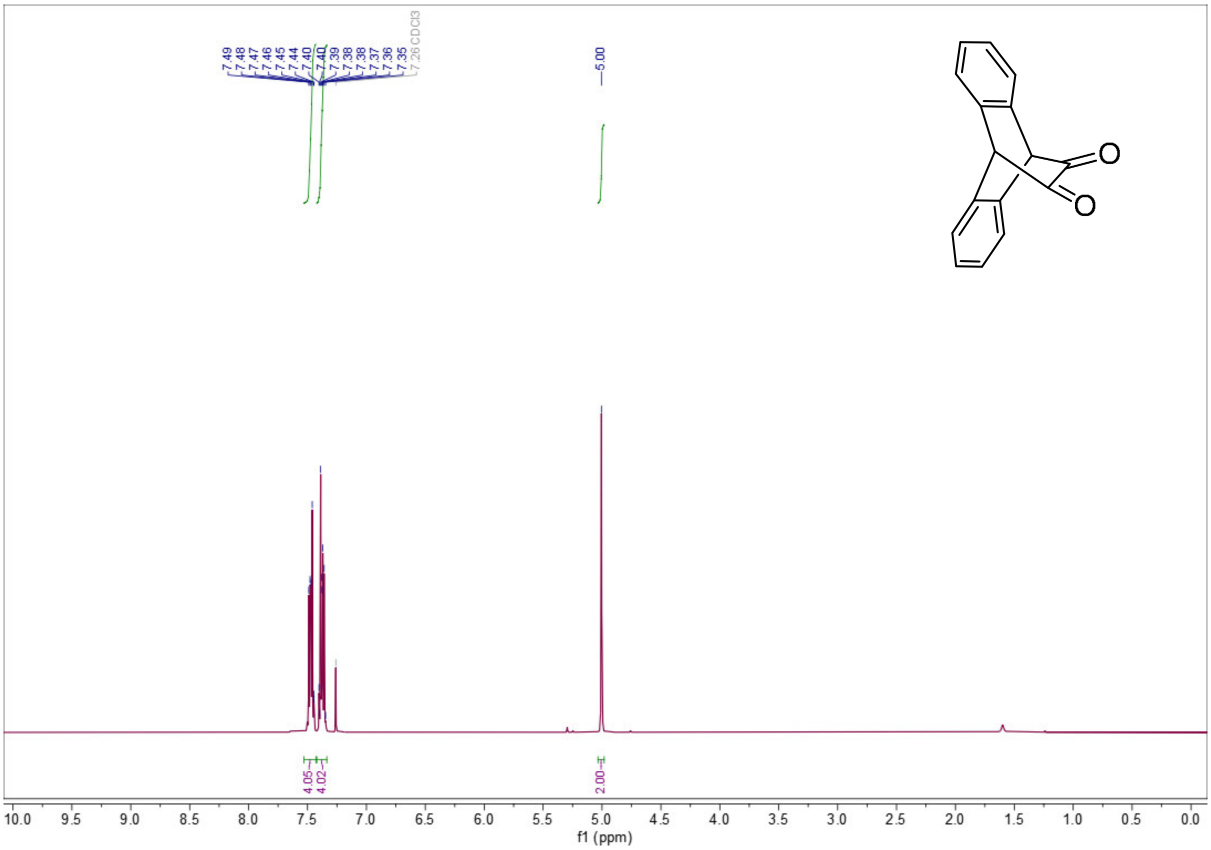

Figure S-13: $^{13}C\{^{1}H\}$ NMR ($CDCl_3$, 125 MHz) of **5**

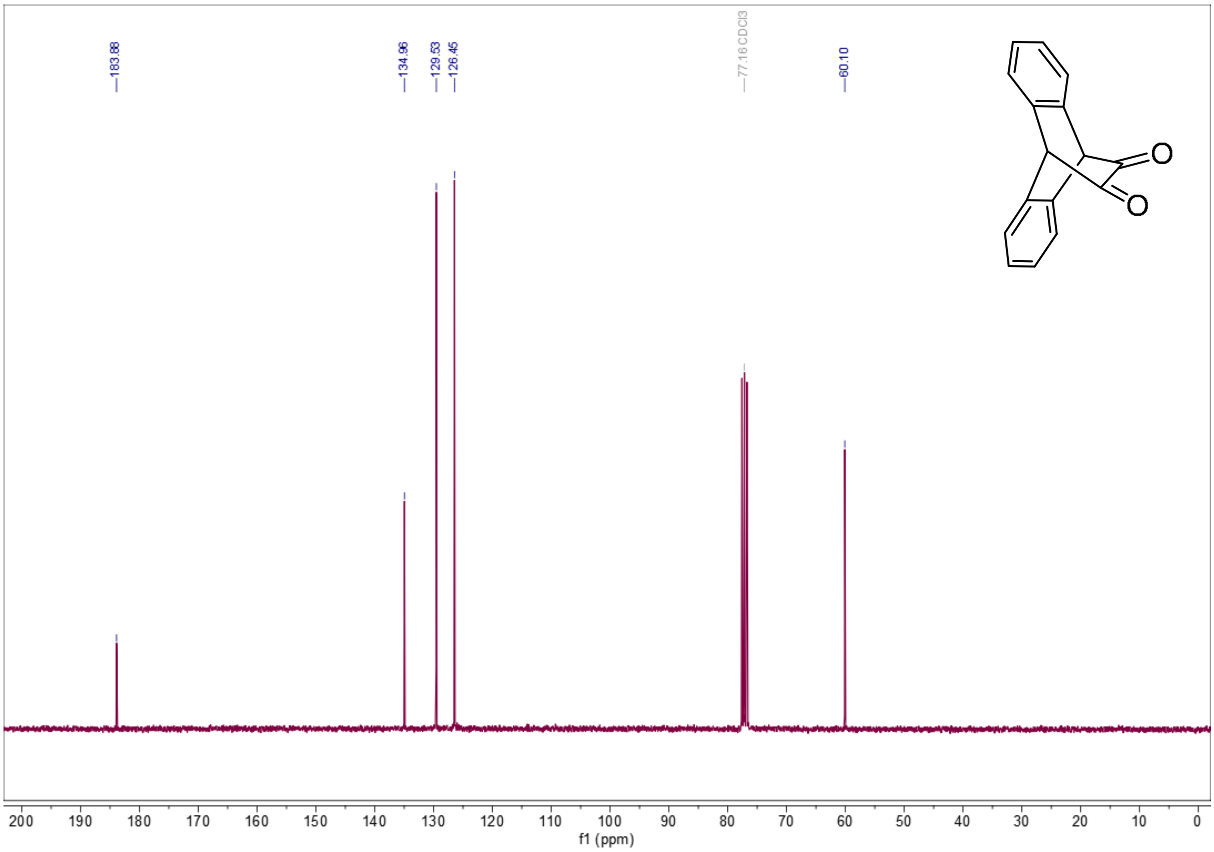

Figure S-14: ^{1}H NMR ($CDCl_3$, 300 MHz) of **7**

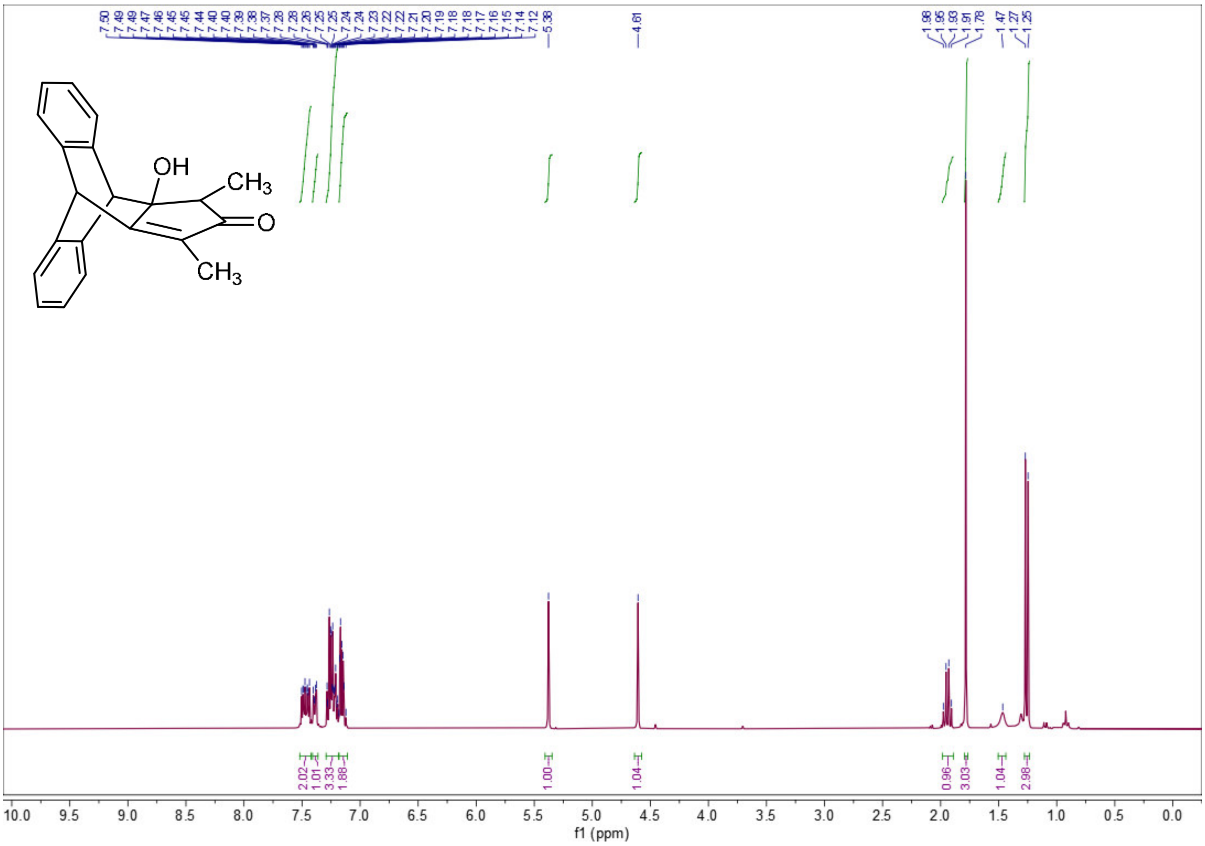

Figure S-15: $^{13}C\{^{1}H\}$ NMR ($CDCl_3$, 125 MHz) of **7**

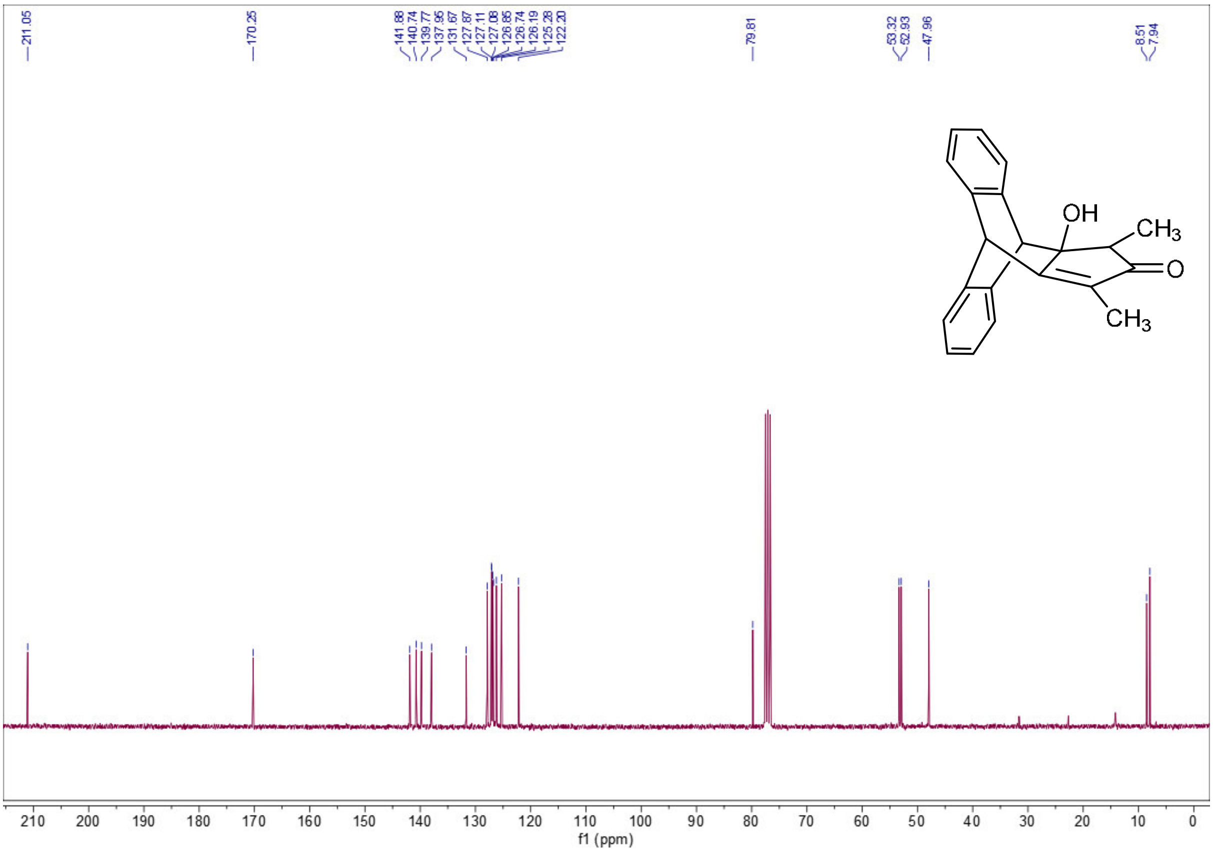

Figure S-16: ^{13}C-APT NMR ($CDCl_3$, 125 MHz) of **7**

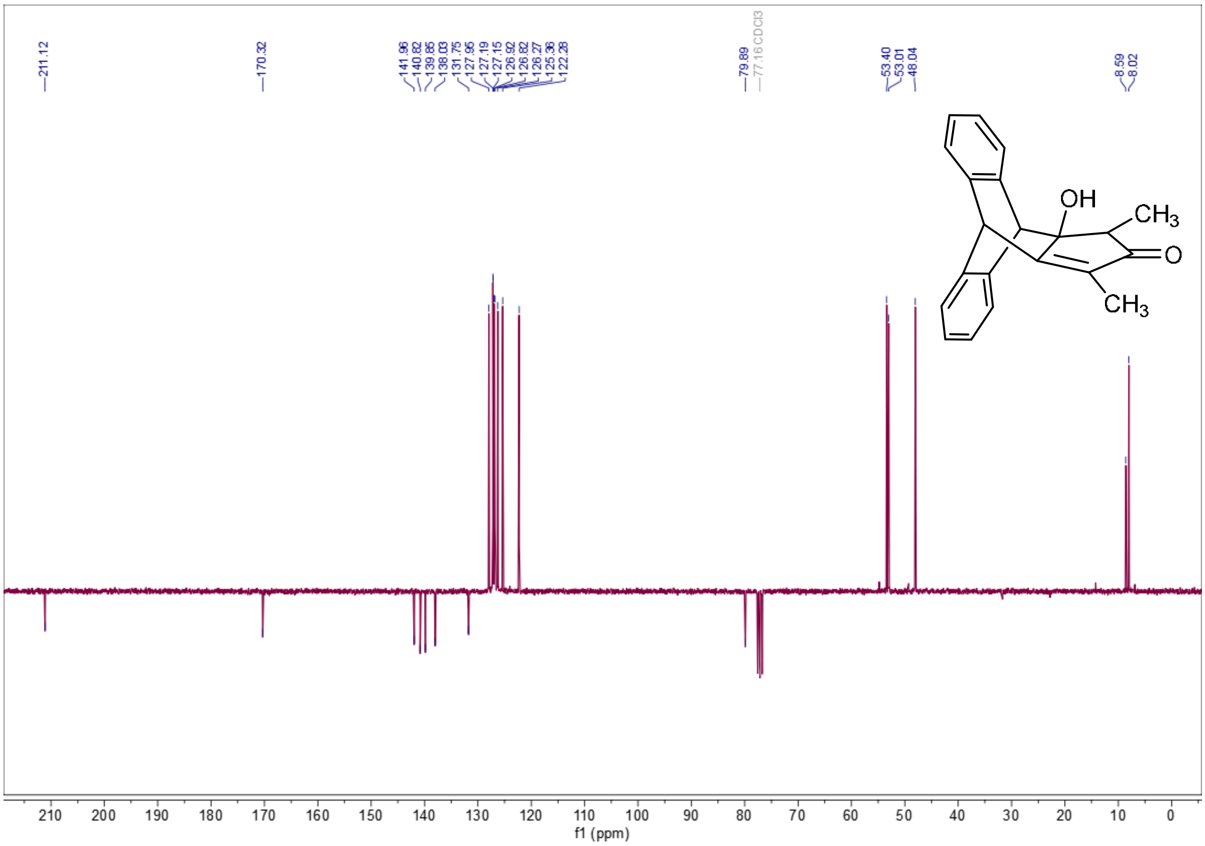

Figure S-17: H,H-COSY ($CDCl_3$, 300 MHz) of **7**

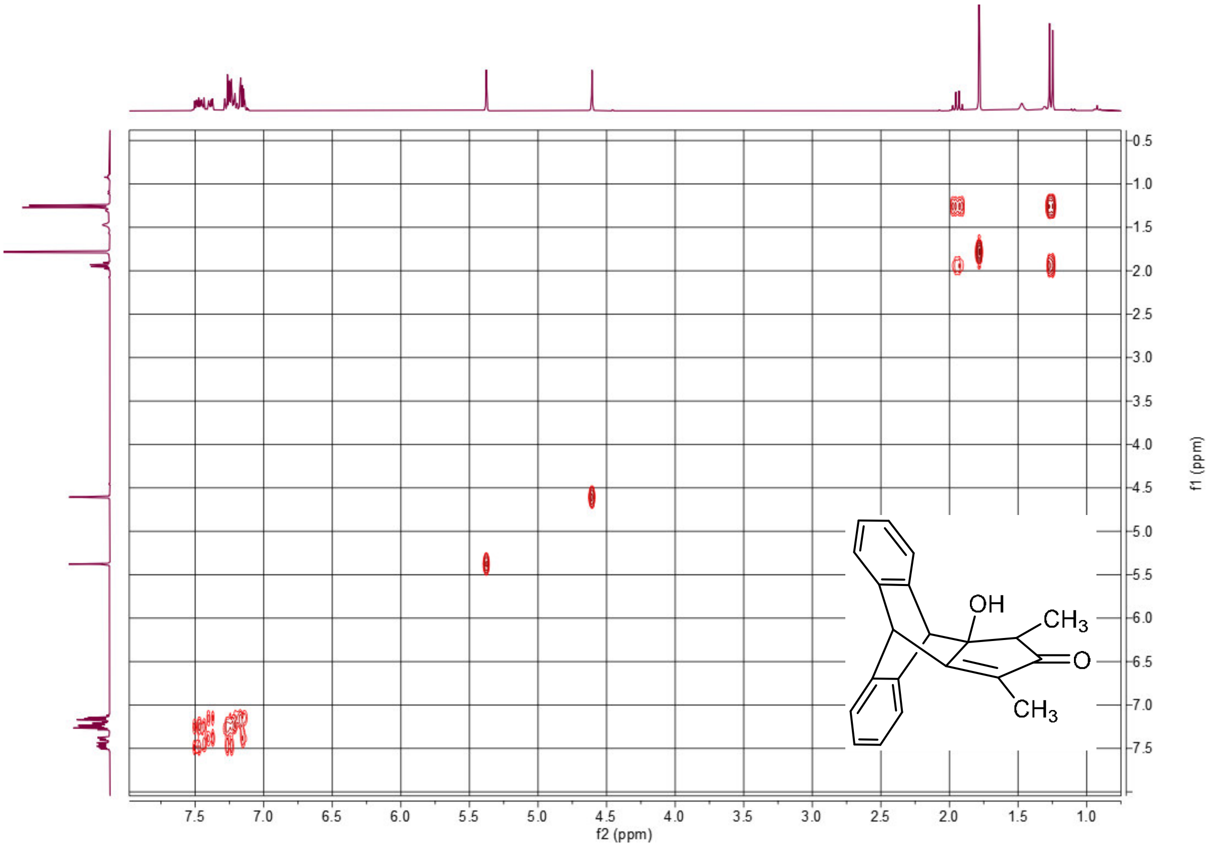

Figure S-18: HSQC ($CDCl_3$, 300 MHz) of 7

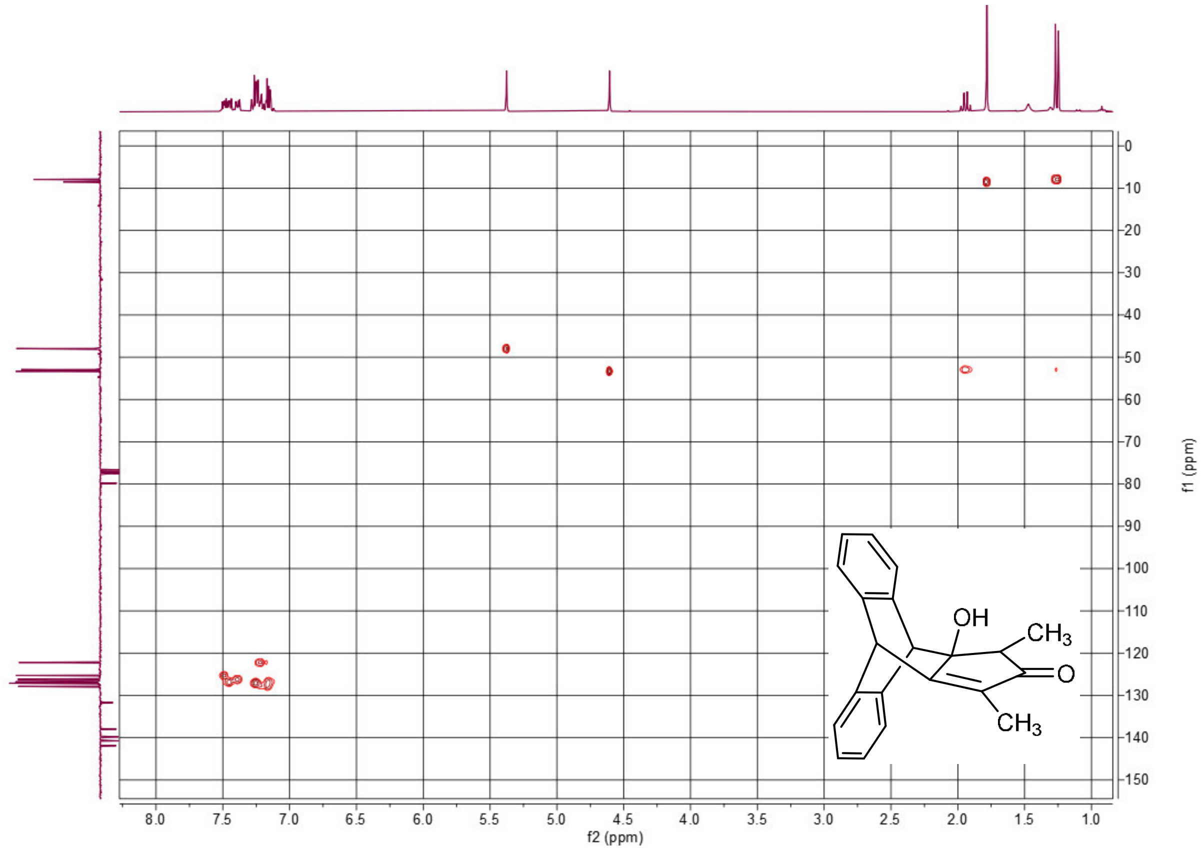

Figure S-19: ^{1}H NMR ($CDCl_3$, 300 MHz) of **8**

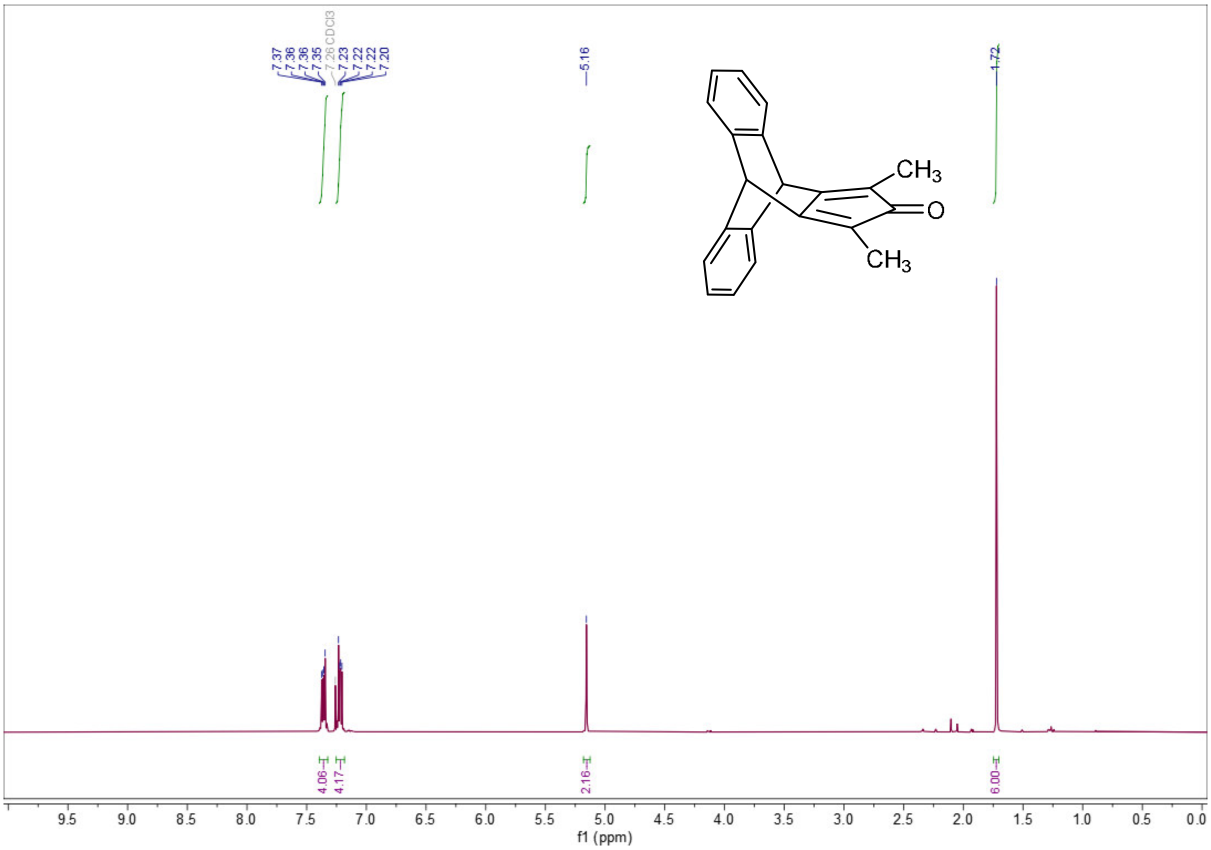

Figure S-20: $^{13}C\{^{1}H\}$ NMR ($CDCl_3$, 125 MHz) of **8**

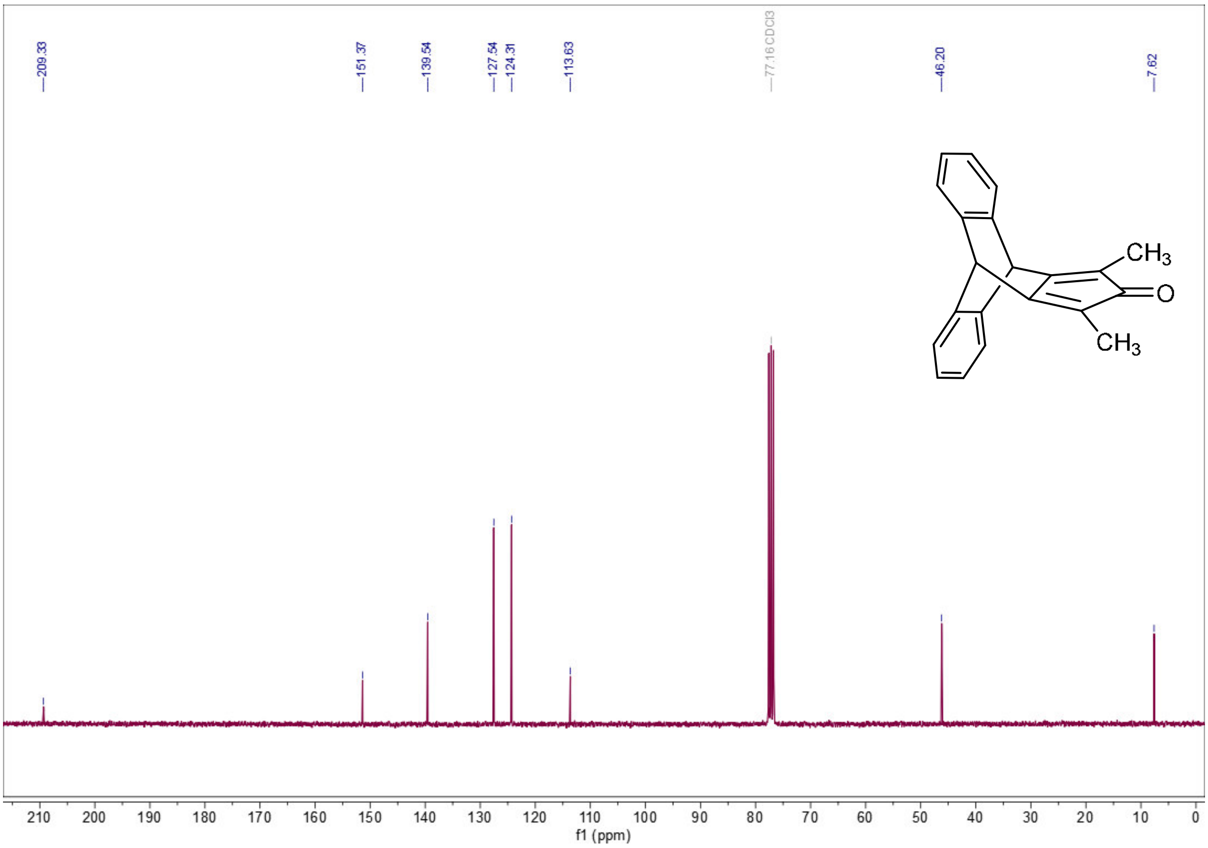

Figure S-21: ^{1}H NMR ($CDCl_3$, 300 MHz) of *exo*-**8**

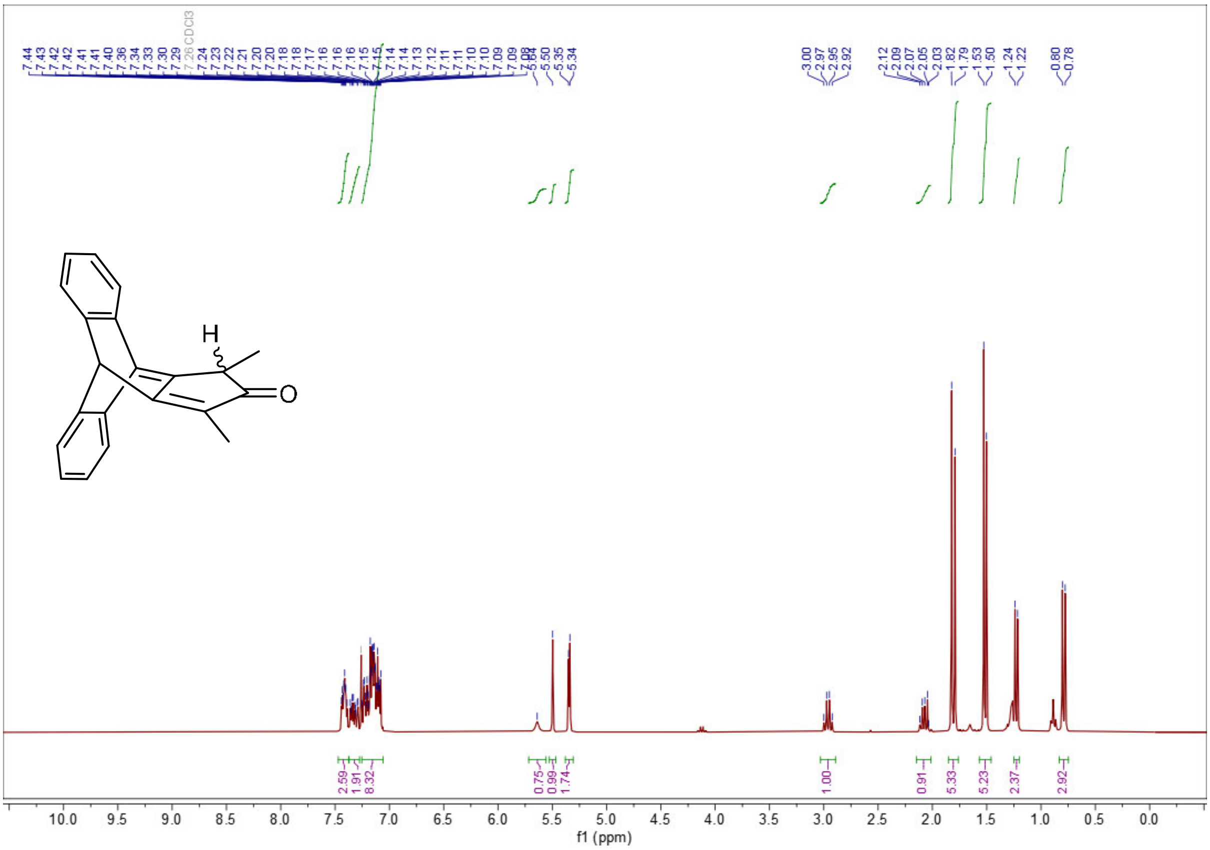

Figure S-22: ^{1}H NMR ($CDCl_3$, 300 MHz) of **9**

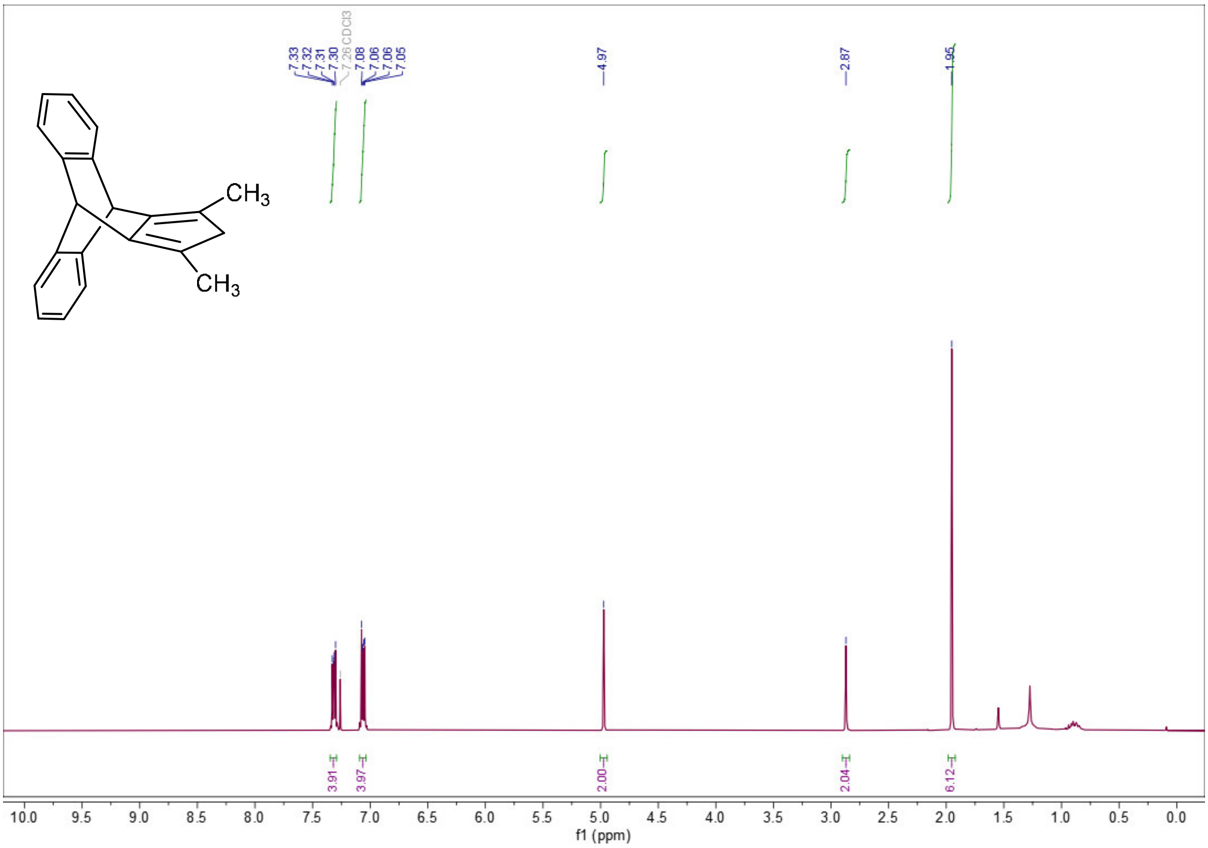

Figure S-23: $^{13}C\{^{1}H\}$ NMR ($CDCl_3$, 125 MHz) of **9**

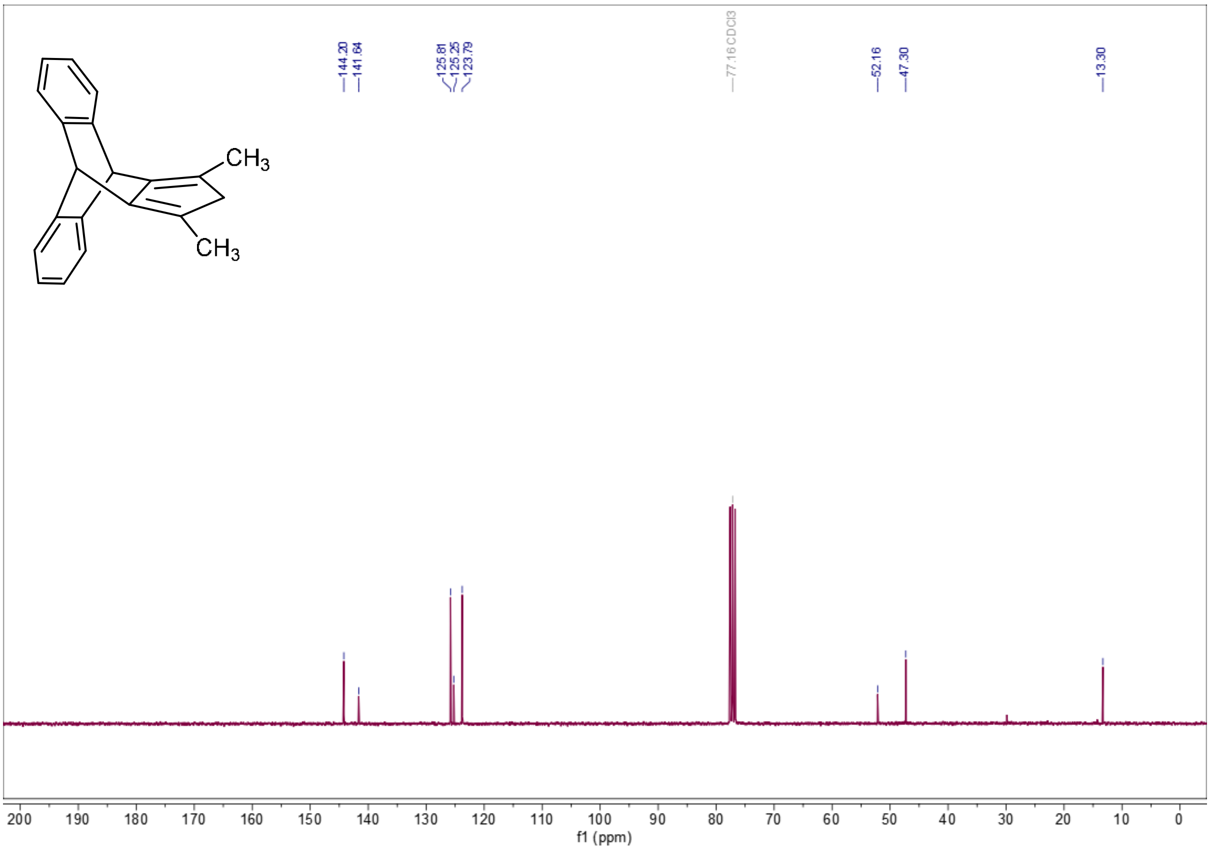

Figure S-24: ^{13}C-APT NMR ($CDCl_3$, 125 MHz) of **9**

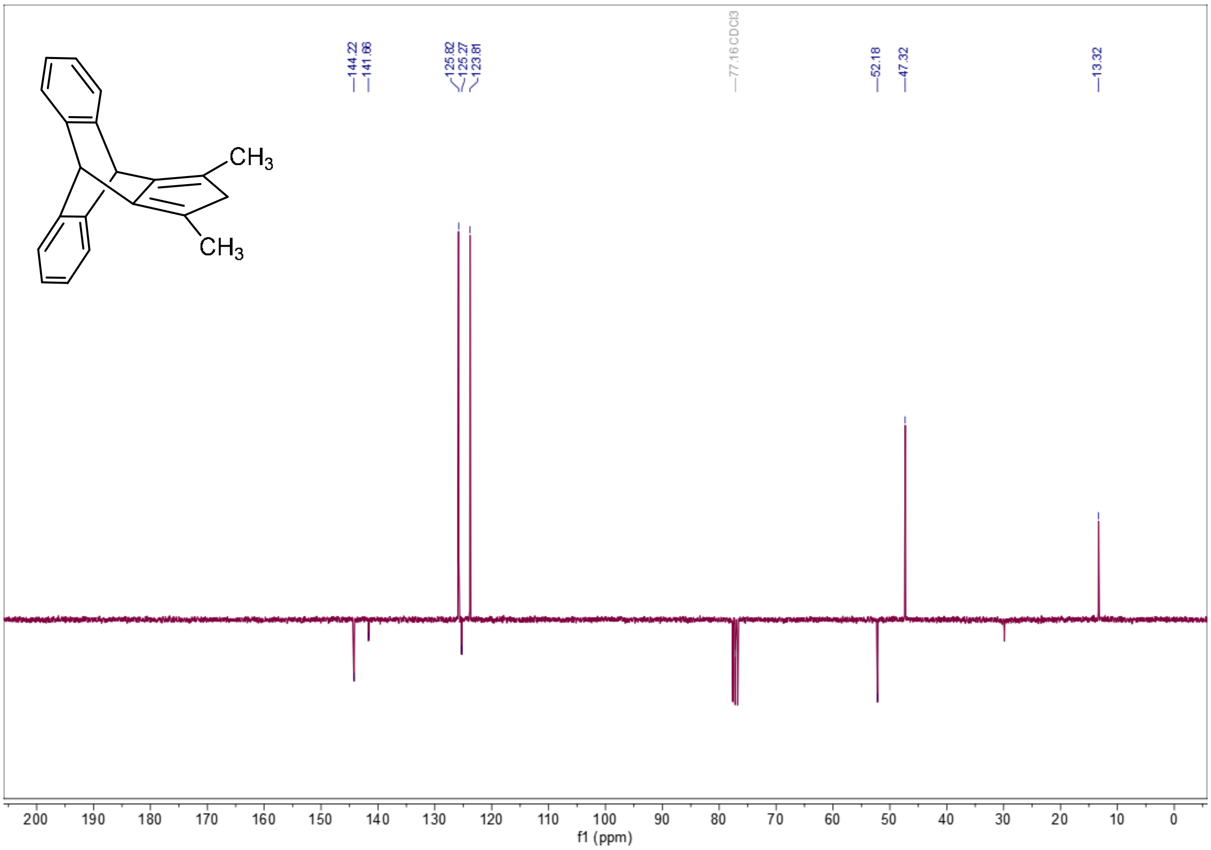

Figure S-25: H,H-COSY ($CDCl_3$, 300 MHz) of **9**

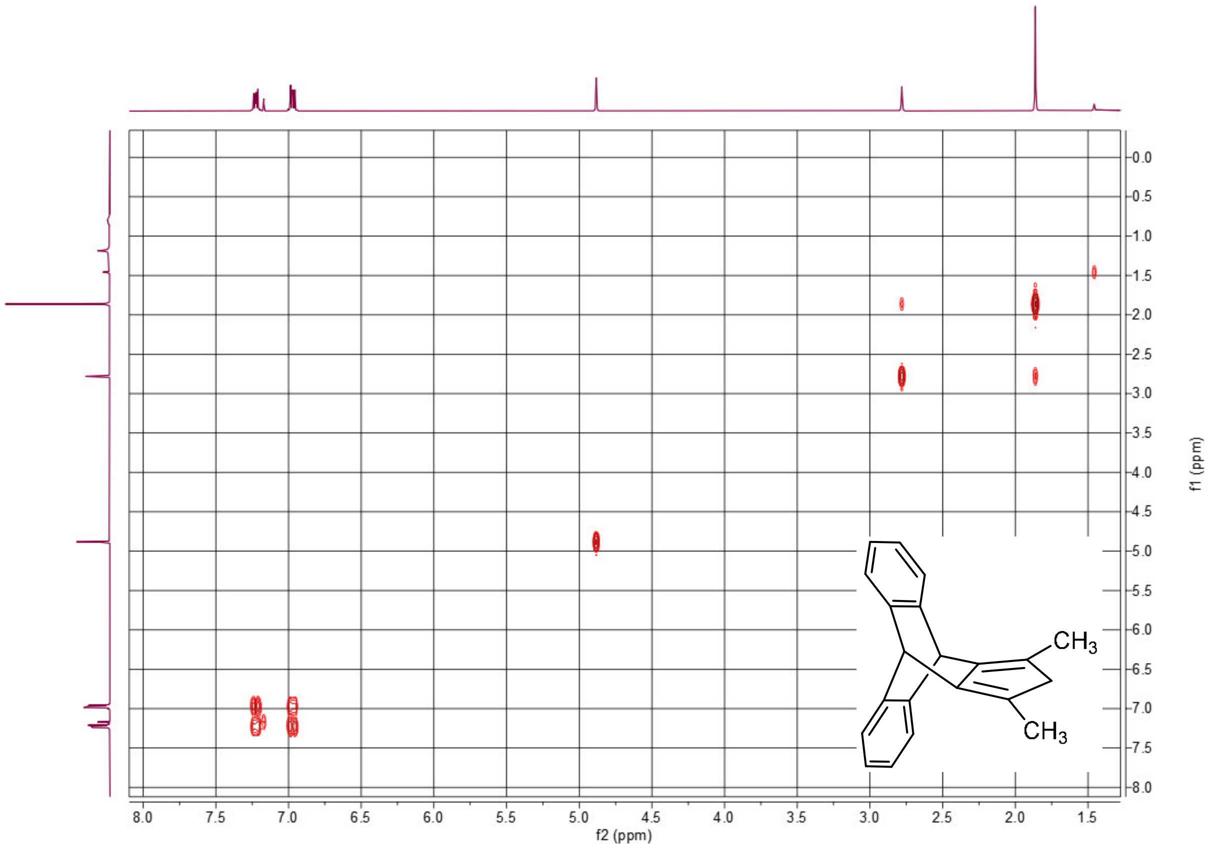

Figure S-26: HSQC ($CDCl_3$, 300 MHz) of **9**

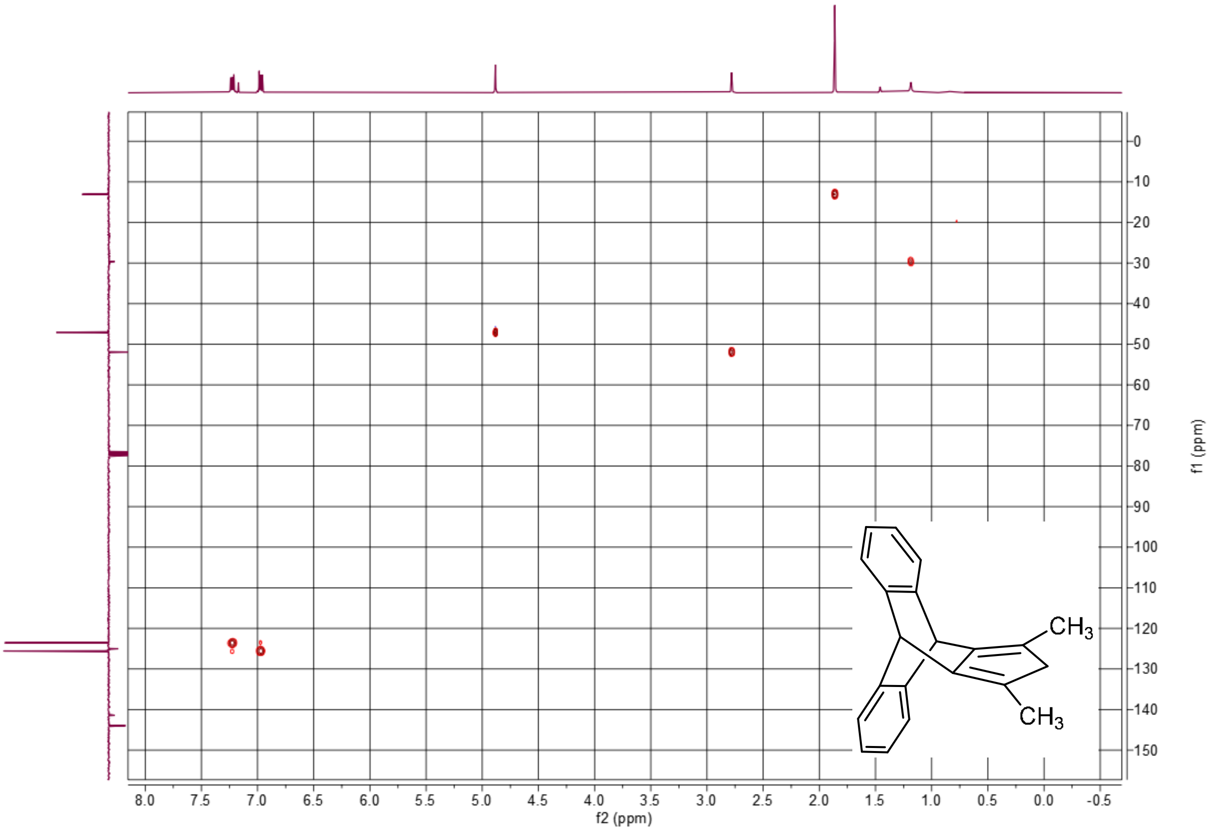

Figure S-27: ^{1}H NMR ($CDCl_3$, 300 MHz) of **9**-OH

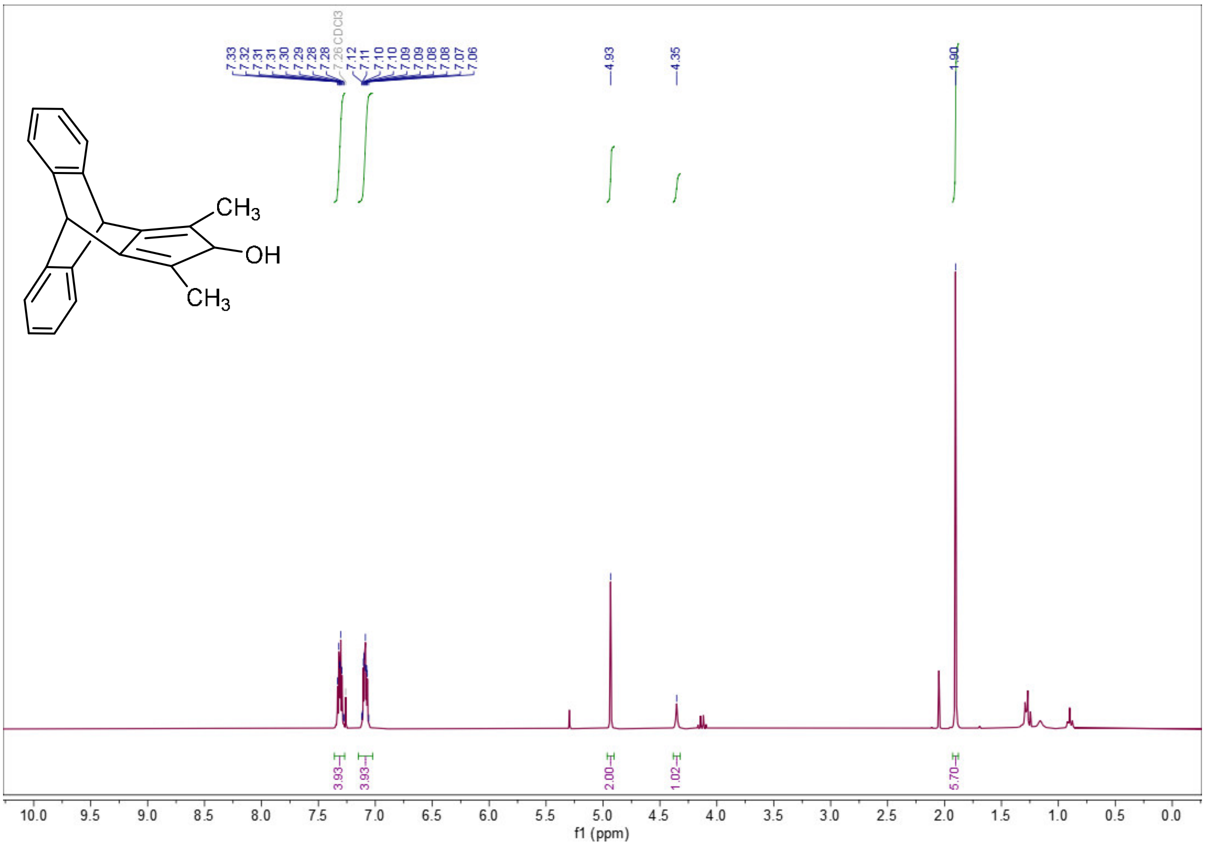

Figure S-28: ^{1}H NMR ($CDCl_3$, 300 MHz) of **11**

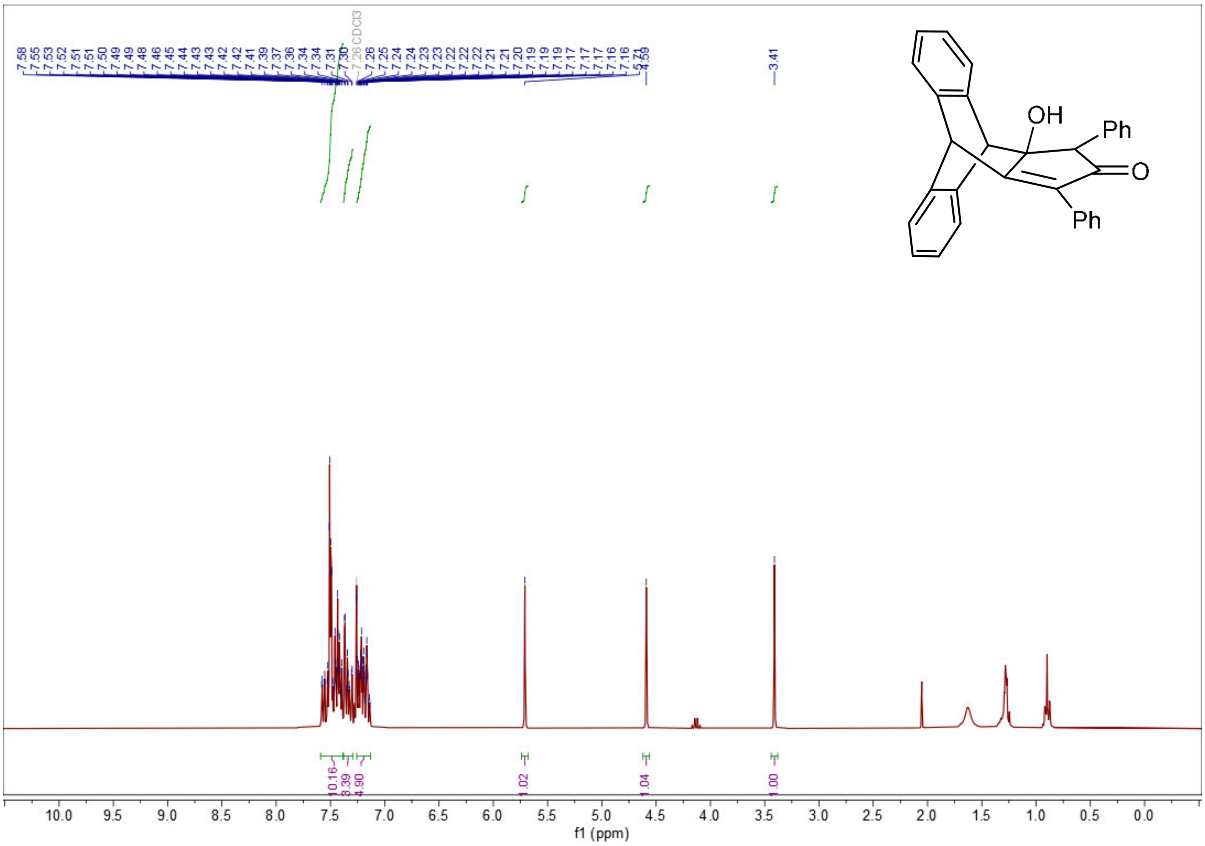

Figure S-29: ^{13}C-APT NMR ($CDCl_3$, 125 MHz) of **11**

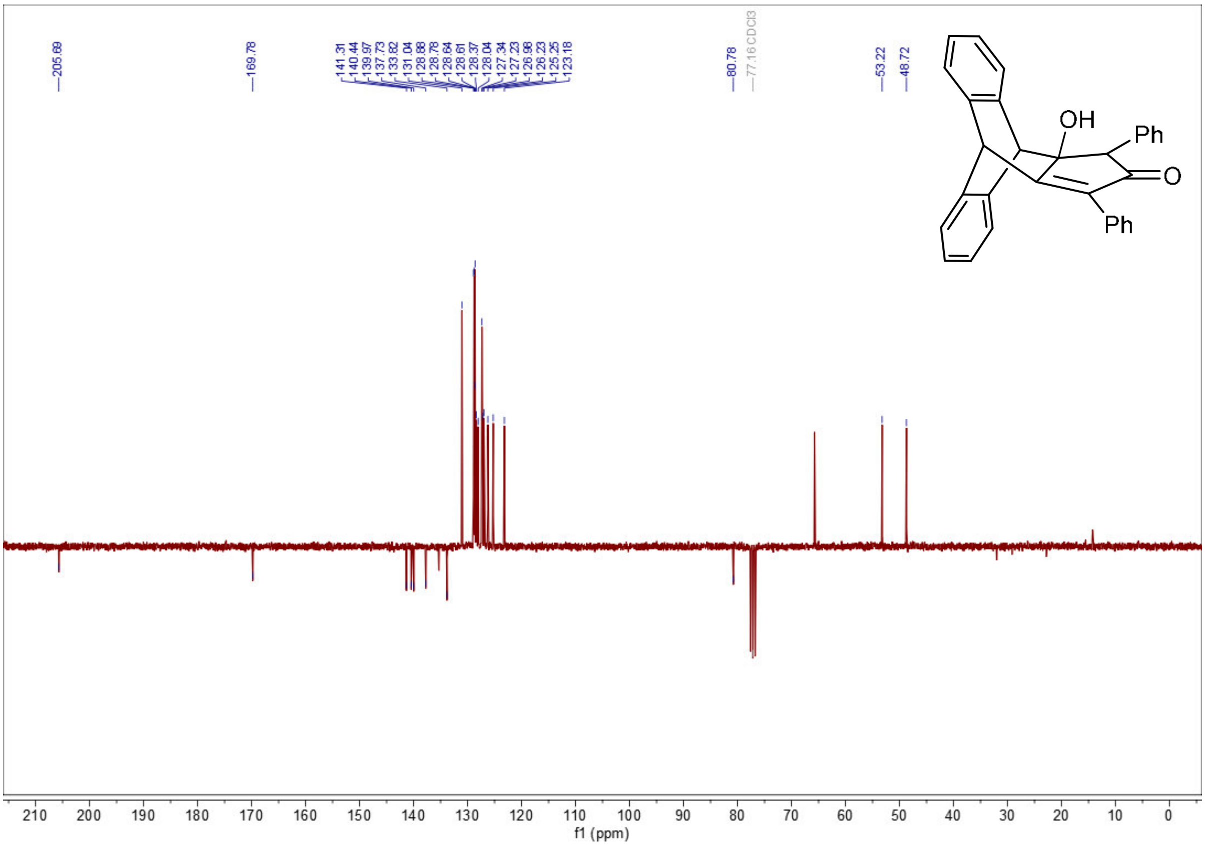

Figure S-30: ^{1}H NMR (CDCl$_3$, 300 MHz) of **12**

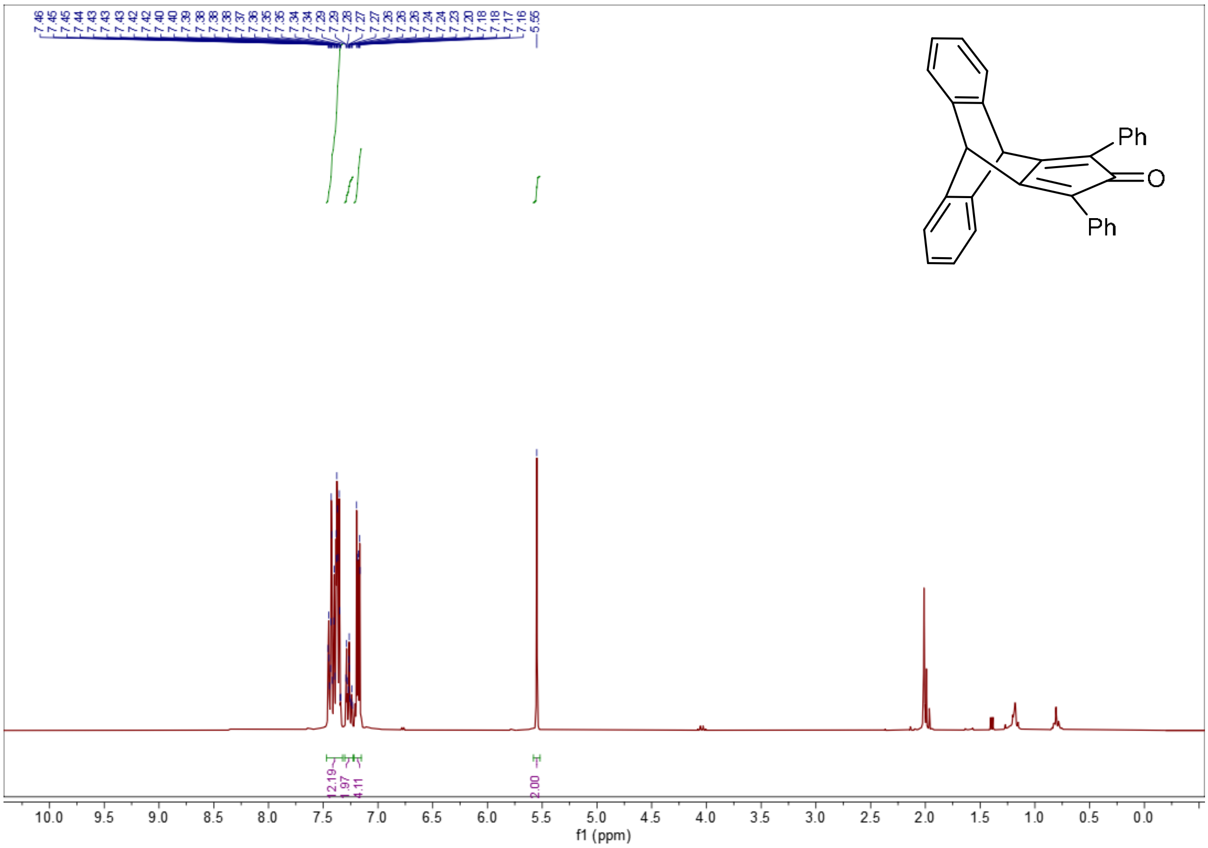

Figure S-31: ^{13}C{^{1}H} NMR (CDCl$_3$, 125 MHz) of **12**

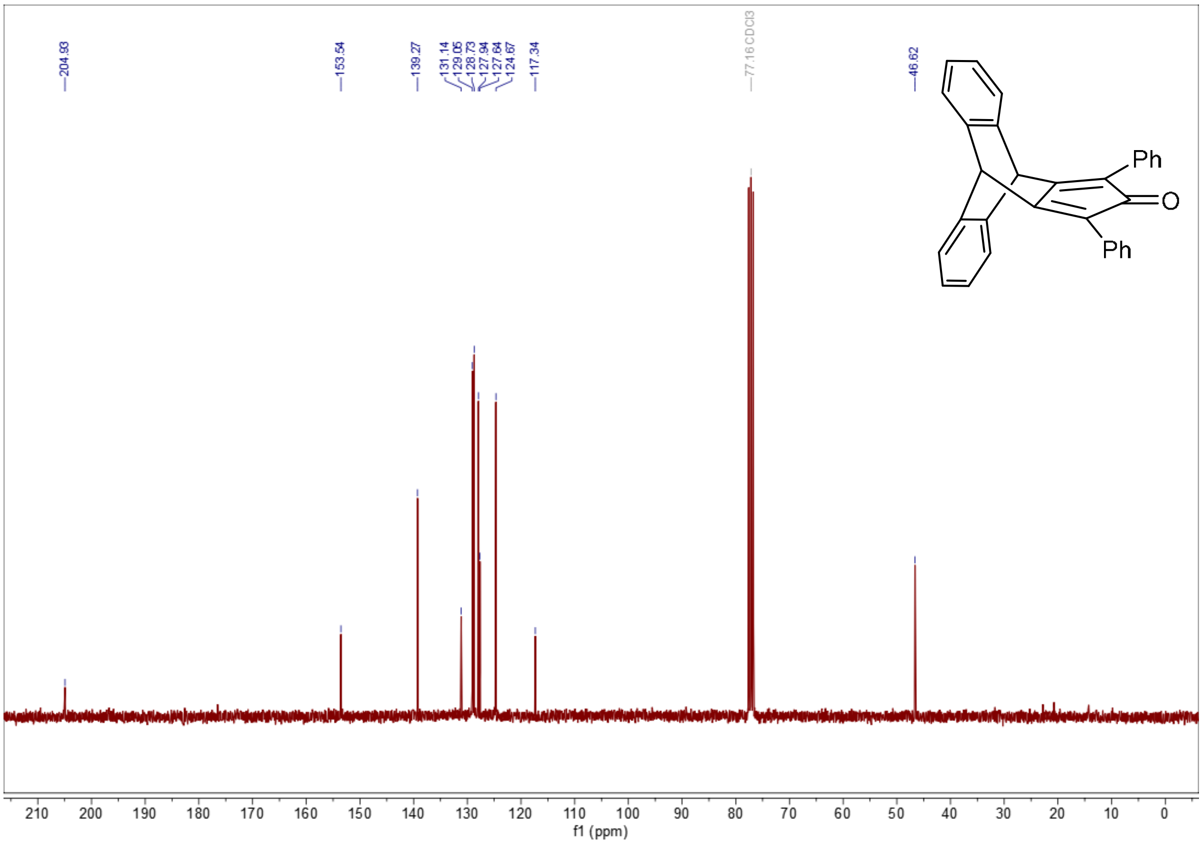

Figure S-32: ^{13}C-APT NMR ($CDCl_3$, 125 MHz) of **12**

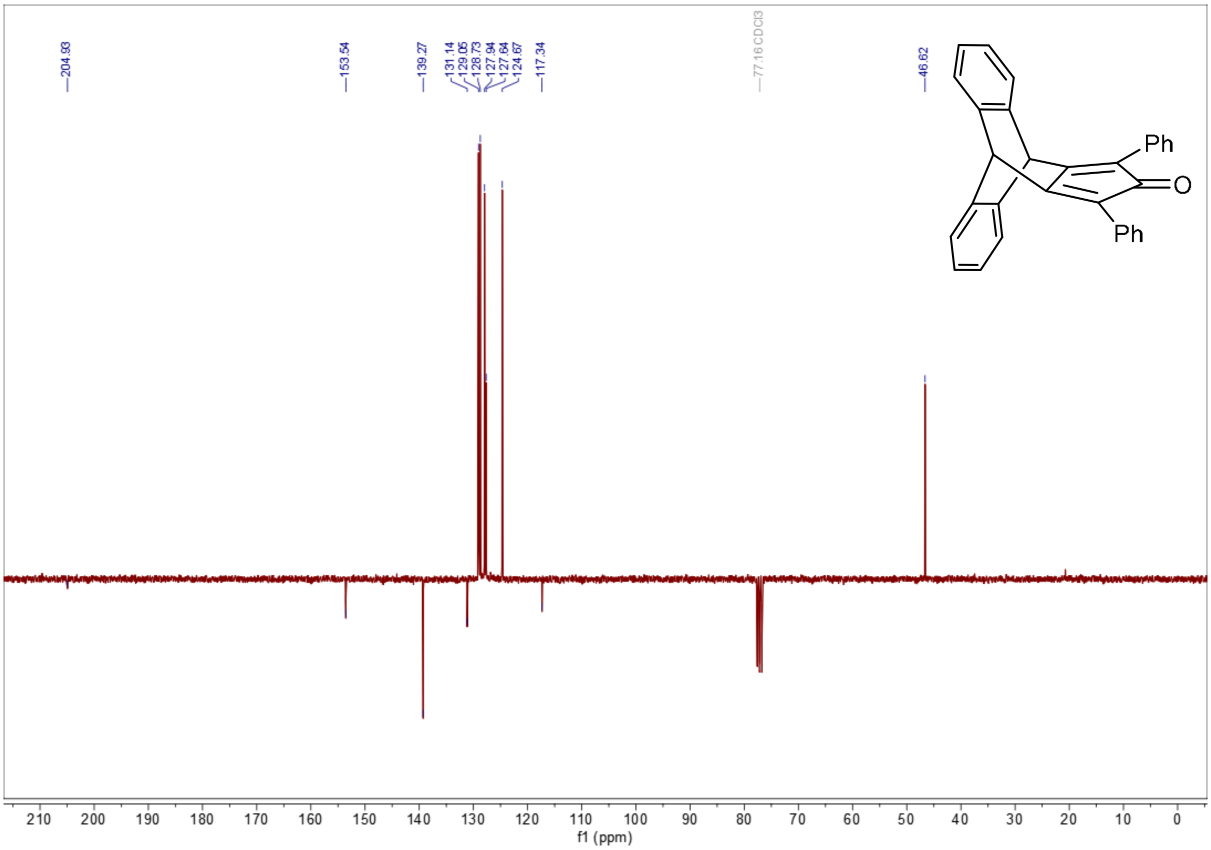

Figure S-33: H,H-COSY ($CDCl_3$, 300 MHz) of **12**

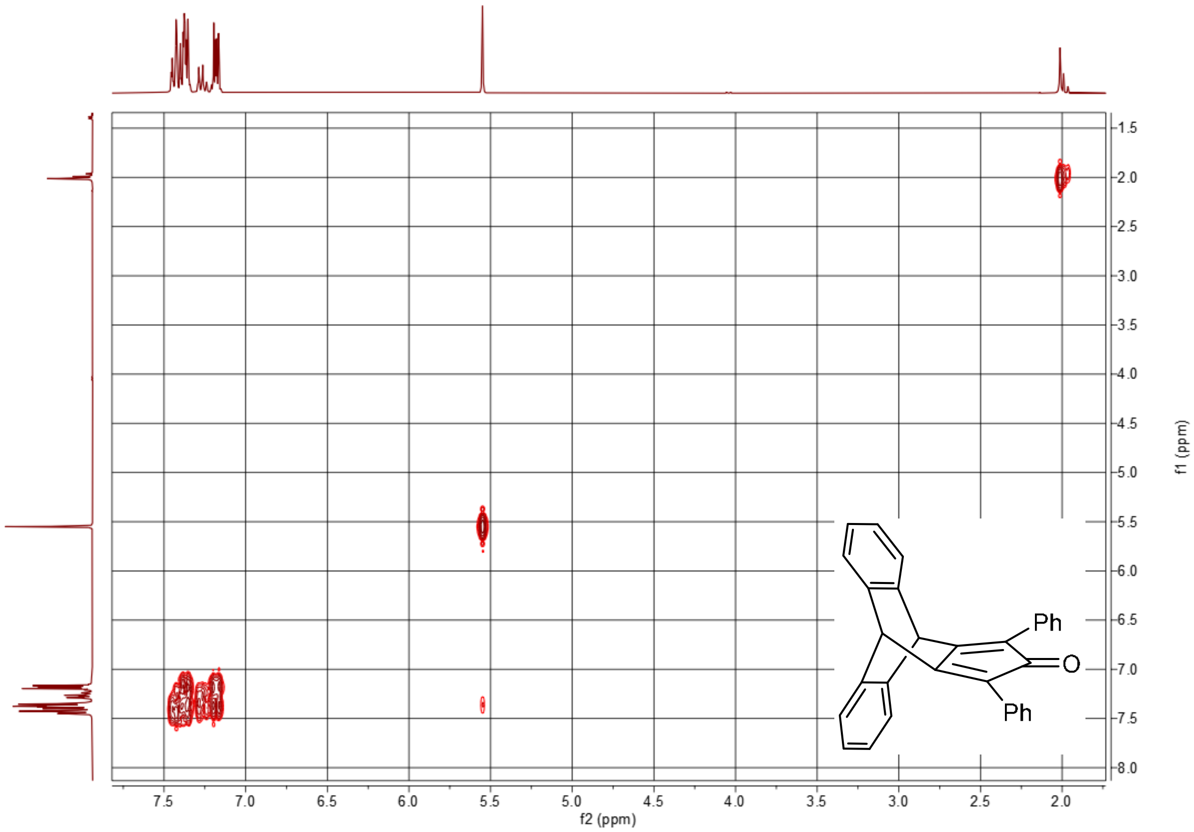

Figure S-34: HSQC ($CDCl_3$, 300 MHz) of **12**

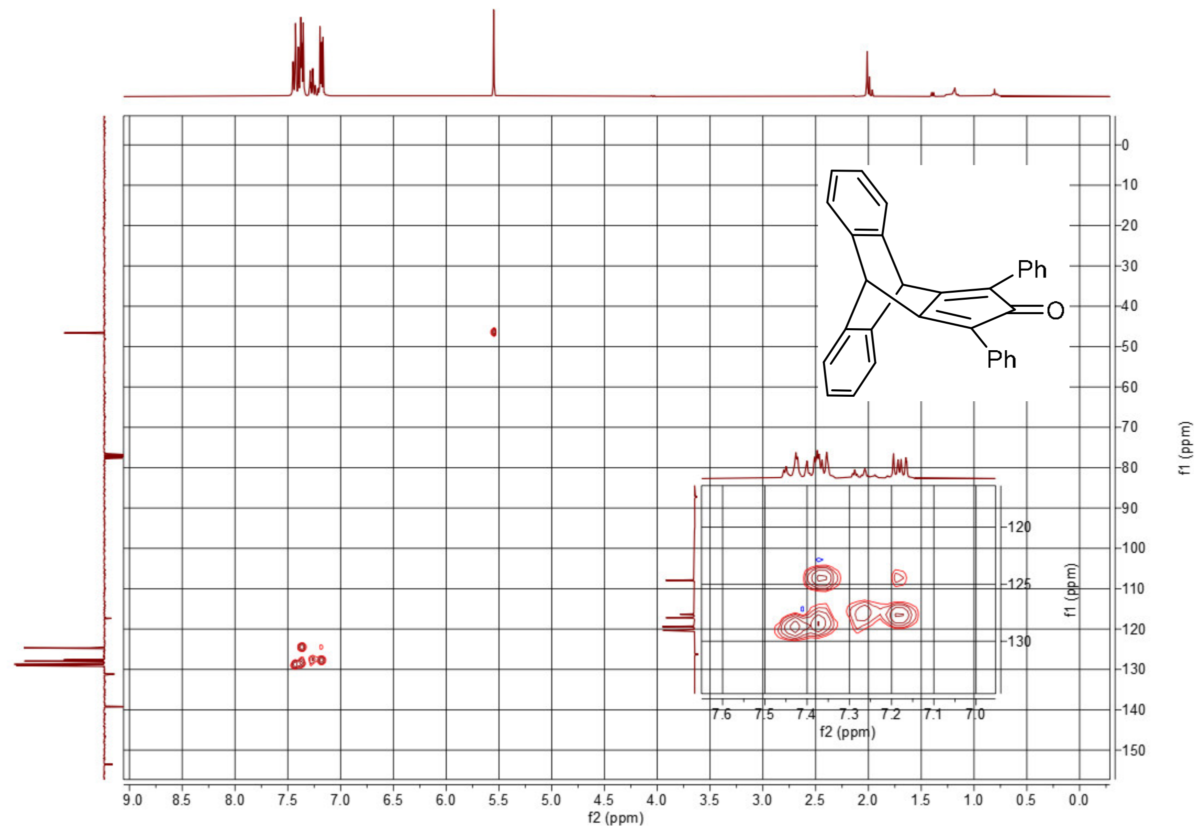

Figure S-35: ^1H NMR ($CDCl_3$, 300 MHz) of **13**

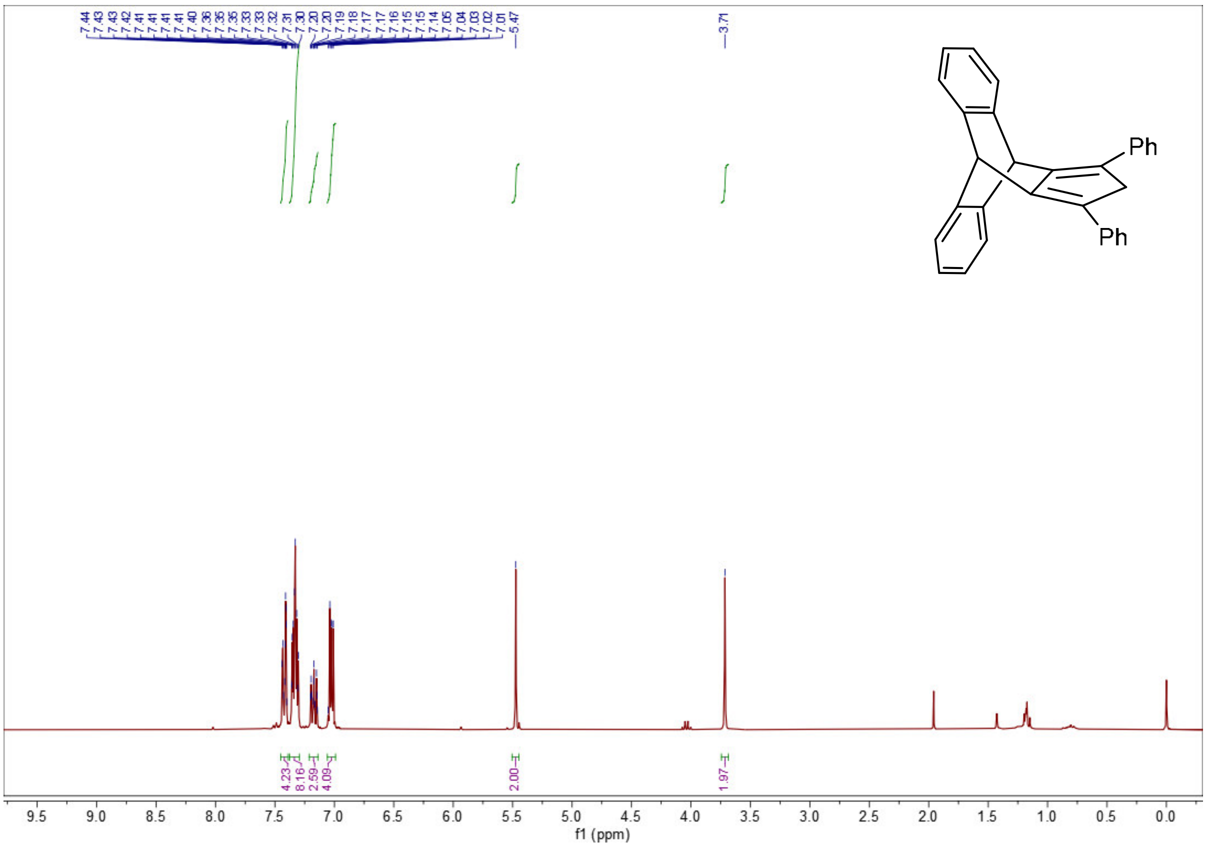

Figure S-36: $^{13}C\{^{1}H\}$ NMR ($CDCl_3$, 125 MHz) of **13**

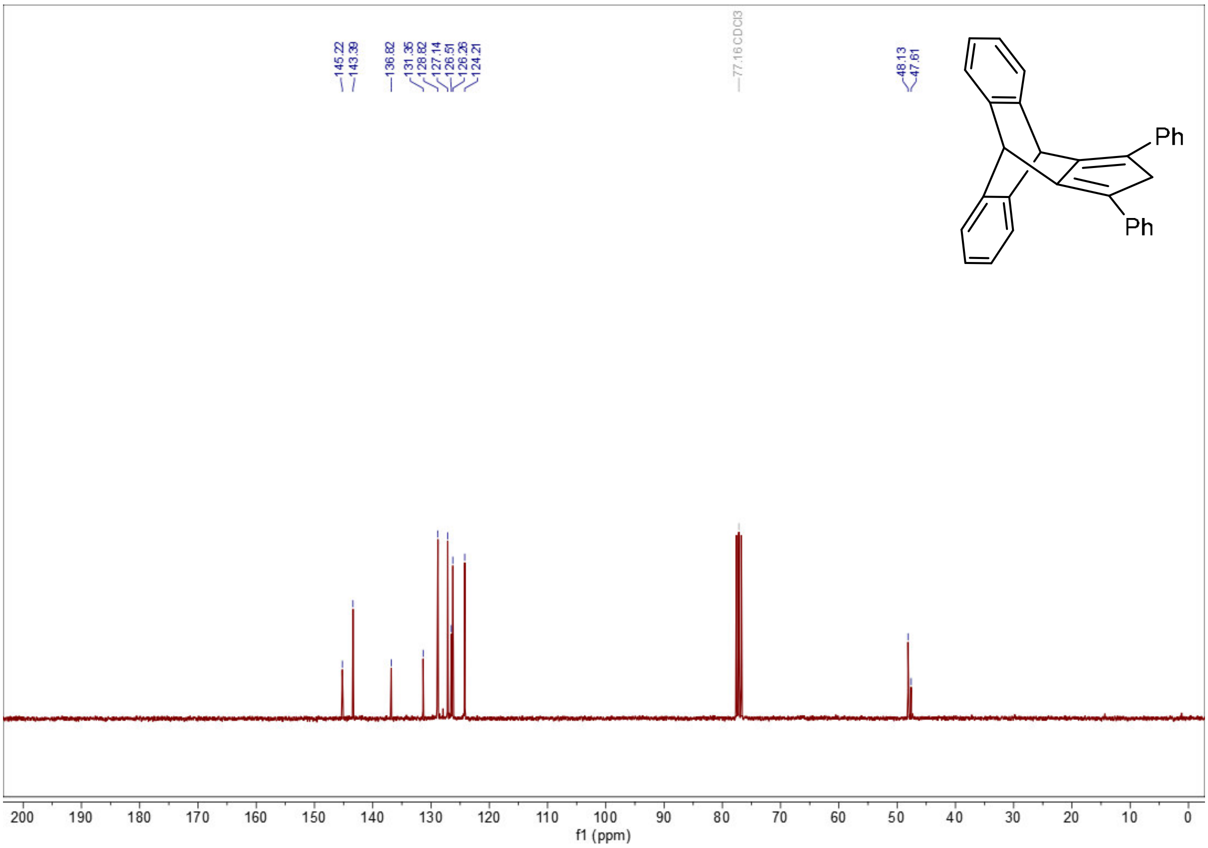

Figure S-37: ^{13}C-APT NMR ($CDCl_3$, 125 MHz) of **13**

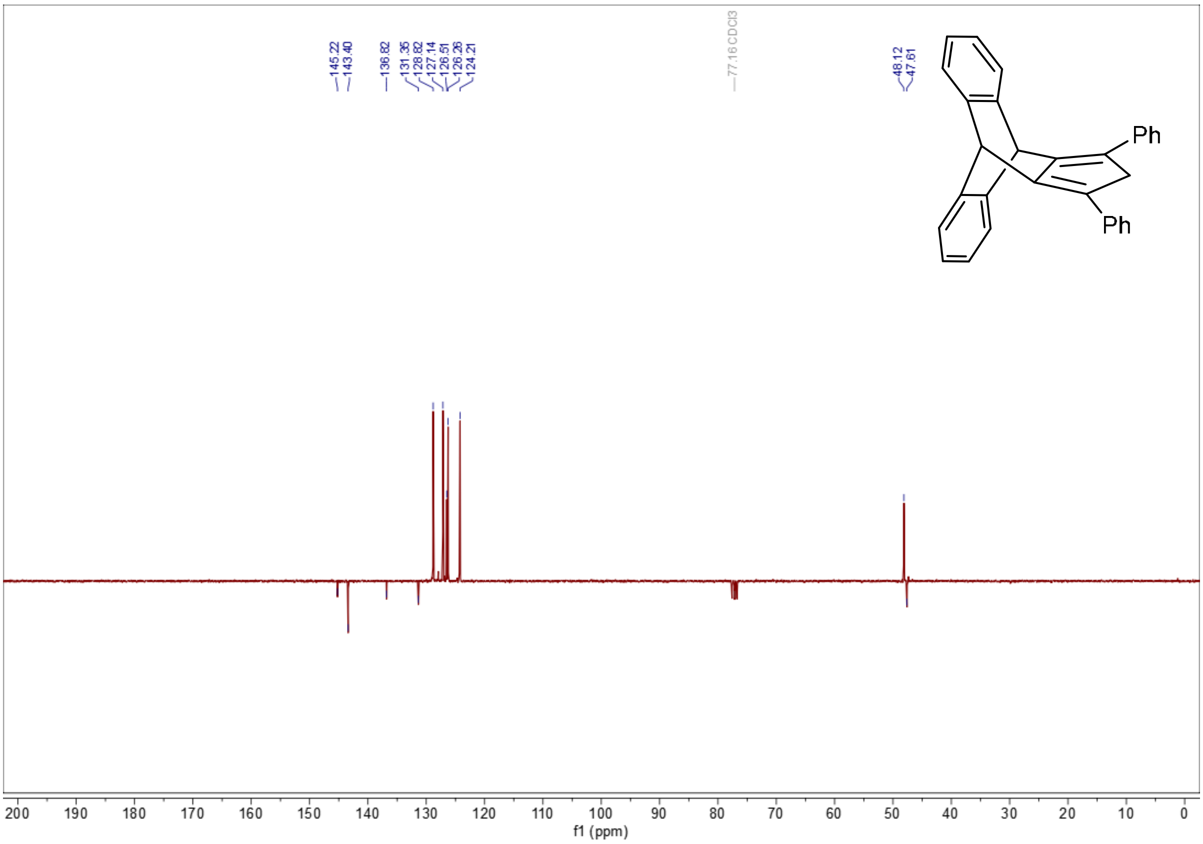

Figure S-38: COSY ($CDCl_3$, 300 MHz) of **13**

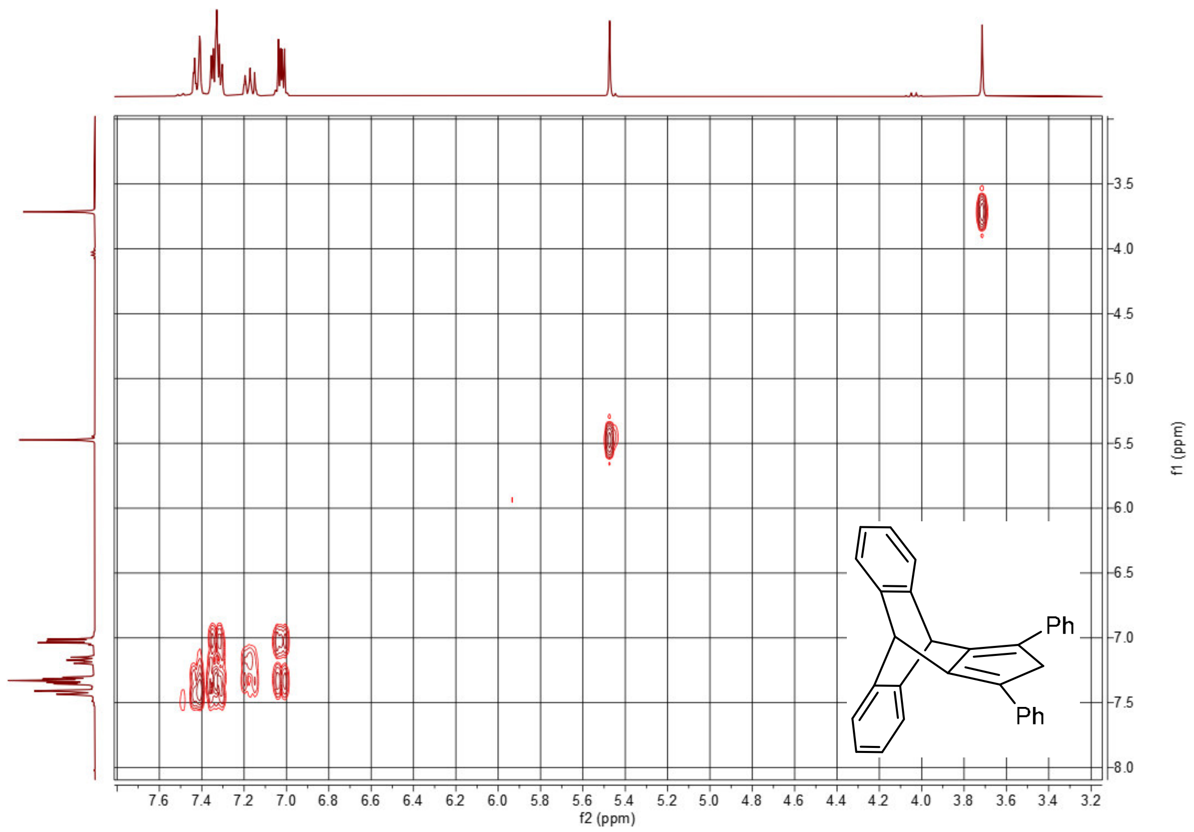

Figure S-39: ^{1}H NMR ($CDCl_3$, 300 MHz) of **13**-OH

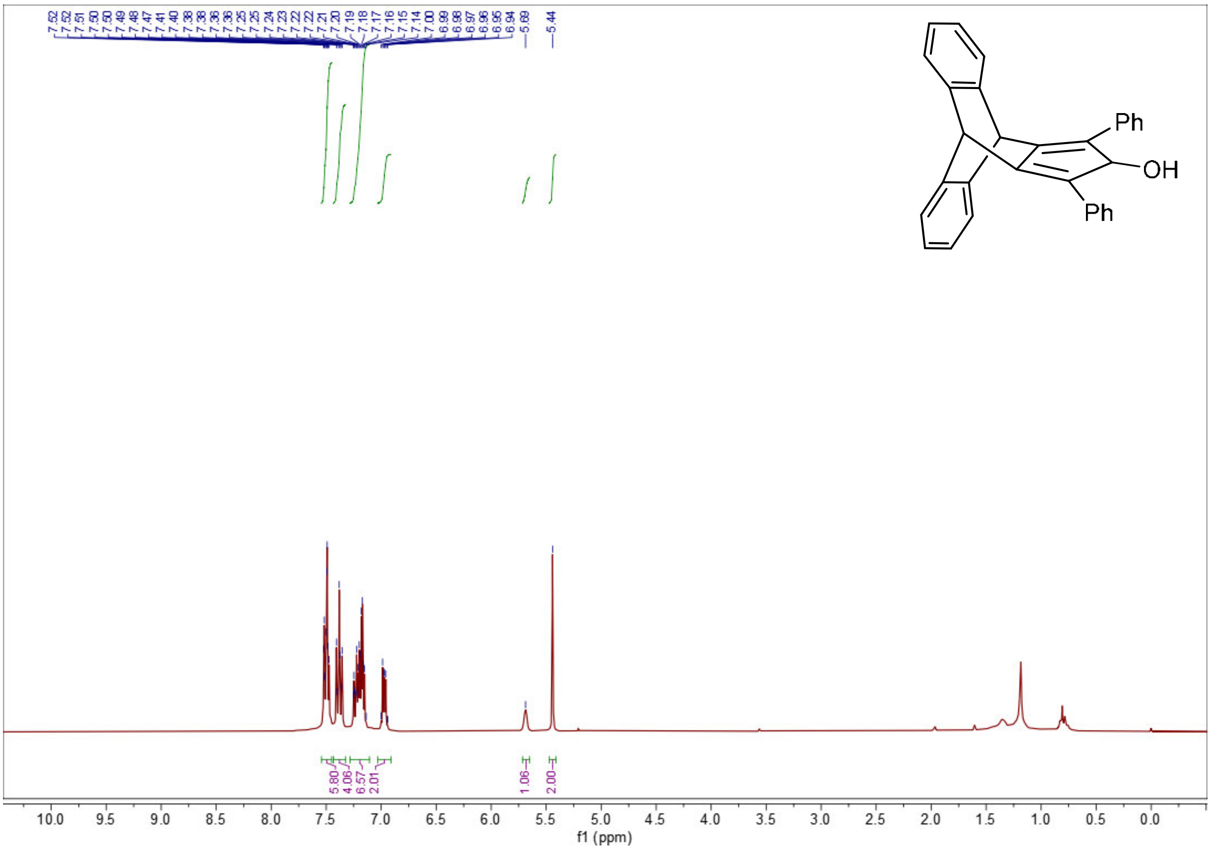

Figure S-40: ^{13}C-APT NMR ($CDCl_3$, 125 MHz) of **13**-OH

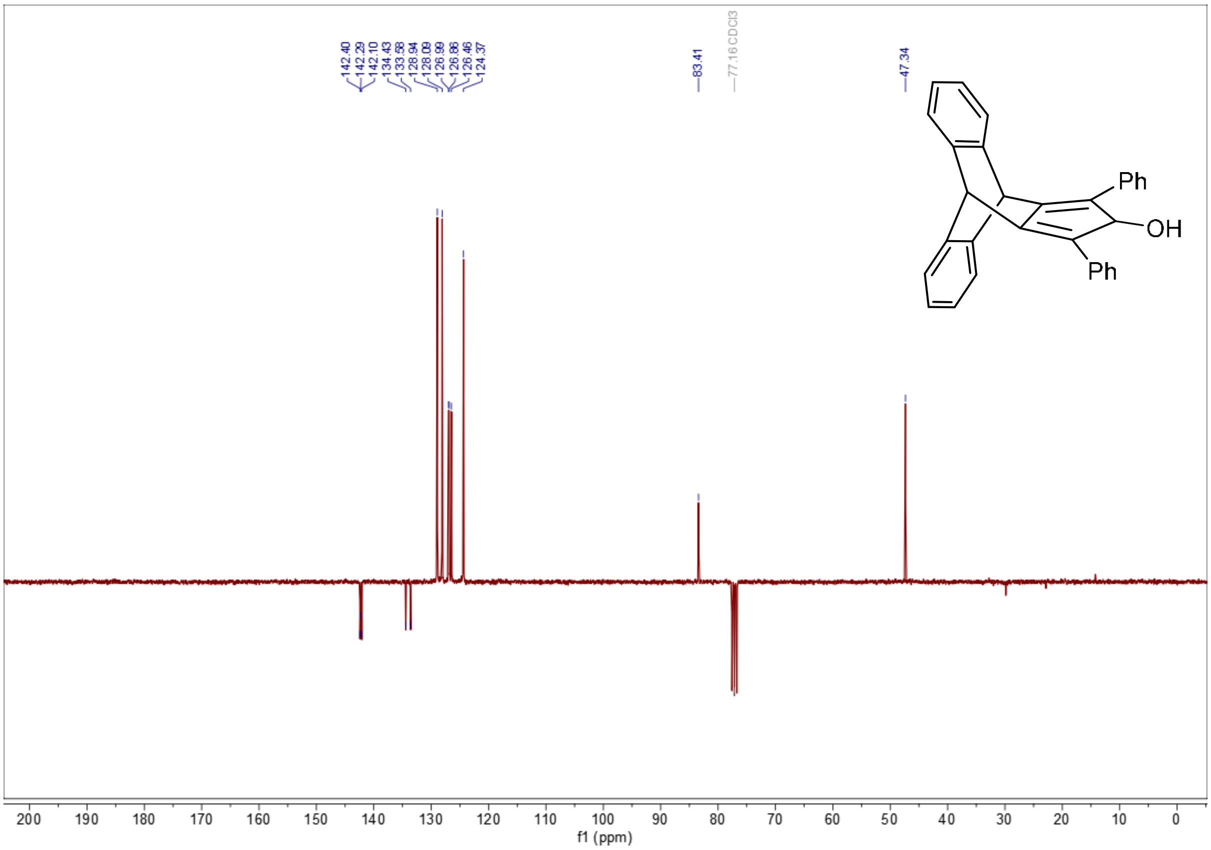

Figure S-41: ^{1}H NMR ($CDCl_3$, 300 MHz) of **14**

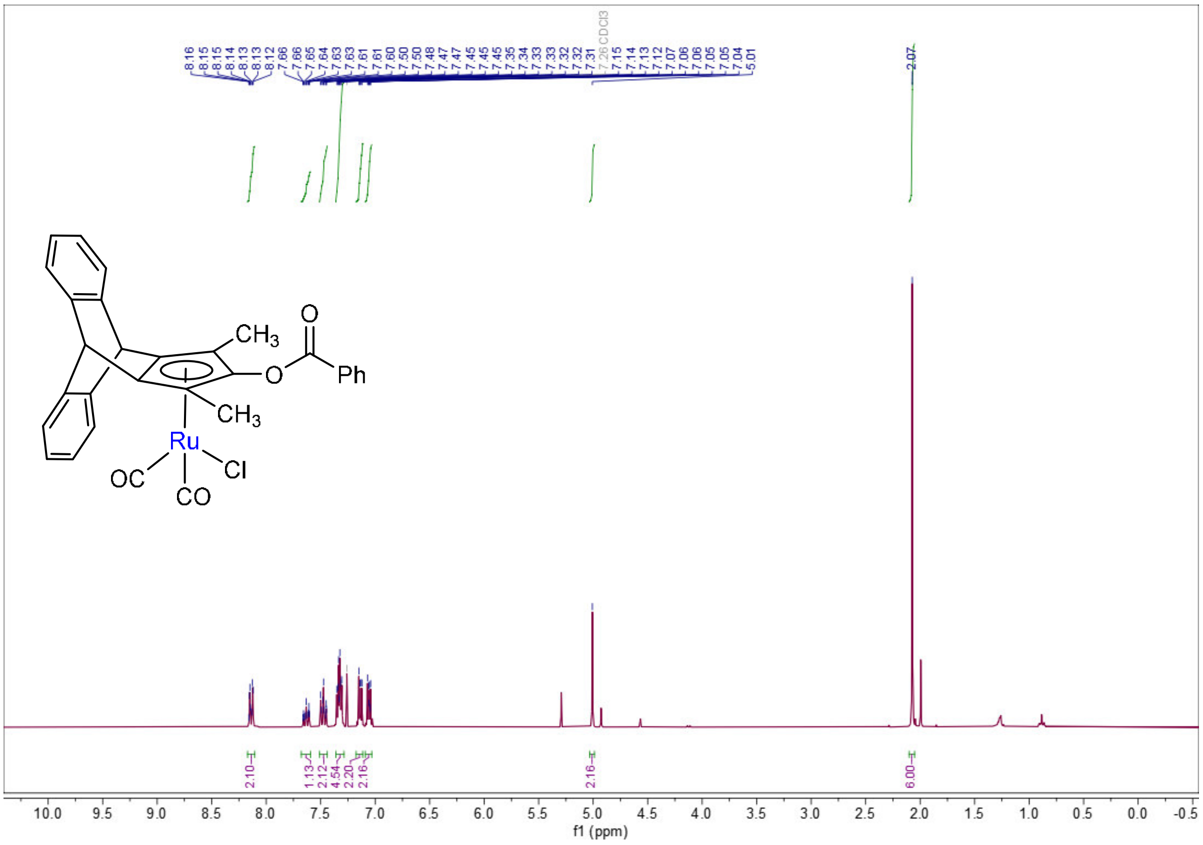

Figure S-42: $^{13}C\{^{1}H\}$ NMR ($CDCl_3$, 125 MHz) of **14**

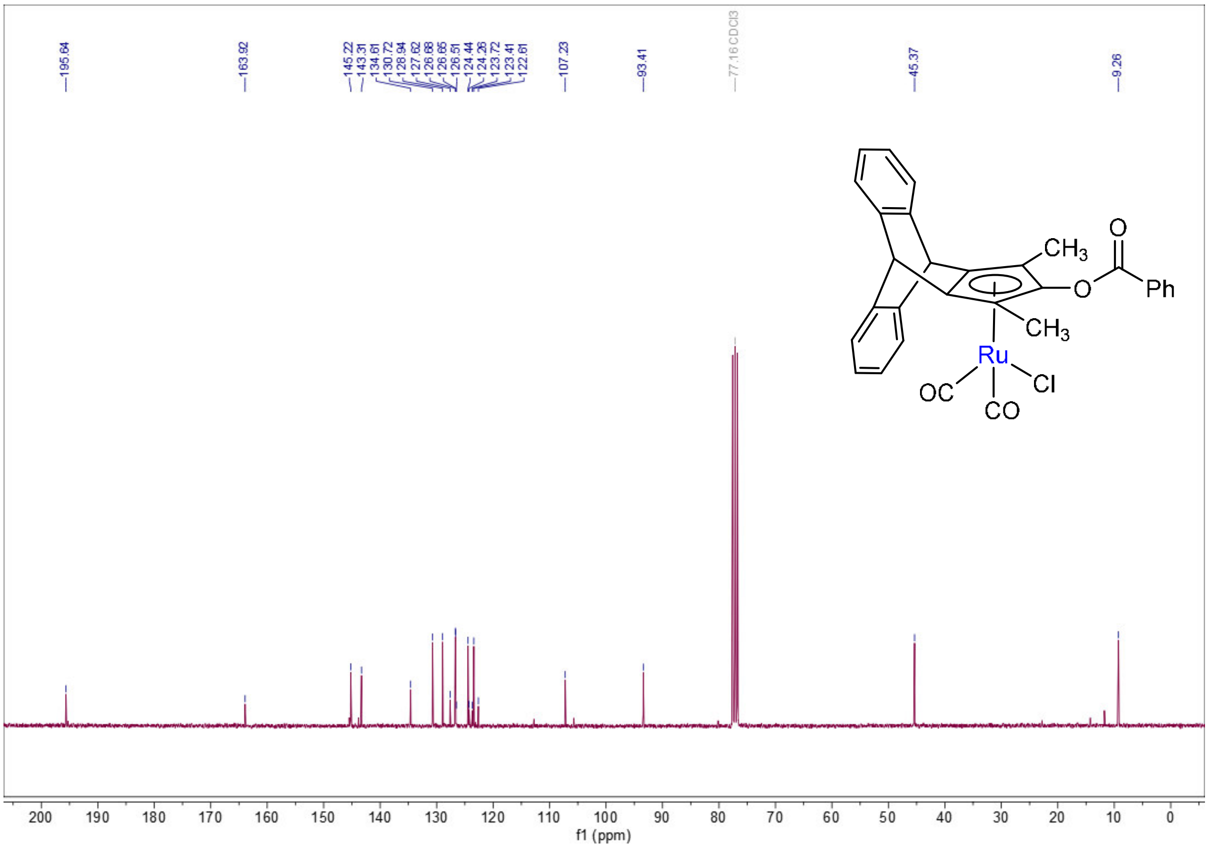

Figure S-43: ^{13}C-APT NMR ($CDCl_3$, 125 MHz) of **14**

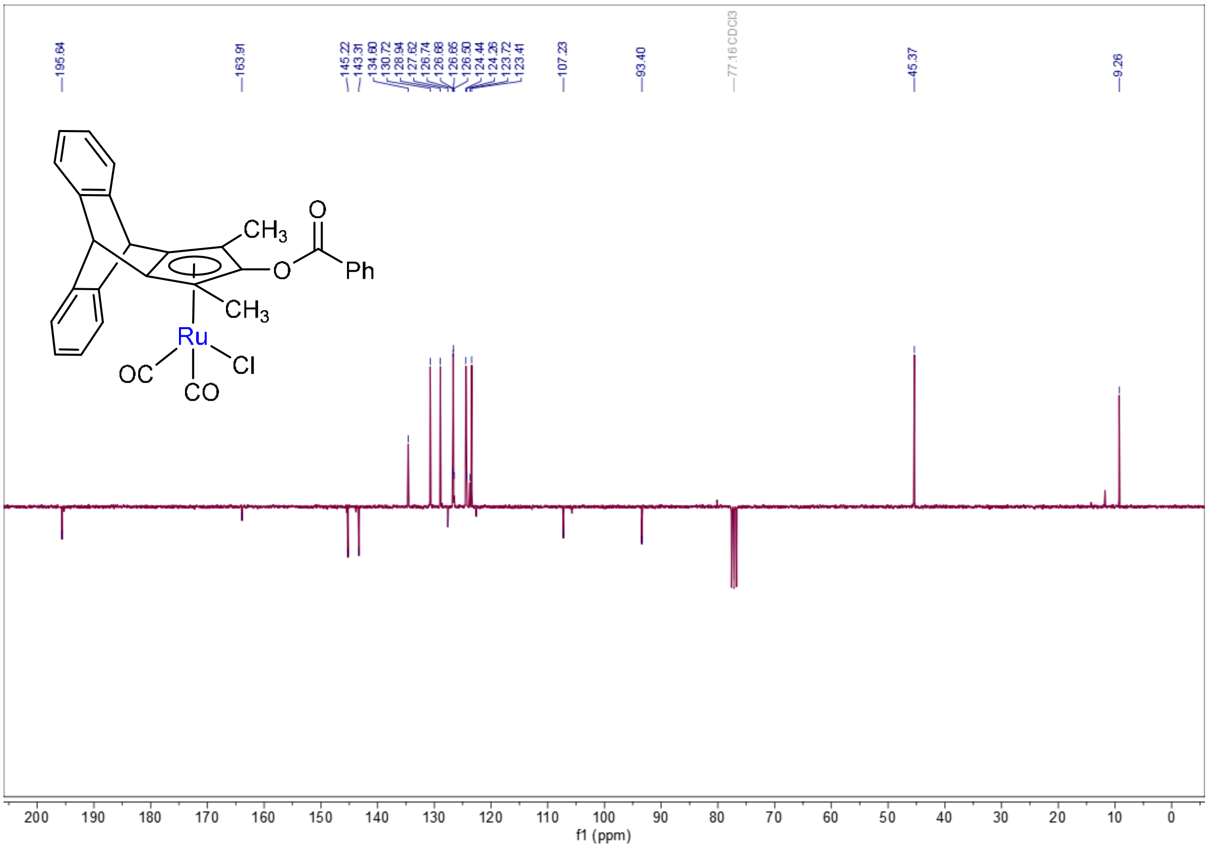

Figure S-44: H,H-COSY ($CDCl_3$, 300 MHz) of **14**

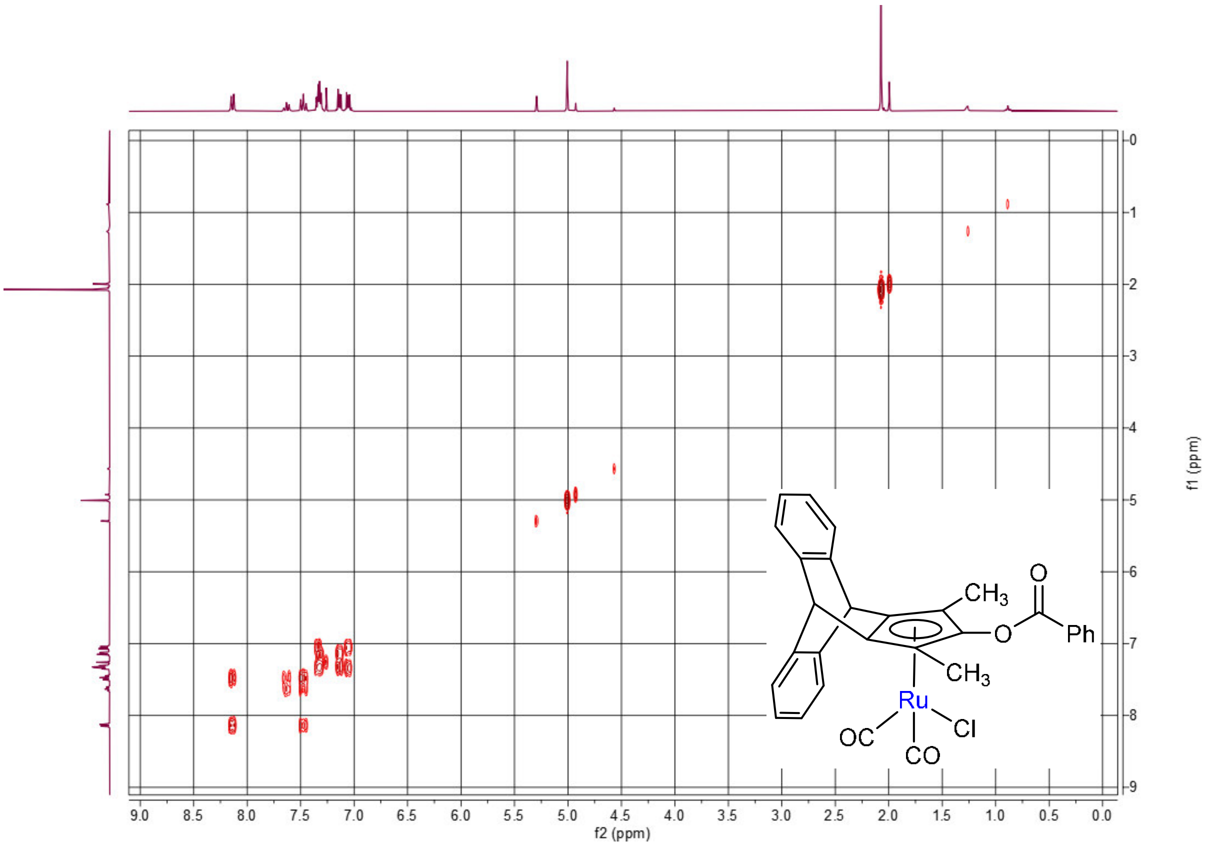

Figure S-45: HSQC ($CDCl_3$, 300 MHz) of **14**

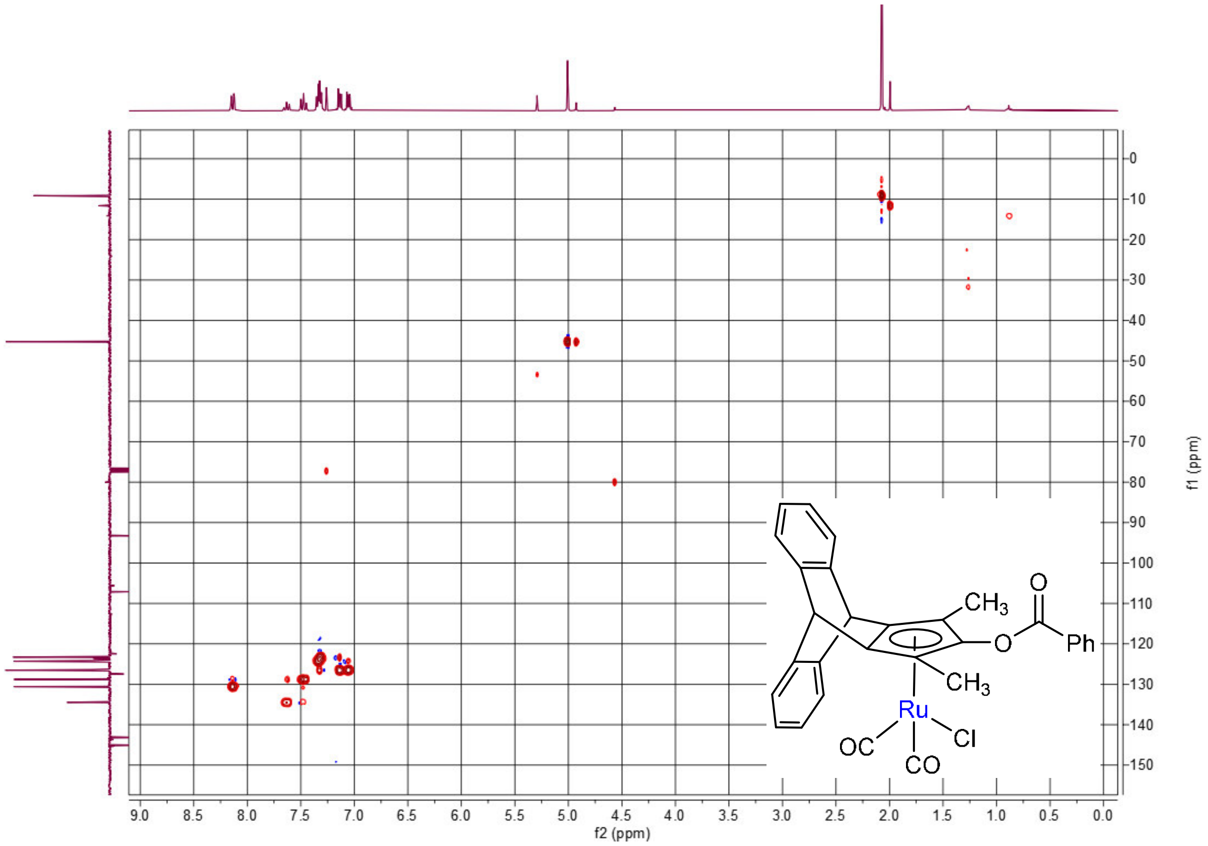

Figure S-46: ^{1}H NMR ($CDCl_3$, 300 MHz) of **15**

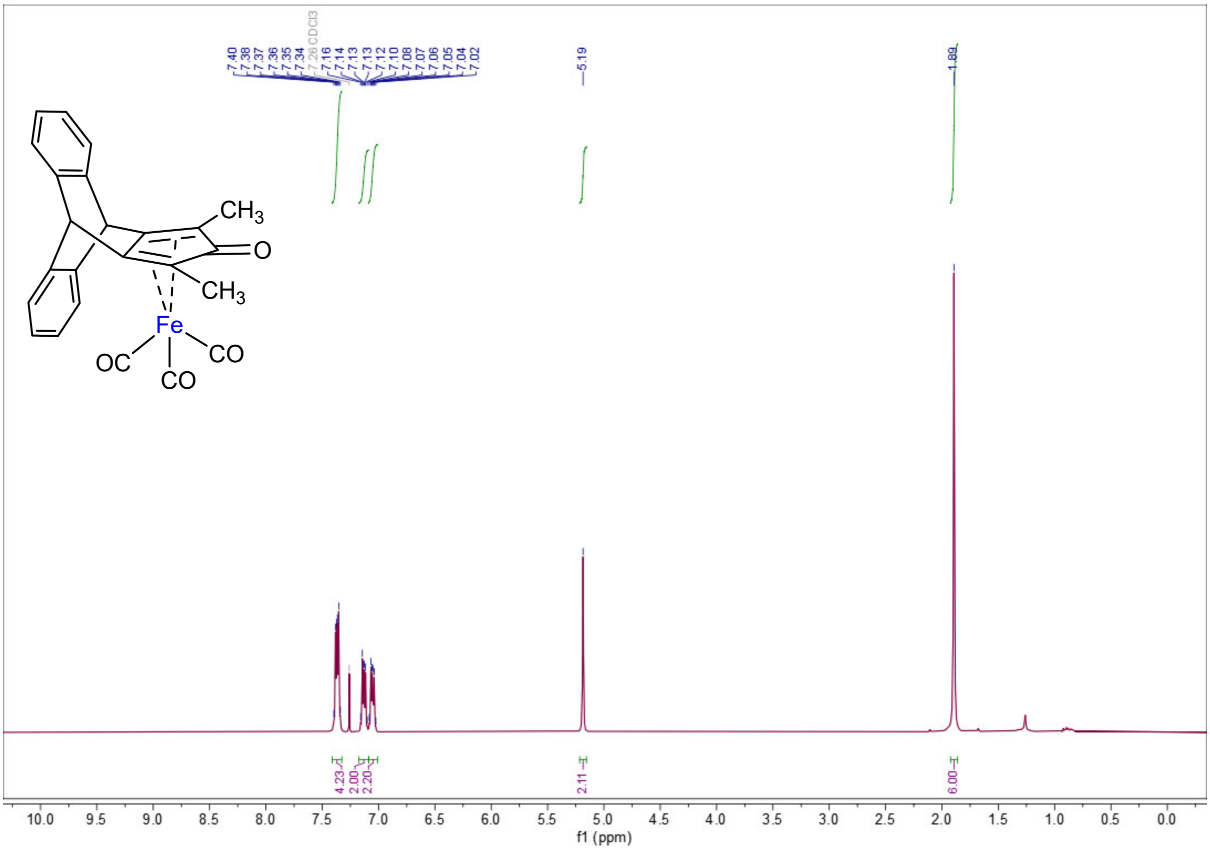

Figure S-47: ^{13}C{^{1}H} NMR ($CDCl_3$, 125 MHz) of **15**

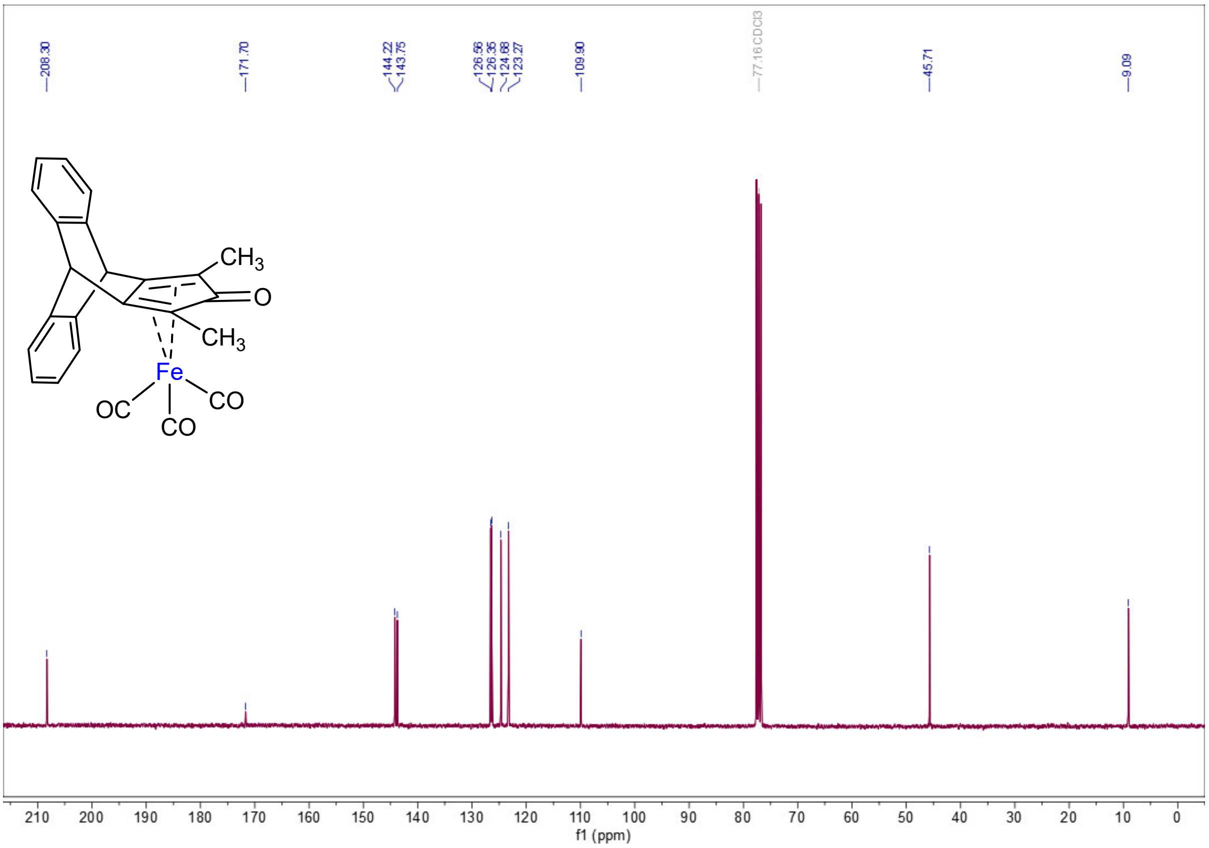

Figure S-48: ^{13}C-APT NMR (CDCl$_3$, 125 MHz) of **15**

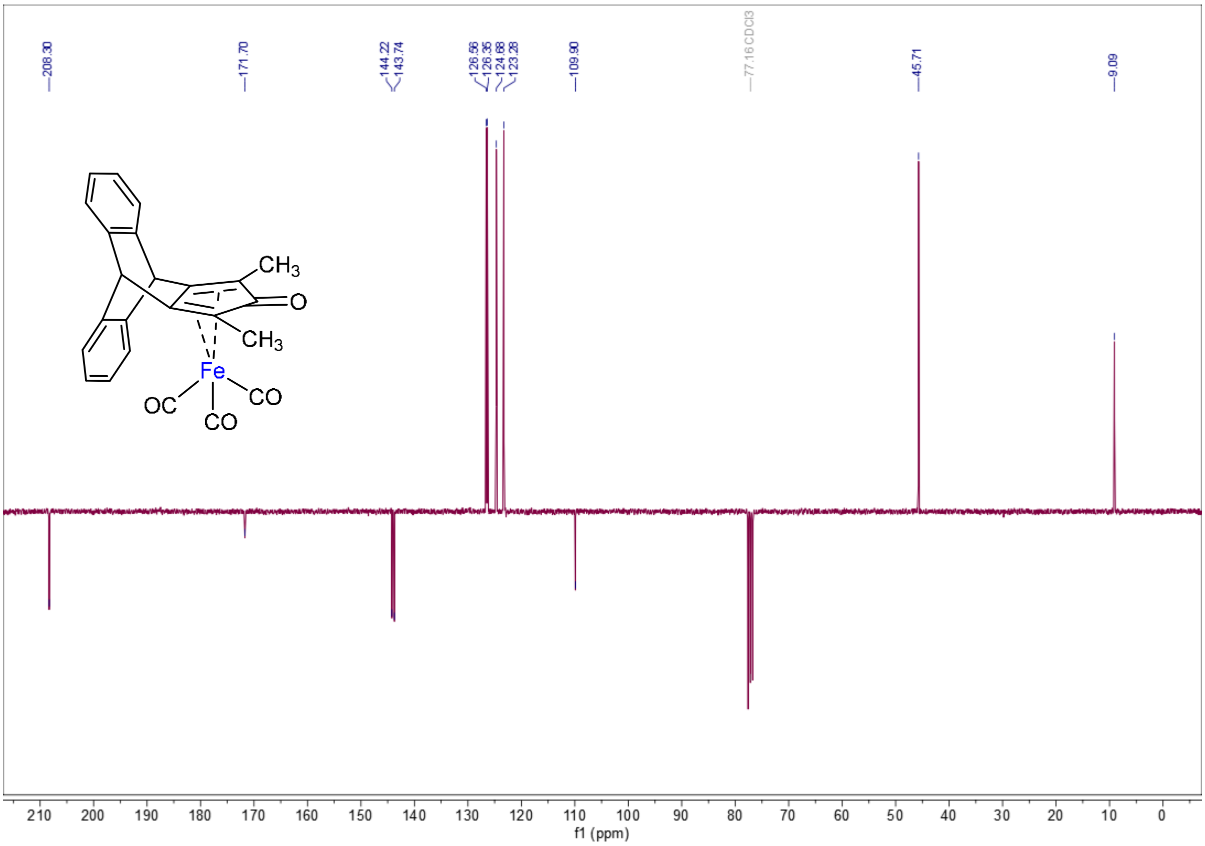

Figure S-49: H,H-COSY (CDCl$_3$, 300 MHz) of **15**

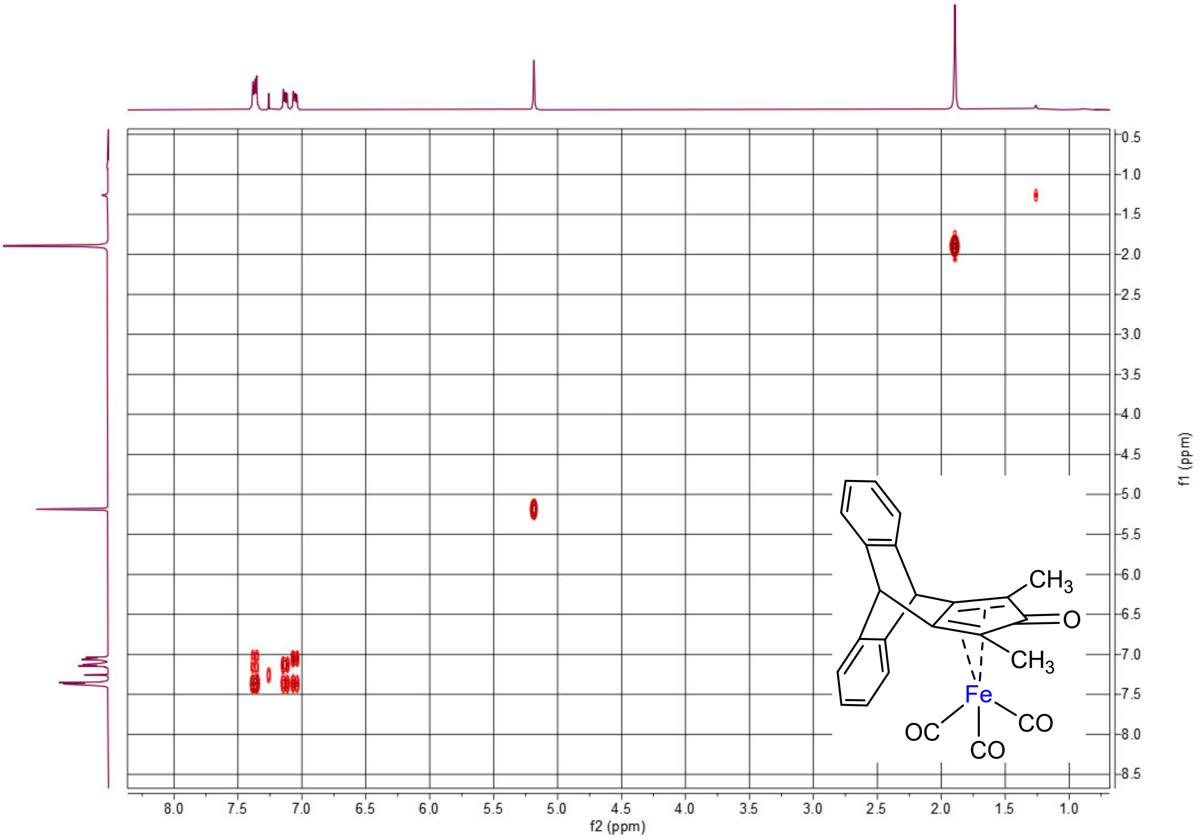

Figure S-50: HSQC ($CDCl_3$, 300 MHz) of **15**

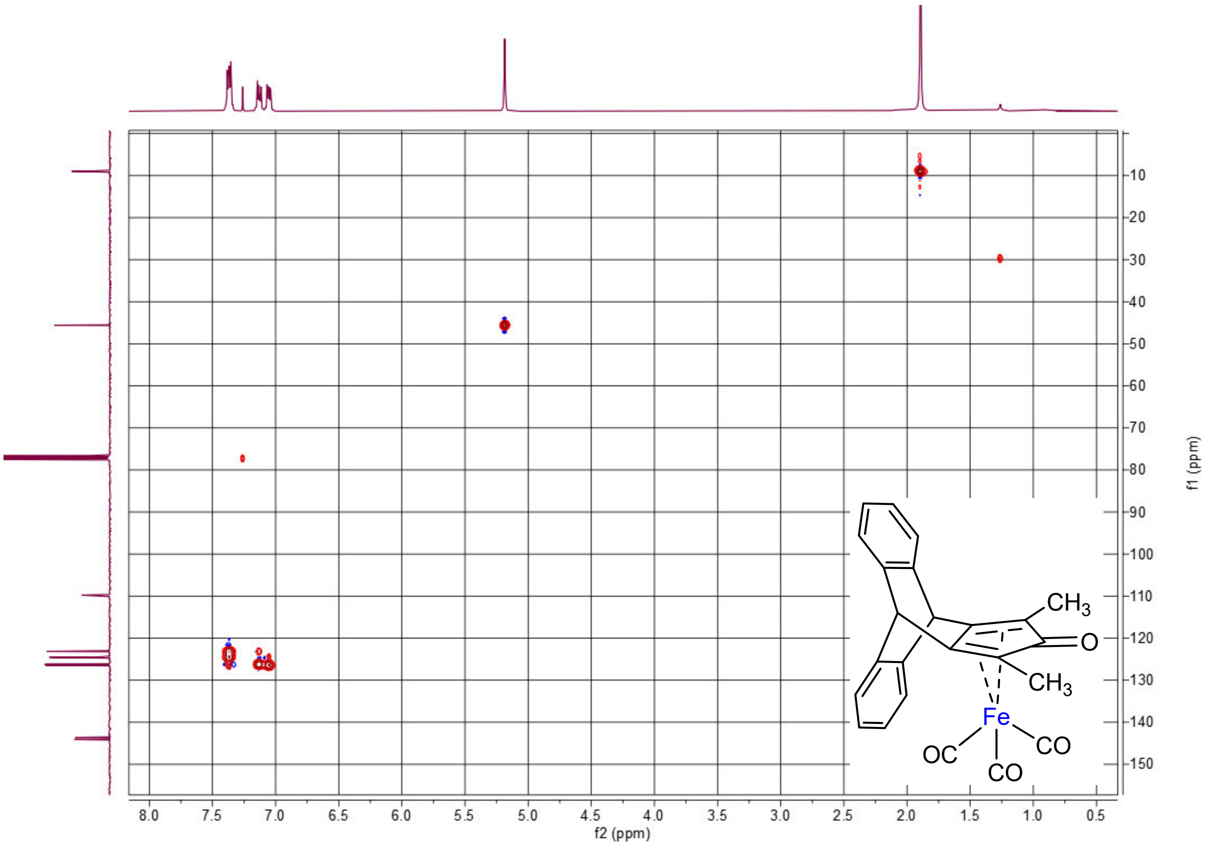

Figure S-51: ^{1}H NMR ($CDCl_3$, 300 MHz) of **16**

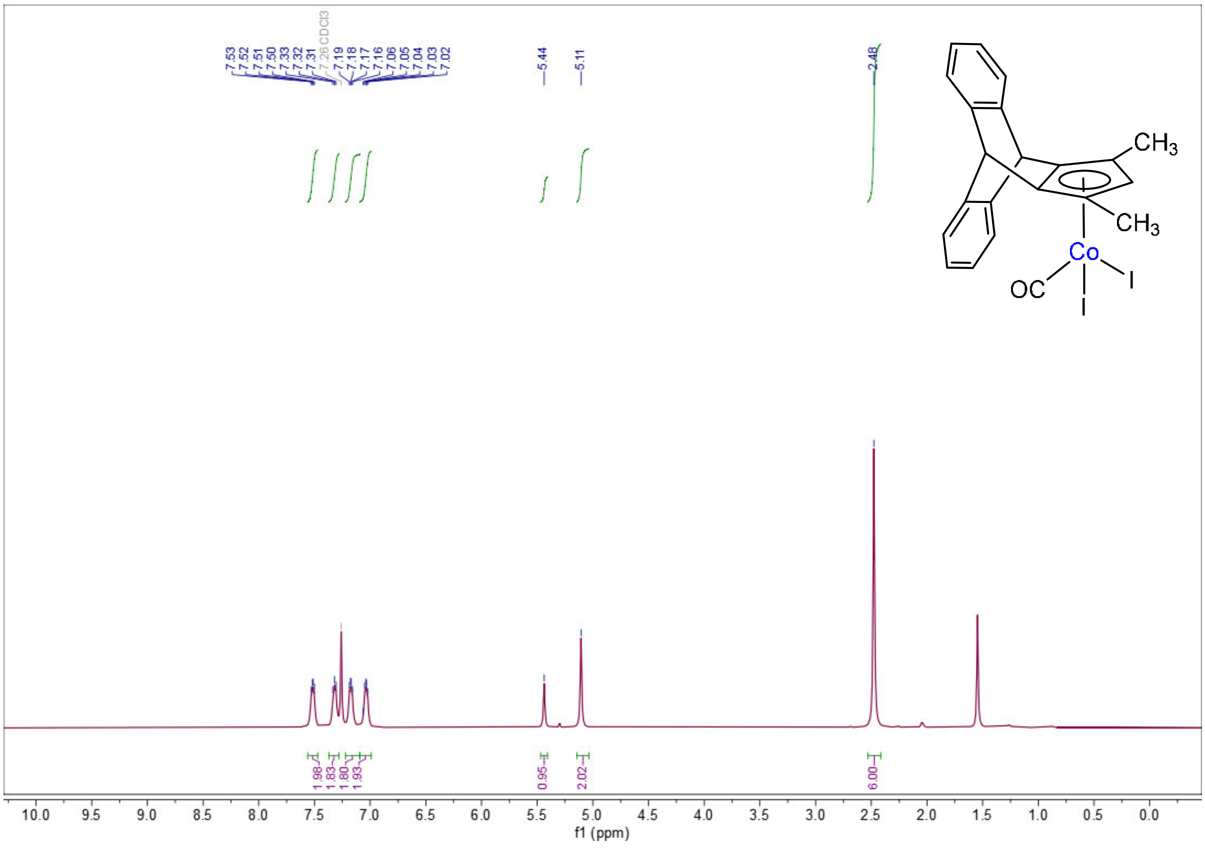

Figure S-52: $^{13}C\{^{1}H\}$ NMR ($CDCl_3$, 125 MHz) of **16**

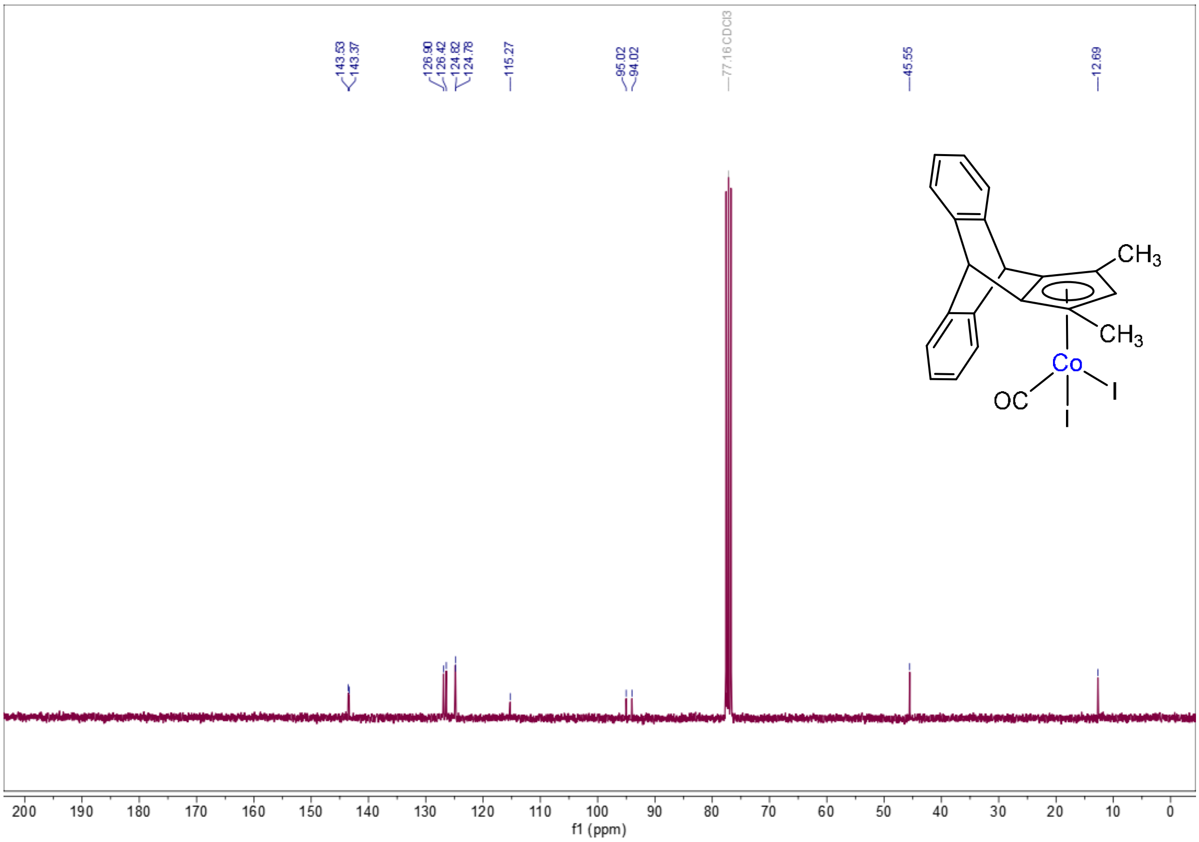

Figure S-53: H,H-COSY ($CDCl_3$, 300 MHz) of **16**

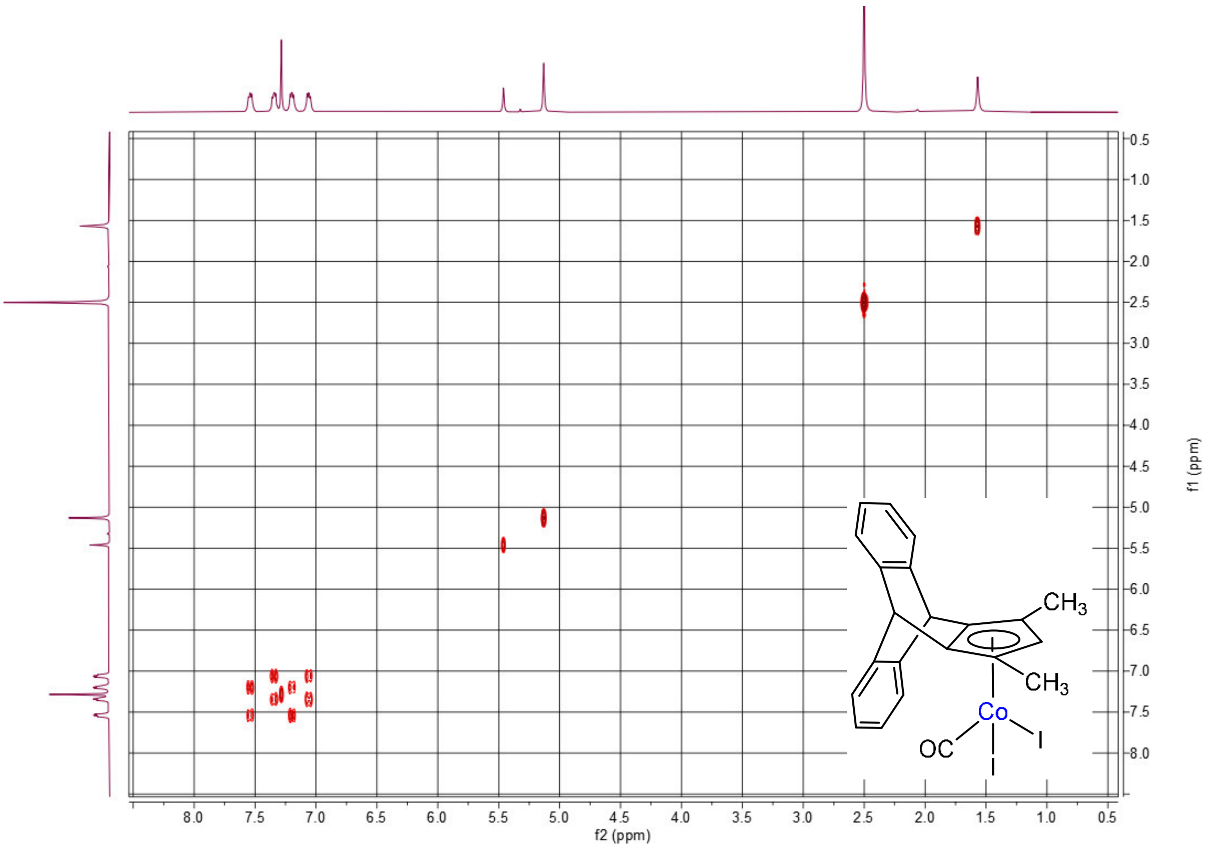

Figure S-54: HSQC ($CDCl_3$, 300 MHz) of **16**

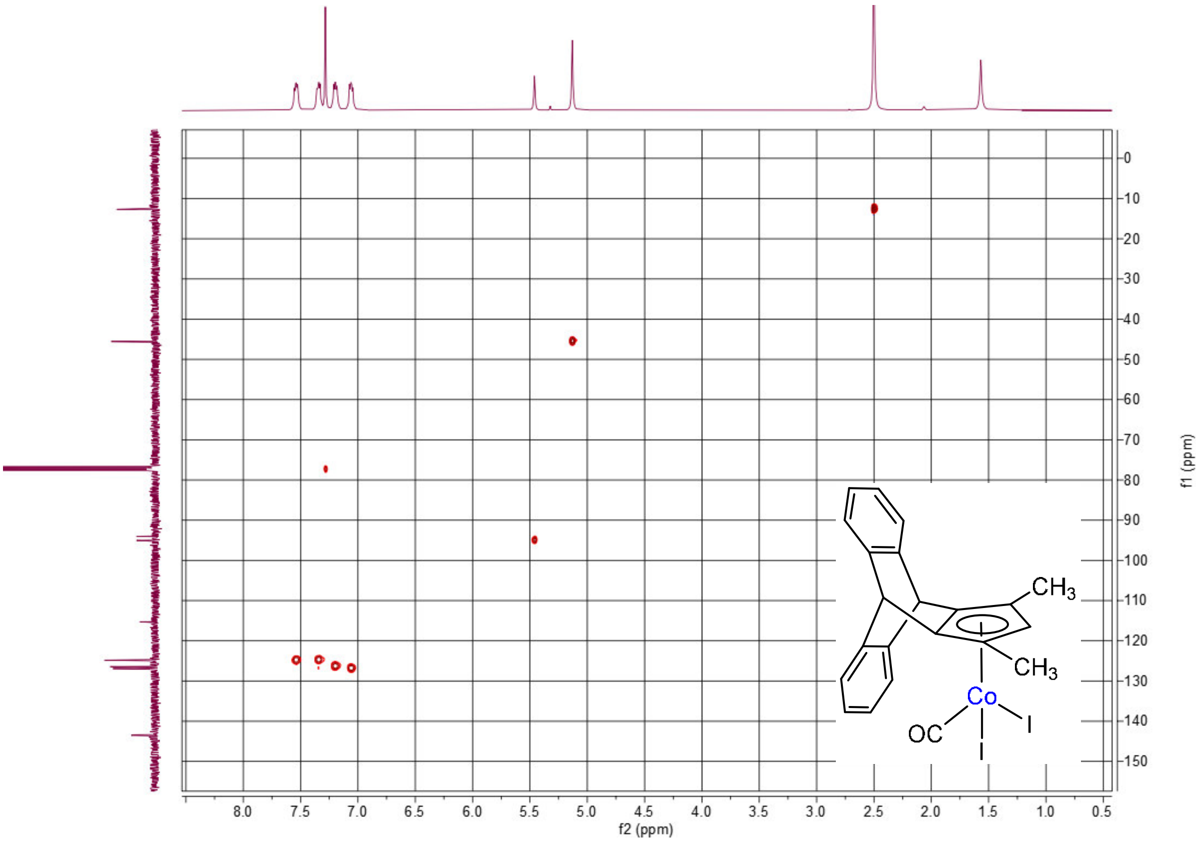

Figure S-55: ^{1}H NMR ($CDCl_3$, 300 MHz) of **18a**

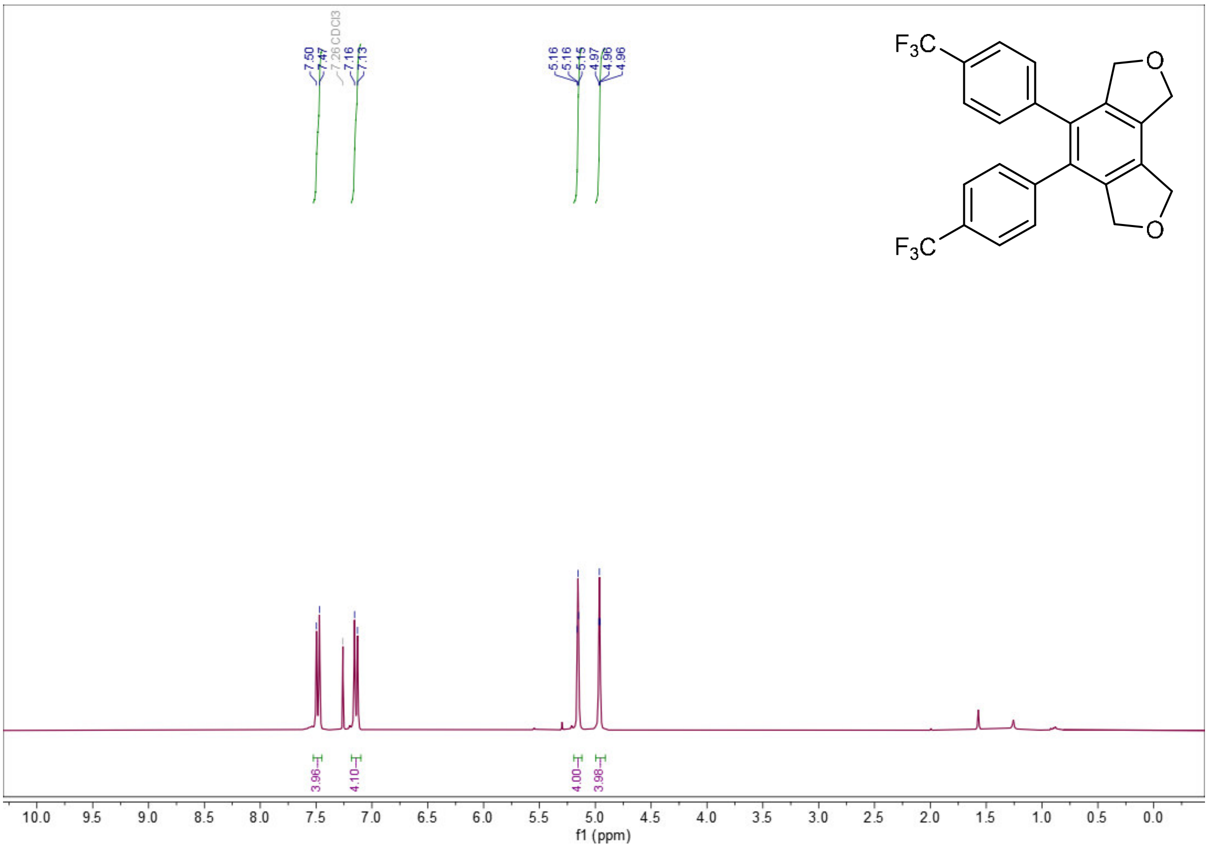

Figure S-56: ^{19}F NMR ($CDCl_3$, 125 MHz) of **18a**

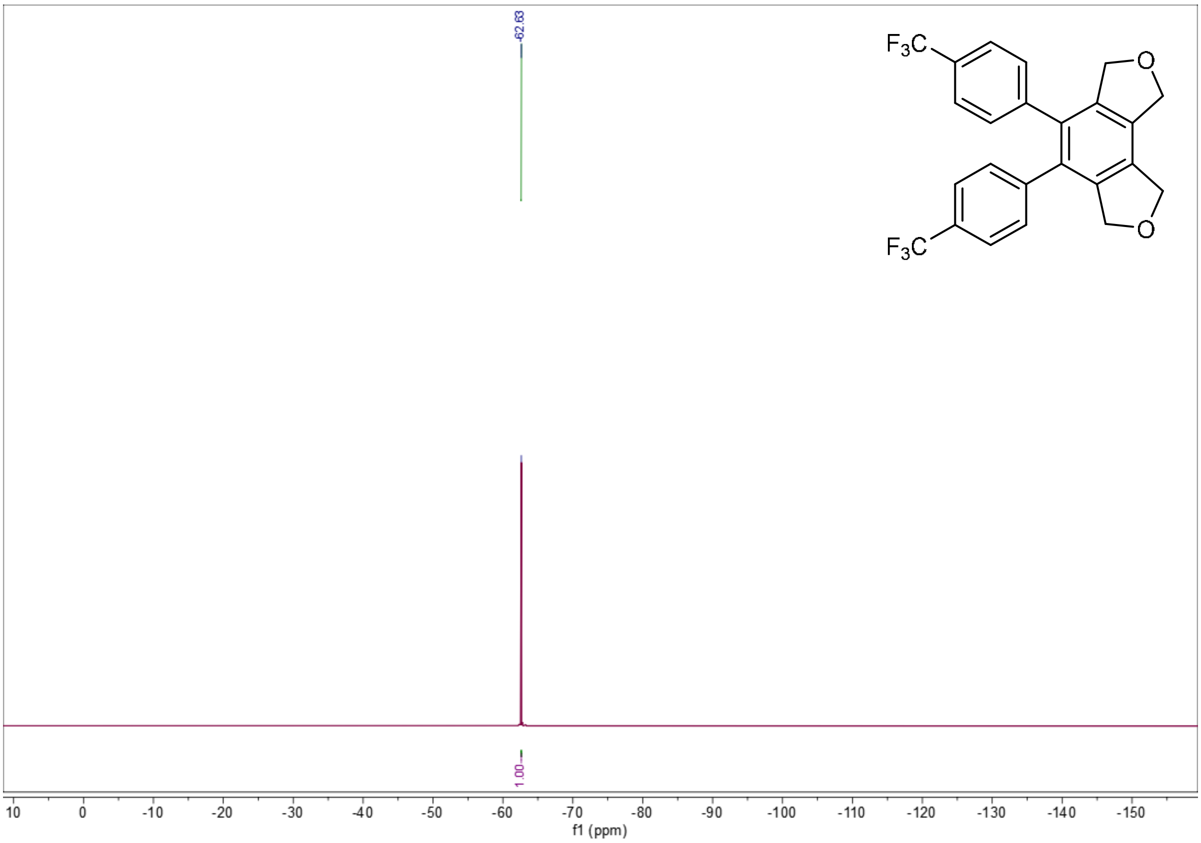

Figure S-57: ^{13}C{^{1}H} NMR ($CDCl_3$, 125 MHz) of **18a**

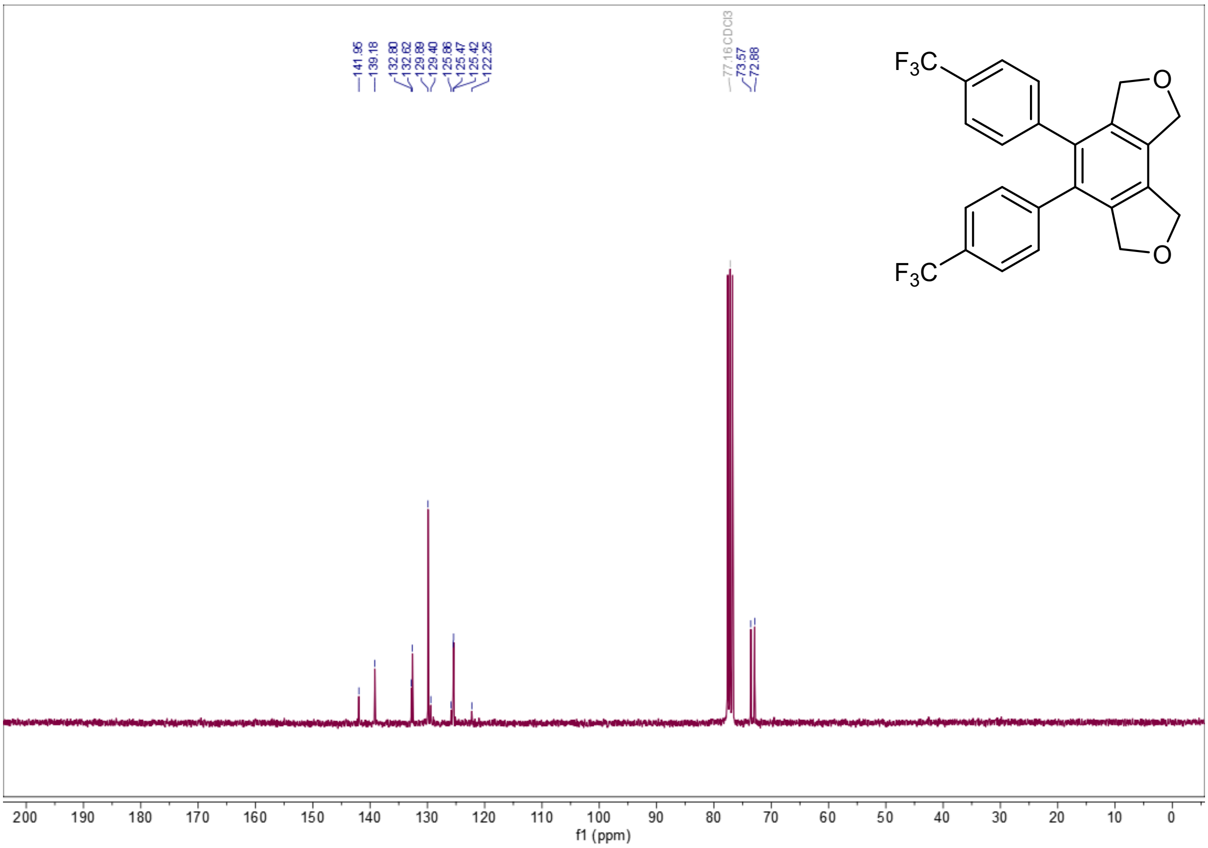

Figure S-58: ^{1}H NMR ($CDCl_3$, 300 MHz) of **18b**

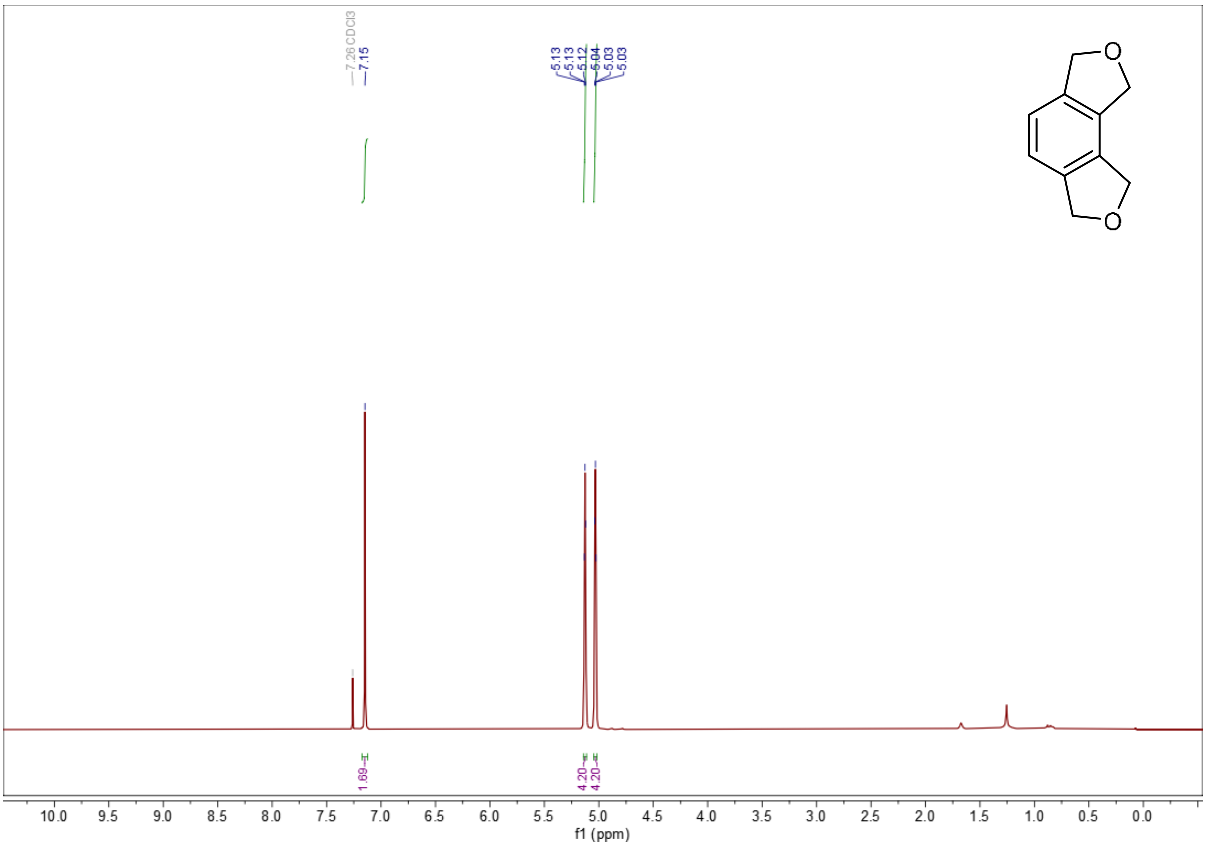

Figure S-59: $^{13}C\{^{1}H\}$ NMR ($CDCl_3$, 125 MHz) of **18b**

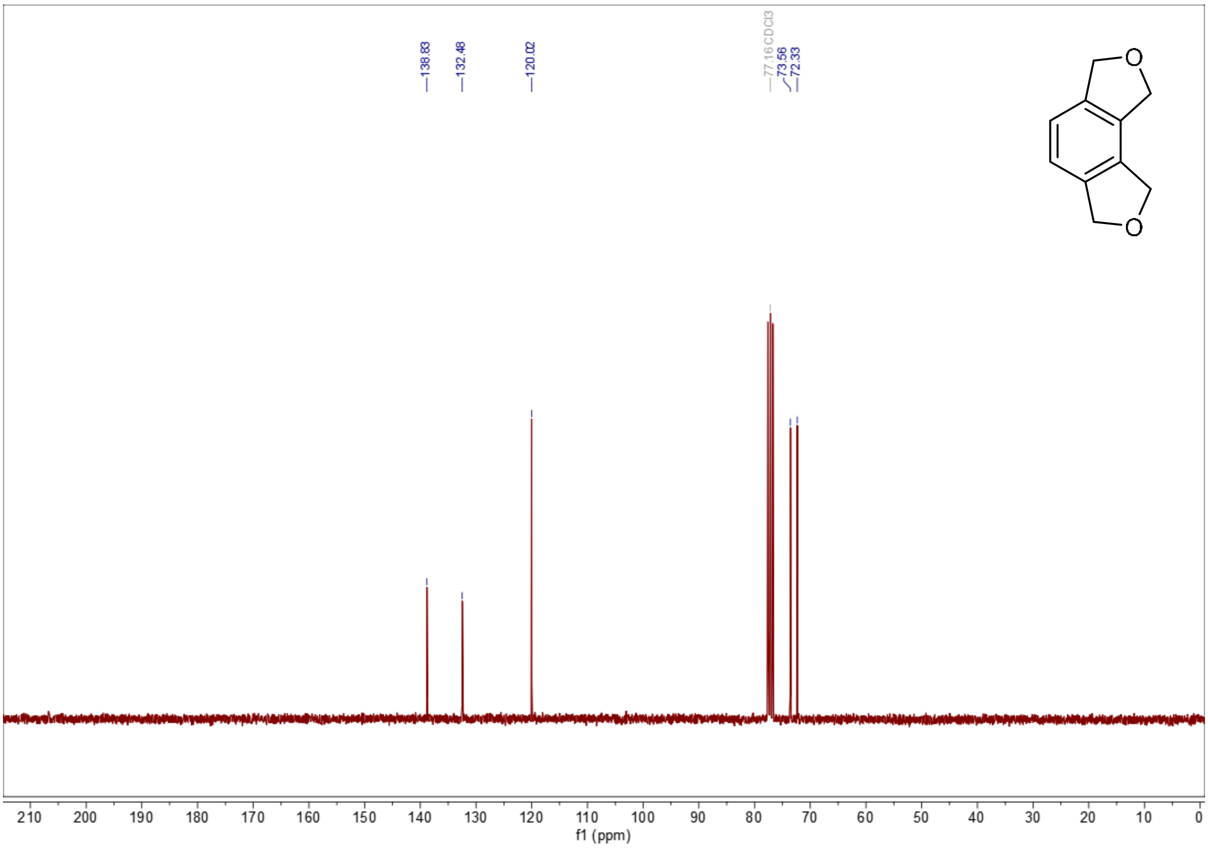

Figure S-60: ^{1}H NMR ($CDCl_3$, 300 MHz) of **18c**

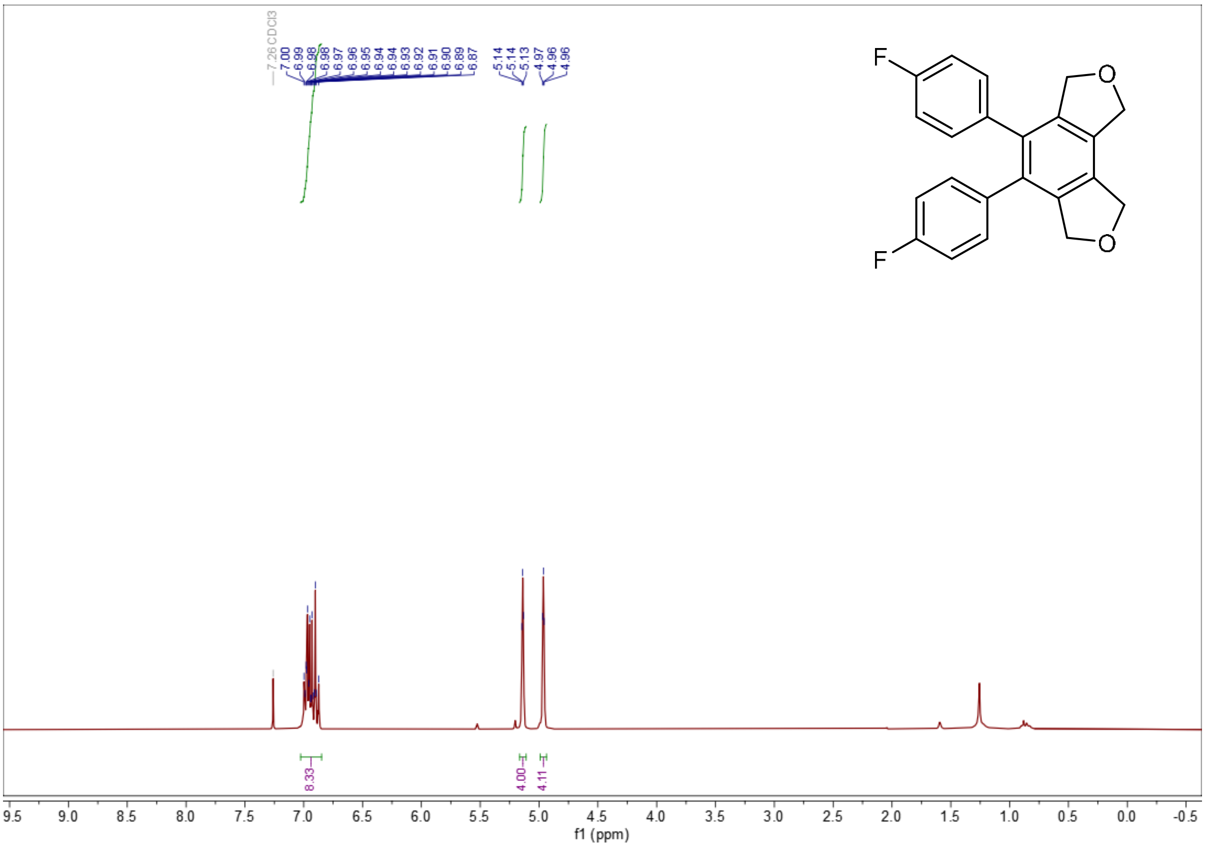

Figure S-61: ^{19}F NMR ($CDCl_3$, 300 MHz) of **18c**

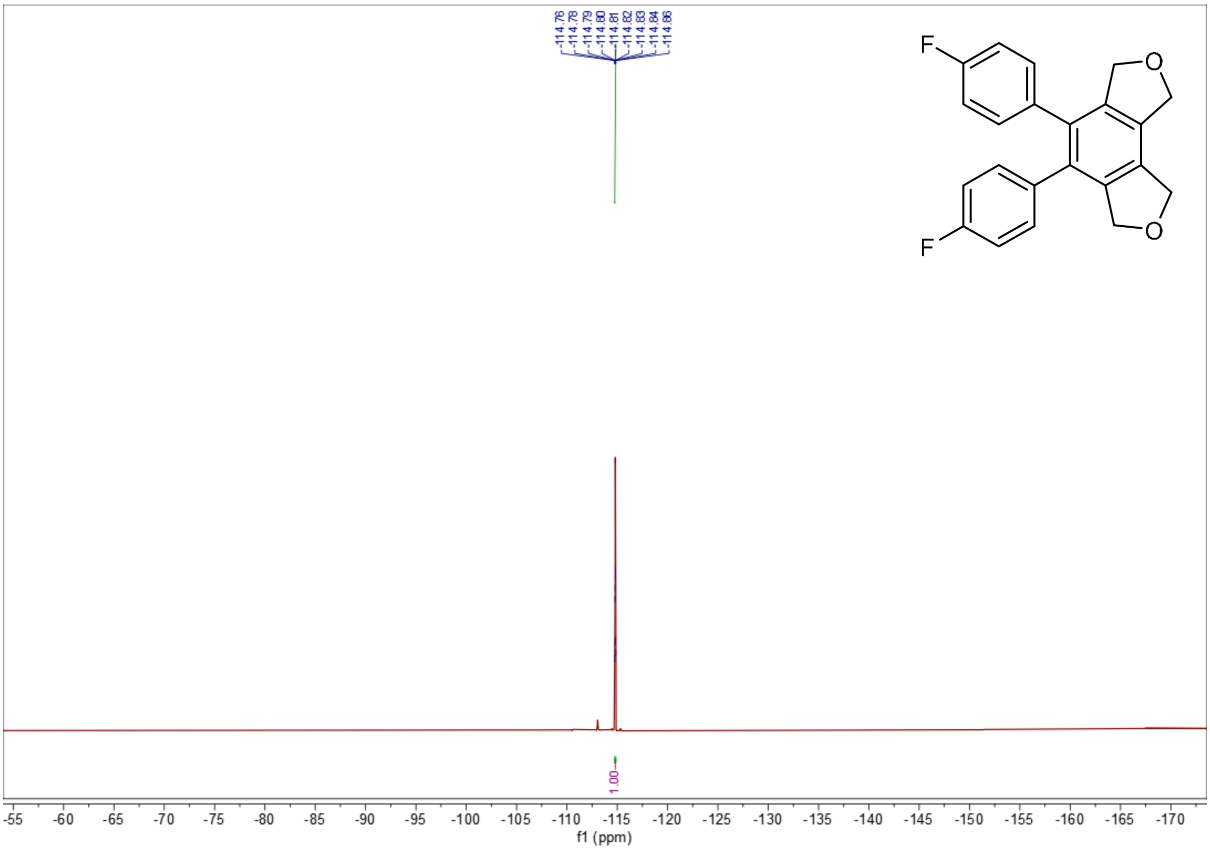

Figure S-62: ^{1}H NMR ($CDCl_3$, 300 MHz) of **20a**

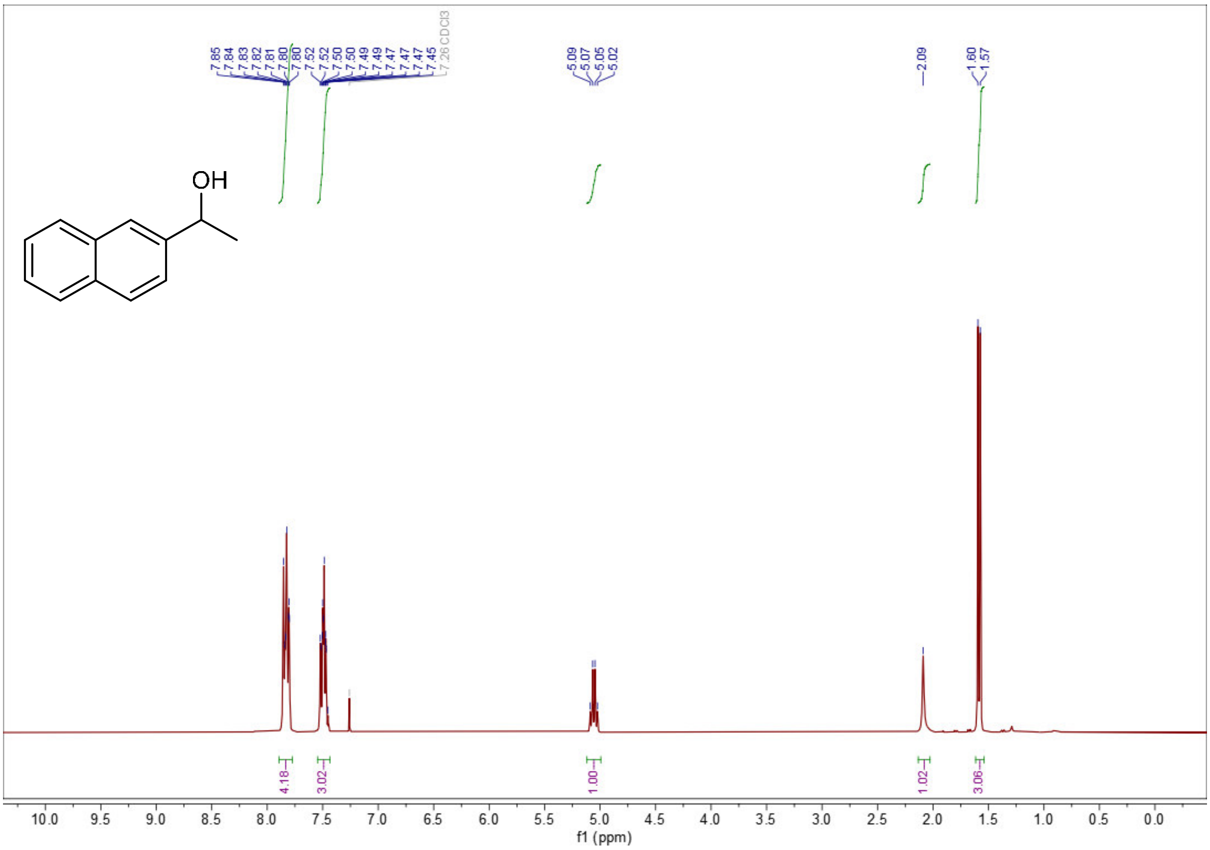

Figure S-63: ^{13}C{^{1}H} NMR ($CDCl_3$, 125 MHz) of **20a**

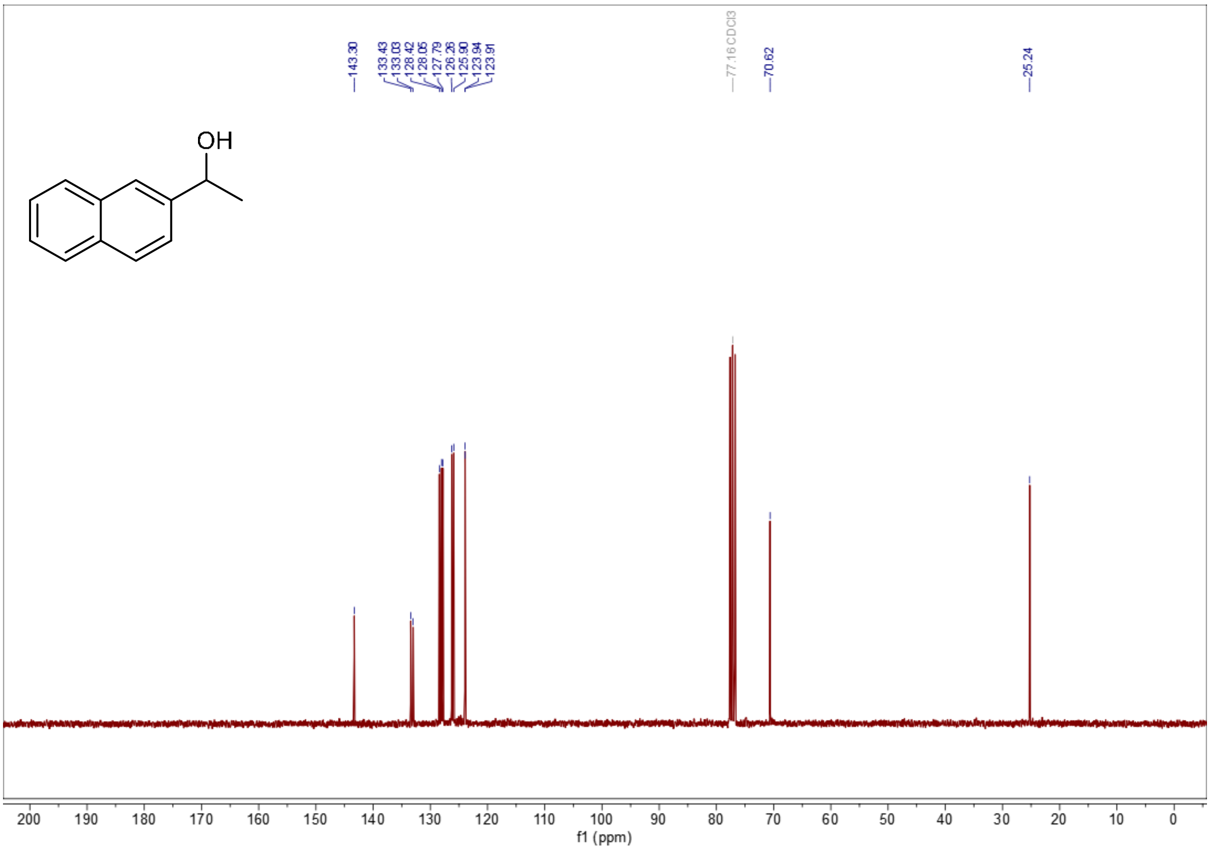

Figure S-64: ^{1}H NMR ($CDCl_3$, 300 MHz) of **20c**

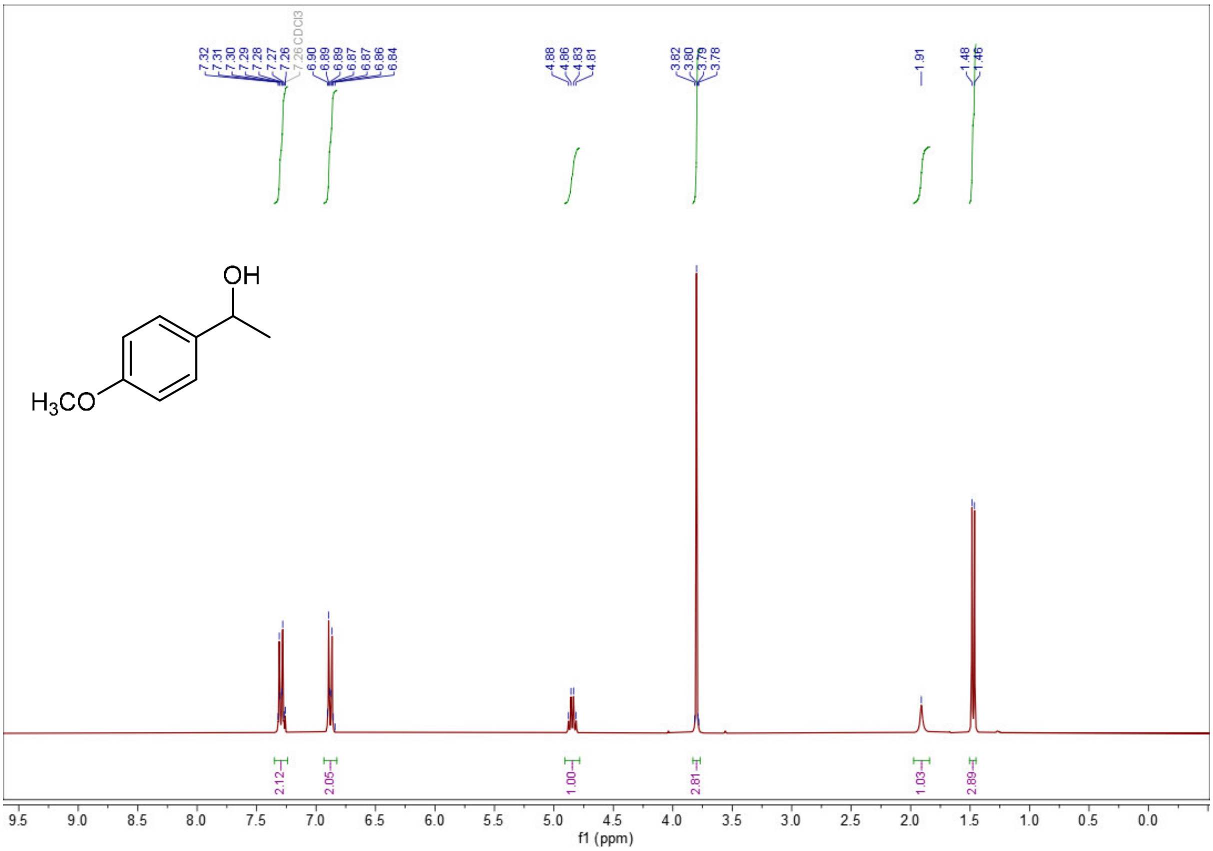

Figure S-65: ^{1}H NMR ($CDCl_3$, 300 MHz) of **23a**

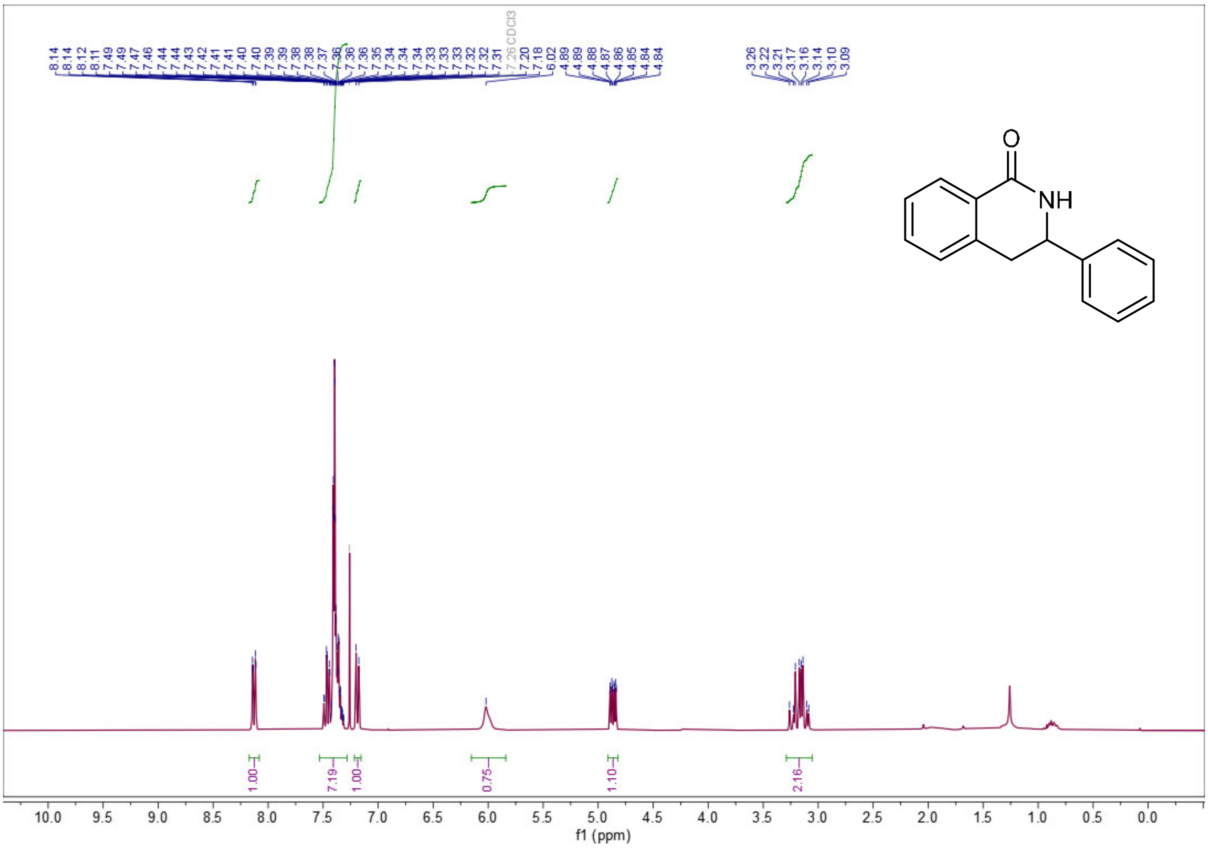

Figure S-66: $^{13}C\{^{1}H\}$ NMR ($CDCl_3$, 125 MHz) of **23a**

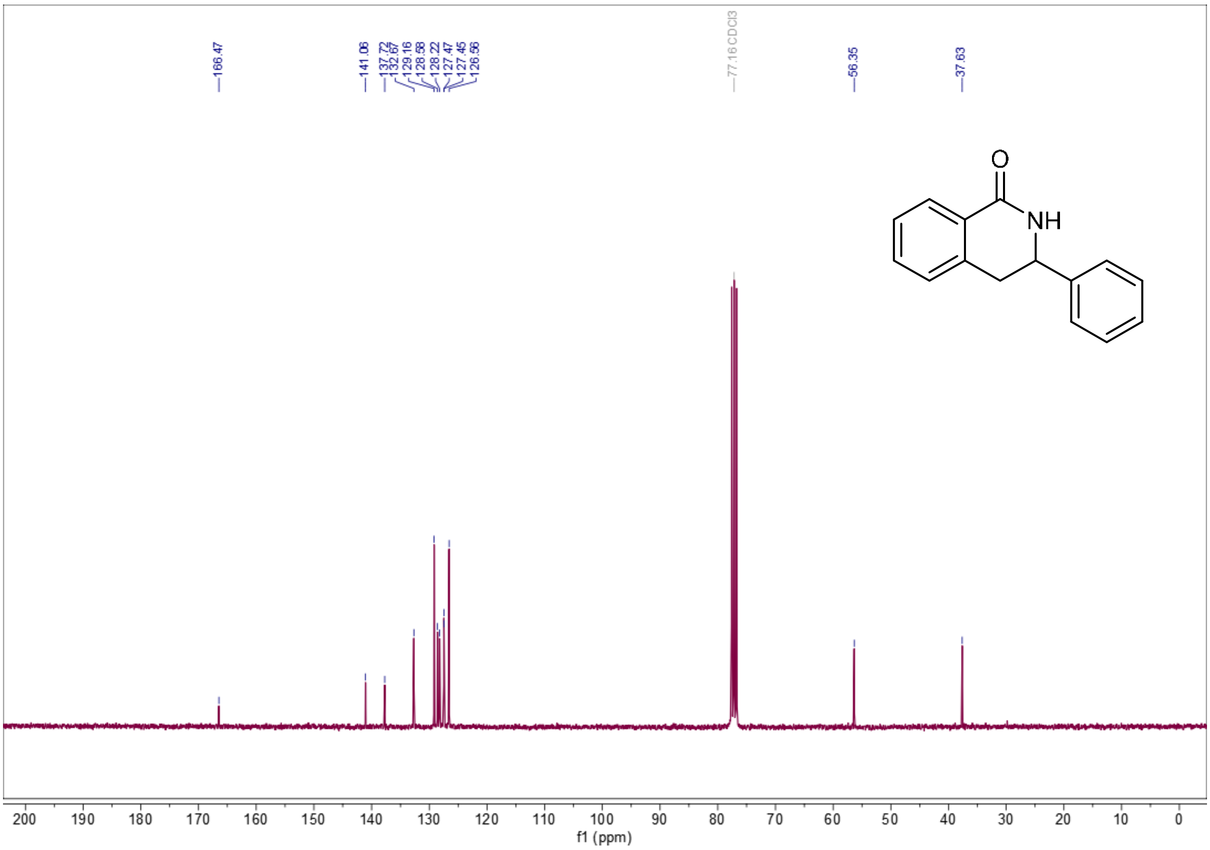

Figure S-67: ^{1}H NMR ($CDCl_3$, 300 MHz) of **23b**

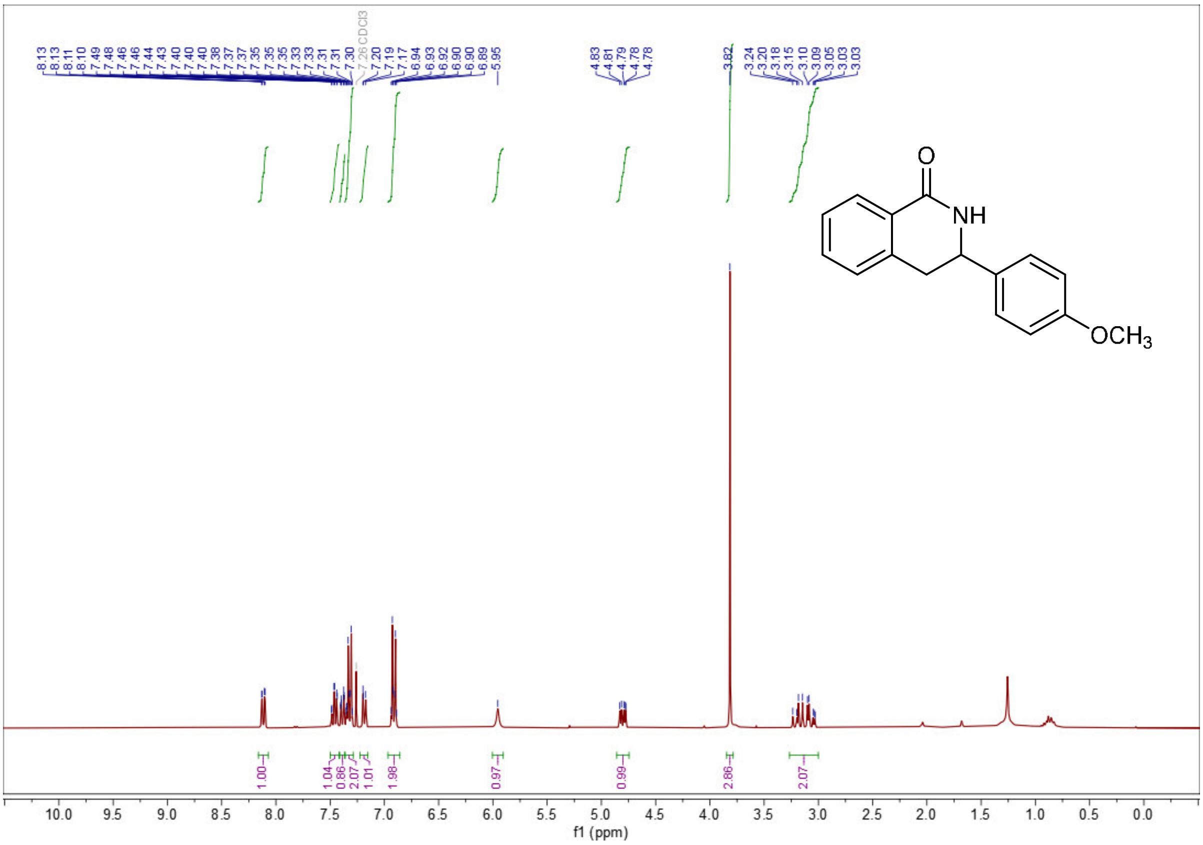

Figure S-68: $^{13}C\{^1H\}$ NMR ($CDCl_3$, 125 MHz) of **23b**

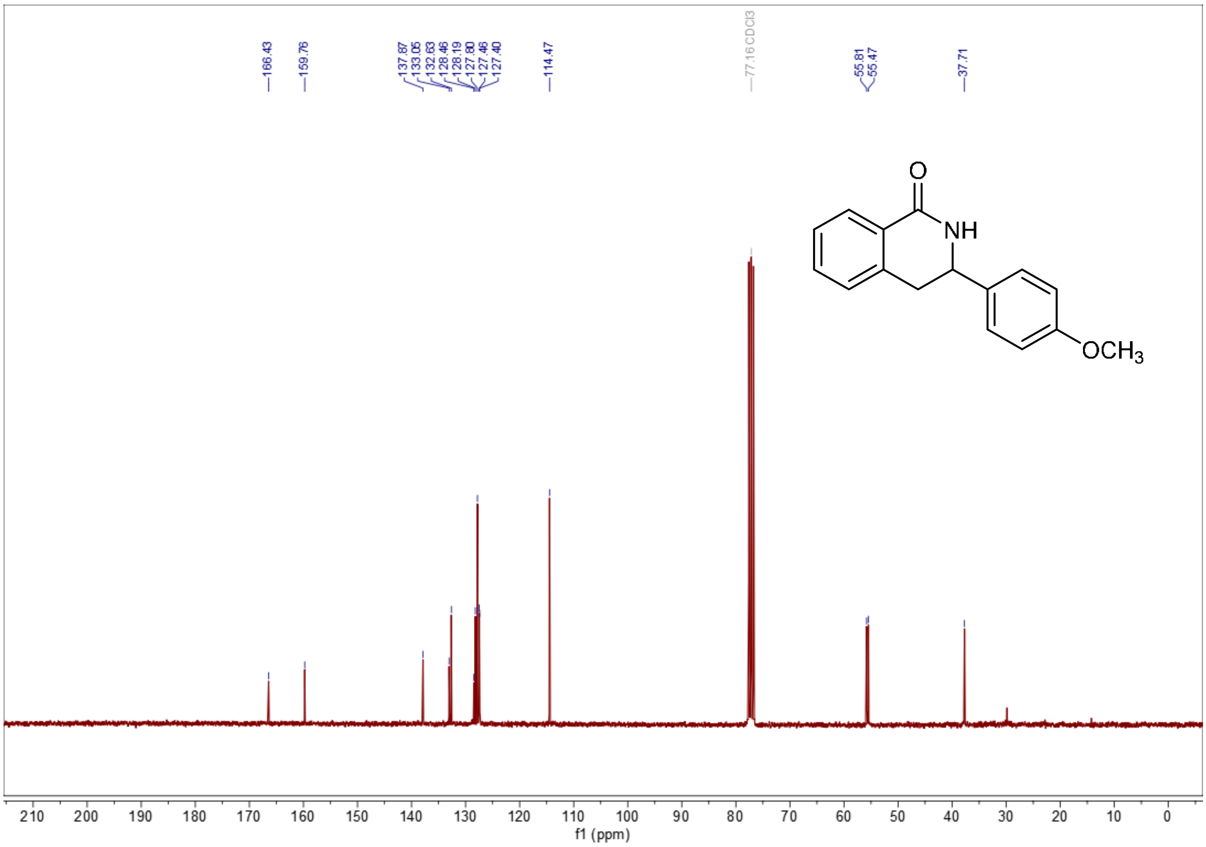

Figure S-69: ^1H NMR ($CDCl_3$, 300 MHz) of **23c**

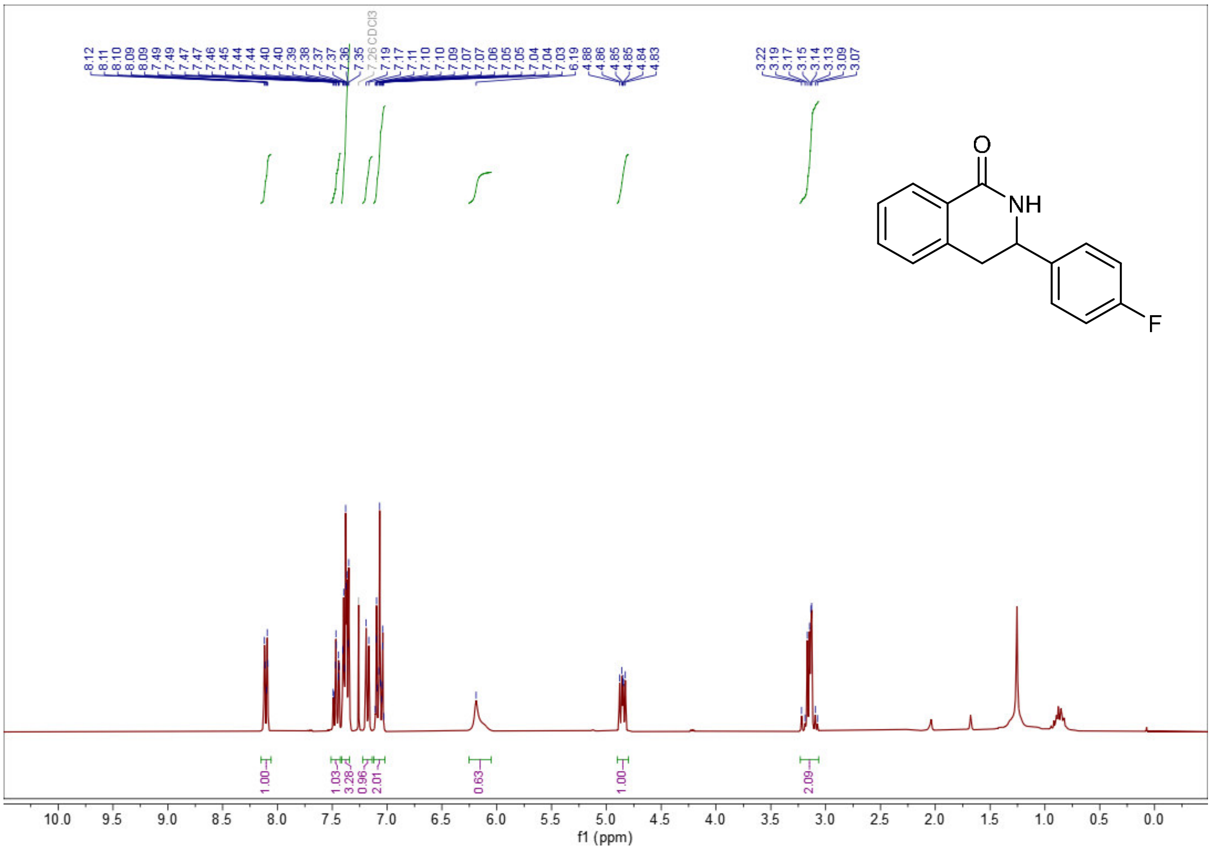

Figure S-70: ^{19}F NMR ($CDCl_3$, 125 MHz) of **23c**

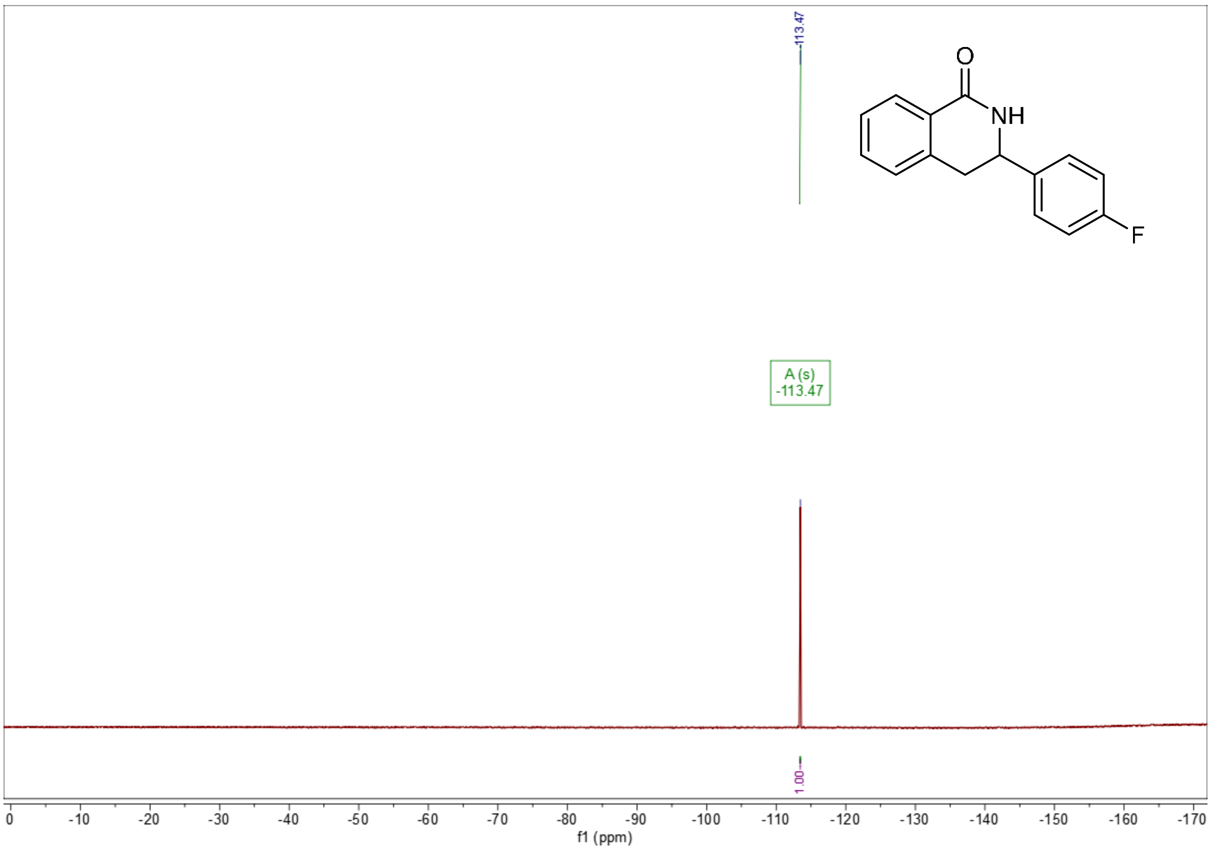

Figure S-71: $^{13}C\{^1H\}$ NMR ($CDCl_3$, 125 MHz) of **23c**

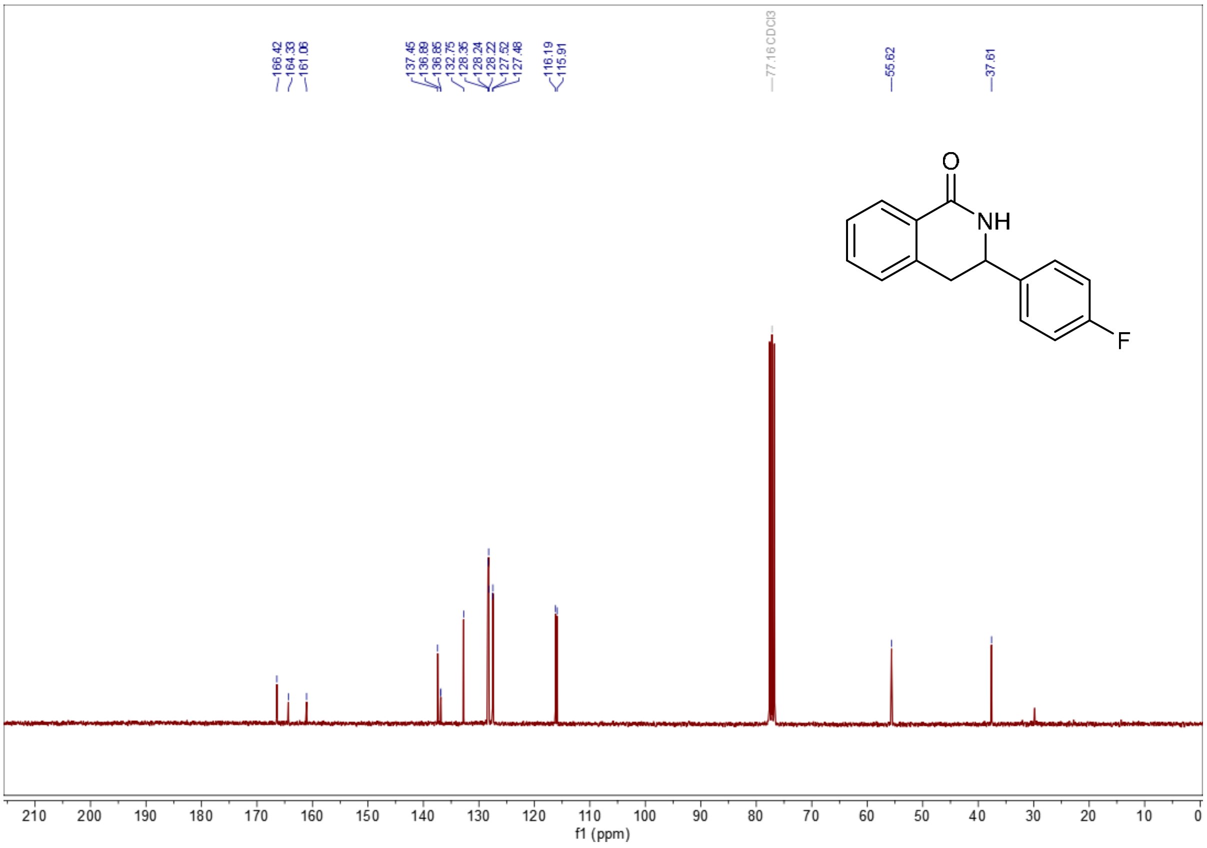

4. References

(1) Jia, J.; Yamaguchi, Y.; Ueda, T.; Yamada, H.; Kakiuchi, K.; Morimoto, T. Rhodium(I)-Catalyzed [2+2+1]-Carbonylative Cycloaddition of Diynes with Anthracene α-Diketone as the Source of CO. *Synlett* **2022**, *33*, 1948-1952.

(2) (a) Wang, S.; Tang, M.; Wu, L.; Bian, L.; Jiang, L.; Liu, J.; Tang, Z.; Liang, Y.: Liu, Z. Linear Nonalternant Isomers of Acenes Fusing Multiple Azulene Units. *Angew. Chem. Int. Ed.* **2022**, *61*, e202205658. (b) Xie, Y.; Gong, Y.; Han, M.; Zhang, F.; Peng, Q.; Xie, G.; Li, Z. Tetraphenylcyclopentadiene-Based Hyperbranched Polymers: Convenient Syntheses from One Pot ″A_4+B_2″ Polymerization and High External Quantum Yields up to 9.74% in OLED Devices. *Macromolecules* **2019**, *52*, 896-903.

(3) G. M. Sheldrick, A short history of SHELX. *Acta Cryst. A* **2008**, *64*, 112-122.

(4) G. M Sheldrick, Crystal structure refinement with SHELXL, *Acta Cryst. C* **2015**, *71*, 3-8.
